# Erinacenones M–Y, Part 2: Thirteen New Isoindolinone Alkaloids with α-Glucosidase Inhibitory Activity from Liquid Cultures of the Edible and Medicinal Mushroom *Hericium erinaceus*

**DOI:** 10.3390/molecules31183333

**Published:** 2026-09-20

**Authors:** Lin-Lin Yuan, Ji-Kai Liu

**Affiliations:** 1 College of Food Science and Engineering, Tarim University, Alar 843300, China; 2 Anhui Province Key Laboratory of Bioactive Natural Products, School of Pharmacy, Anhui University of Chinese Medicine, Hefei 230012, China

**Keywords:** mushroom, *Hericium erinaceus*, Hericiaceae, isoindolinone alkaloids, α-glucosidase inhibitory activity

## Abstract

As a continuation of our previous study on erinacenones A–L (Part 1), thirteen previously undescribed isoindolin-1-ones, erinacenones M–Y (**1**–**13**), were isolated from the fermentation broth of *Hericium erinaceus*. Their structures were elucidated by extensive spectroscopic analyses (1D and 2D NMR, HRESIMS), and the absolute configurations of **1**–**13** were determined by comparing their specific rotations with those of related phthalimidines (**14**–**23**). Compound **11** showed the highest α-glucosidase inhibitory activity among the tested compounds, with 31.5% inhibition at 50 μM. These findings significantly enrich the chemical diversity of isoindolinone metabolites from *H. erinaceus* and provide a foundation for future structure–activity relationship studies.

## 1. Introduction

Diabetes mellitus is one of the most prevalent chronic metabolic diseases worldwide, posing a major threat to human health. According to the International Diabetes Federation (IDF), the global number of patients reached 536 million in 2021 and is projected to rise to 783 million by 2045, with nearly half of cases remaining undiagnosed [1,2]. Type 2 diabetes (T2D) accounts for the vast majority of cases. Although commonly used clinical drugs such as acarbose, an α-glucosidase inhibitor, effectively control postprandial hyperglycemia, they are often associated with gastrointestinal side effects [3]. Hence, identifying alternative inhibitors with high efficacy and low toxicity from natural sources has attracted considerable interest.

Fungi are a vital source of bioactive natural products. *Hericium erinaceus*, a medicinal and edible fungus, has been used in traditional Chinese medicine for over a millennium to treat gastric disorders [4] and produces diterpenoids, sterols, polysaccharides, and aromatic compounds. Among these, isoindolinone alkaloids are distinguished by their unique aromatic skeleton and have been reported to exhibit nerve growth factor-stimulating [5,6,7,8], cytotoxic [9,10,11], and anticoagulant activities [12]. Our previous work on aromatic metabolites from *H. erinaceus* led to the isolation of erinacenones A–L, which exhibited cytotoxic activities against cancer cell lines [13].

Recently, their hypoglycemic potential has been unveiled. Lee et al. [14] isolated 4-[3′,7′-dimethyl-2′,6′-octadienyl]-2-formyl-3-hydroxy-5-methyoxybenzylalcohol, hericene A, and hericene D from the fruiting bodies, all of which showed potent α-glucosidase inhibition; Miyazawa et al. [15] also reported similar activity for isohericerin and N-dephenylethyl isohericerin. Our previous work isolated multiple isoindolinone monomers and dimers from *H. erinaceus* fermentation and confirmed their cytotoxic and α-glucosidase inhibitory activities [13,16,17]. 

Nevertheless, research on the antihyperglycemic activity of aromatic compounds from this fungus remains limited, with unclear structure–activity relationships, and most existing studies have focused on known compounds, highlighting the need to explore new structural types. To address this, we continued our investigation of the aromatic constituents from *H. erinaceus* and isolated thirteen previously undescribed isoindolinone alkaloids, named erinacenones M–Y (**1**–**13**), from the same culture batch as that referenced in our previous study [13]. Although the compounds in this study were obtained from the same fermentation batch as our previous report [13], they were not co-isolated previously owing to co-elution with known analogs and the complexity of stereochemical determination (requiring semisynthesis and optical rotation comparison). Therefore, we optimized the chromatographic separation and employed combined spectroscopic and chemical derivatization to characterize the structures, aiming to discover novel hypoglycemic lead compounds and to provide a scientific basis for developing functional products derived from this fungus.

## 2. Results and Discussion

### Structural Elucidation of the Previously Undescribed Compounds

**Figure 1:**
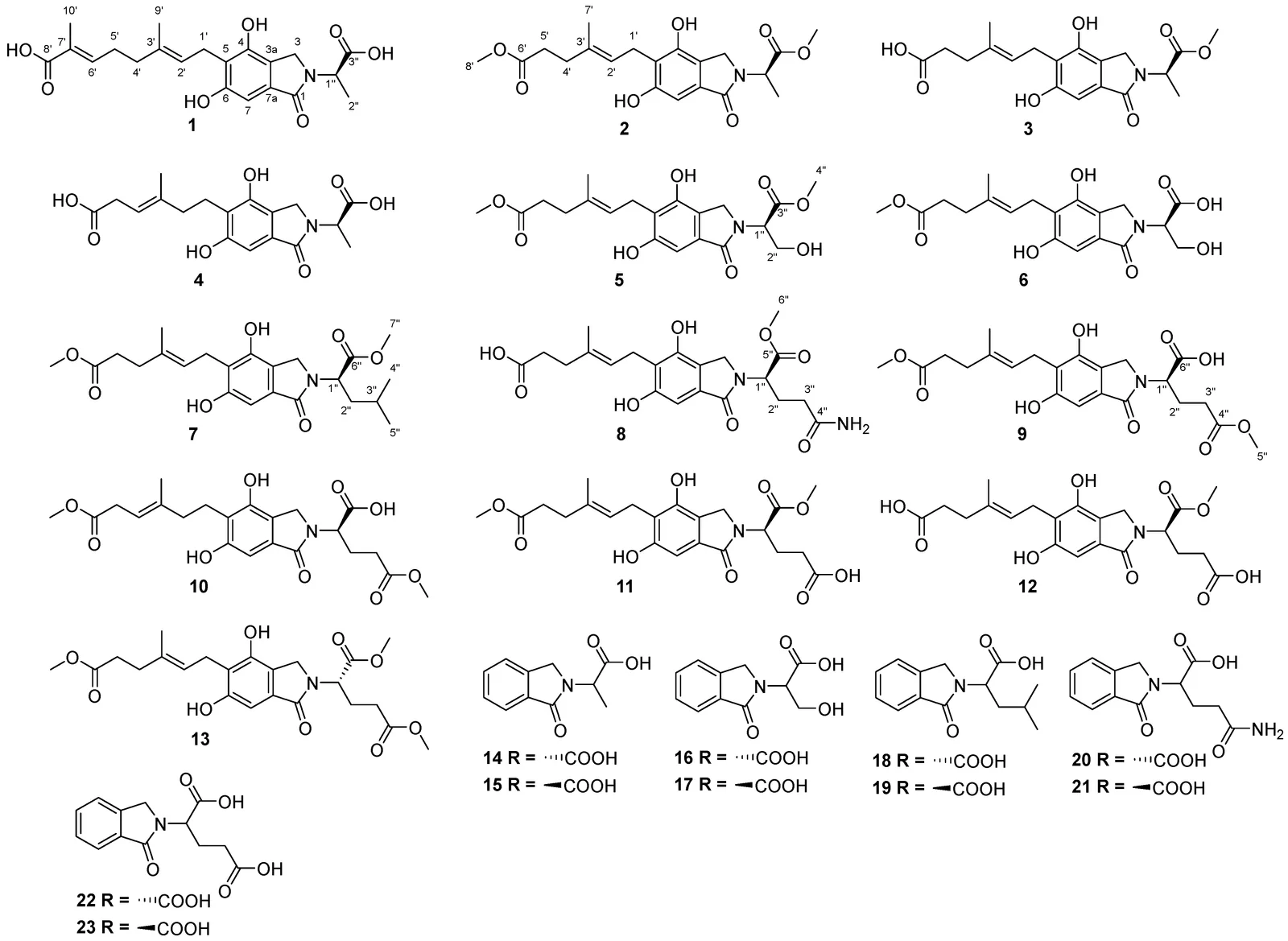
Structures of compounds **1**–**23**.

Compound **1** was obtained as a yellow oil, and the molecular ion peak at *m*/*z* 404.17075 [M+H]^+^ obtained by HRESIMS revealed that it had the molecular formula C_21_H_25_NO_7_. The ^1^H NMR spectrum (Table 1) of **1** showed three methyl groups (*δ*_H_ 1.81, 1.72 and 1.55), three methines, including an aromatic proton (*δ*_H_ 6.75, s), and two trisubstituted olefinic protons (*δ*_H_ 6.67, t, *J* = 7.2 Hz; 5.29, t, *J* = 7.2 Hz). In the ^13^C NMR spectrum (Table 1), 21 carbon signals were observed, including three methyls (*δ*_C_ 16.3, 15.8, and 12.4), four methylenes (*δ*_C_ 46.3, 39.4, 28.2, and 23.6), four methines (one aromatic at *δ*_C_ 101.9; two olefinic at *δ*_C_ 143.4, 124.6), and ten quaternary carbons (three carbonyl at *δ*_C_ 171.6, 173.5, and 172.1; five aromatic at *δ*_C_ 157.9, 151.7, 131.4, 121.8, and 121.1; two olefinic at *δ*_C_ 134.6, 129.2. The ^1^H and ^13^C NMR data for **1** showed that it shared the same substructure of (2*E*,6*E*)-8-(4,6-dihydroxy-1-oxoisoindolin-5-yl)-2,6-dimethylocta-2,6-dienoic acid [18], except for the presence of an additional methyl group (*δ*_H_ 1.55, t, *J* = 7.3 Hz; *δ*_C_ 15.8), a methine (*δ*_H_ 4.98, overlapped; *δ*_C_ 51.8), together with a carboxylic acid (*δ*_C_ 173.5) in **1**. The HMBC correlations from H-1″ to C-1/C-3/C-3″, as well as the COSY correlations between H-1″ and H-2″, established that the 2-substituted propanoic acid moiety was linked to the nitrogen atom. The ROESY correlations of H-2′/H-4′ and H-5′/H-10′ confirmed the *E* configurations for two double bonds. Thus, the structure of **1** was designated as erinacenone M in Figure 1.

Compound **2** was obtained as a yellow oil, and the molecular ion peak at *m*/*z* 392.17026 [M+H]^+^ obtained by HRESIMS revealed that it had the molecular formula C_20_H_25_NO_7_. The ^1^H NMR spectrum (Table 1) of **2** showed four methyl groups, including two oxygenated methyl groups (*δ*_H_ 3.73 and 3.55), an aromatic proton (*δ*_H_ 6.75, s), and a trisubstituted olefinic proton (*δ*_H_ 5.28, t, *J* = 7.2 Hz). In the ^13^C NMR spectrum (Table 1), 20 carbon signals were observed, including four methyls (two oxygenated at *δ*_C_ 52.9, 51.9; one olefinic at *δ*_C_ 16.1; one aliphatic at *δ*_C_ 15.8), four methylenes (*δ*_C_ 46.4, 36.0, 33.9, and 23.6), three methines (one aromatic at *δ*_C_ 101.8; one olefinic at *δ*_C_ 124.5; other at *δ*_C_ 51.2), and nine quaternary carbons (three carbonyl at *δ*_C_ 175.8, 173.5, and 171.6; five aromatic at *δ*_C_ 158.2, 151.7, 131.3, 121.7, and 120.9; one olefinic at *δ*_C_ 134.3). The ^1^H and ^13^C NMR data for **2** showed that it shared the same substructure as (*E*)-methyl 6-(4,6-dihydroxy-1-oxoisoindolin-5-yl)-4-methylhex-4-enoate [18], except for the presence of two additional methyl groups [an oxygenated (*δ*_H_ 3.73, s; *δ*_C_ 52.9), (*δ*_H_ 1.59, d, *J* = 7.4 Hz; *δ*_C_ 15.8)], a methine (*δ*_H_ 4.99, dd, *J* = 14.8, 7.4 Hz; *δ*_C_ 51.2), together with a carboxylic acid (*δ*_C_ 173.5) in **2**. The HMBC correlations from H-1″ to C-1/C-3/C-3″, from H-4″ to C-3″, as well as the COSY correlations between H-1″ and H-2″, established the methyl 2-substituted propanoate moiety was linked to the nitrogen atom. The ROESY correlations of H-1′/H-7′ and H-2′/H-4′ assigned the *E* configuration to the double bond. Thus, the structure of **2** was designated as erinacenone N.

**Figure 2:**
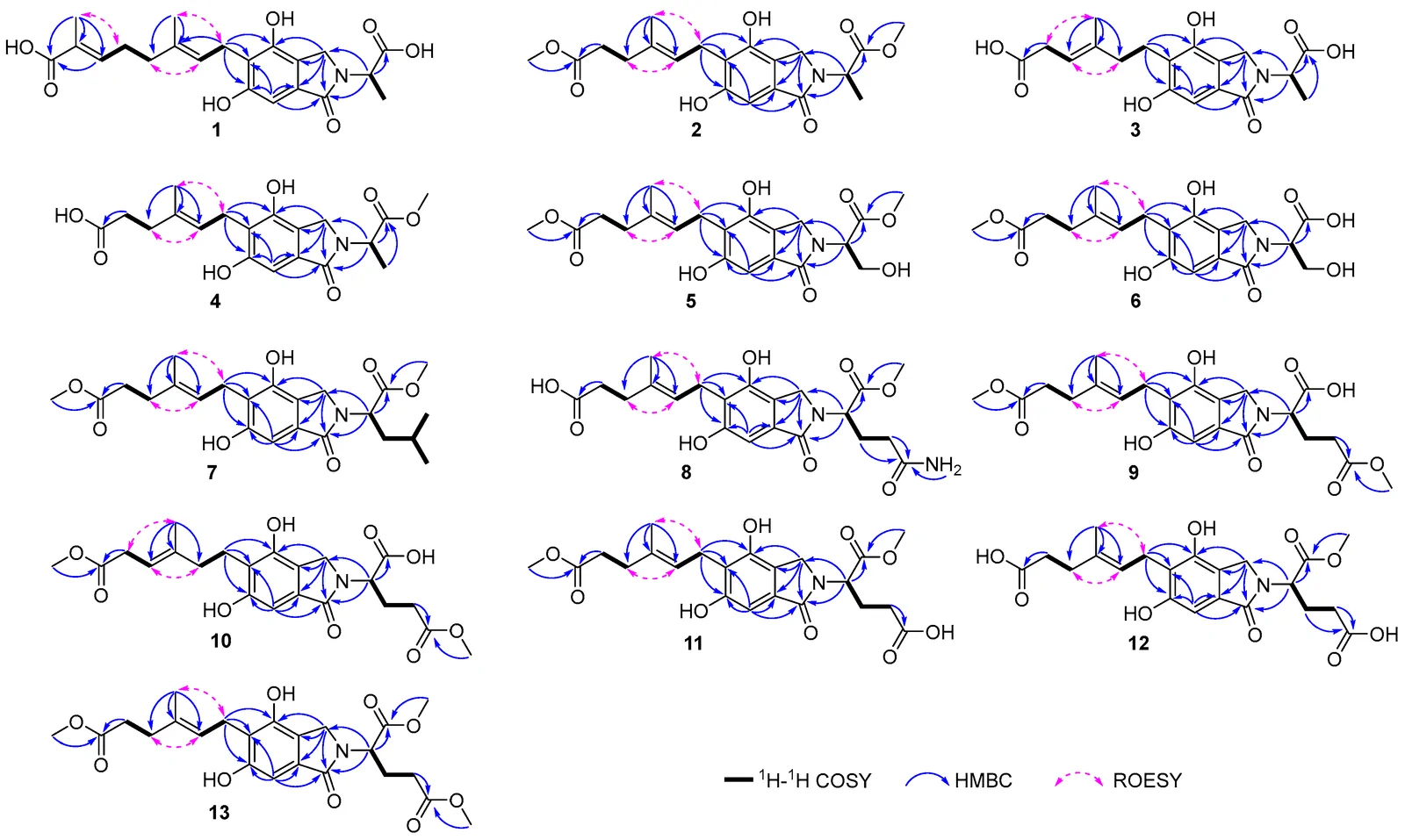
Key 2D NMR correlations for **1**–**13**.

Compounds **3** and **4** were both obtained as yellow oils, with HRESIMS analysis revealing molecular ion peaks at *m*/*z* 400.13681 [M+Na]^+^ (C_19_H_23_NO_7_) and *m*/*z* 386.12094 [M+Na]^+^ (C_18_H_21_NO_7_), respectively. The ^1^H and ^13^C NMR data of **3** and **4** indicated that the presence of (*E*)-methyl 6-(4,6-dihydroxy-1-oxoisoindolin-5-yl)-4-methylhex-4-enoic acid moiety [18] in their structures, including one olefinic methyl (**3**: *δ*_H_ 1.81, s; *δ*_C_ 54.7; 4: *δ*_H_ 1.75, s), four methylenes [**3**: *δ*_H_ 4.37, m; *δ*_C_ 46.4; *δ*_H_ 3.42, d, *J* = 7.2 Hz; *δ*_C_ 23.6; *δ*_H_ 2.27, m; *δ*_C_ 36.8; *δ*_H_ 2.21, m; *δ*_C_ 39.7; **4**: *δ*_H_ 4.50, d, *J* = 16.5 Hz, 4.33, d, *J* = 16.5 Hz; *δ*_C_ 46.4; *δ*_H_ 2.97, t, *J* = 7.5 Hz; *δ*_C_ 35.2; *δ*_H_ 2.83, m; *δ*_C_ 23.3; *δ*_H_ 2.23, m; *δ*_C_ 39.7], two methines [one aromatic (**3**: *δ*_H_ 6.75, s; *δ*_C_ 101.9; 4: *δ*_H_ 6.73, s; *δ*_C_ 101.7); one olefinic (**3**: *δ*_H_ 5.29, t, *J* = 6.9 Hz; *δ*_C_ 123.7; **4**: *δ*_H_ 5.23, t, *J* = 6.8 Hz; *δ*_C_ 117.8)], and eight quaternary carbons [two carbonyl (**3**: *δ*_C_ -, 171.6; 4: *δ*_C_ 177.6, 171.7); five aromatic (**3**: *δ*_C_ 158.2, 151.7, 131.3, 121.9, and 121.0; **4**: *δ*_C_ 158.0, 151.9, 131.8, 122.1, and 121.2); one olefinic (**3**: *δ*_C_ 135.3; **4**: *δ*_C_ 139.8)]. Compounds **3** and **4** contain methyl 2-substituted propanoate and 2-substituted propanoic acid moiety, respectively, as confirmed above by HMBC data analysis and ^1^H-^1^H COSY. The HMBC correlations from H-1″ to C-1/C-3 confirmed the linkage of the methyl 2-substituted propanoate moiety with the nitrogen atom in **3** (Figure 2). ROESY correlations of H-1′/H-7′ and H-2′/H-4′ assigned the *E* configuration to the double bond. Thus, the structure of **3** was designated as erinacenone O. In a similar way, **4** was identified through analysis of its 2D NMR data (Figure 2). Thus, the structure of **4** was designated as erinacenone P in Figure 1.

Compound **5** was obtained as a yellow oil, and its molecular formula was determined to be C_20_H_25_NO_8_ (*m*/*z* 430.14725 [M+Na]^+^, calculated for C_20_H_25_NO_8_Na, 430.14724) through the analysis of HRESIMS. The NMR spectra (Table 2) of **5** were similar to those of erinacerin G [18], except for the presence of an additional oxygenated methyl group (*δ*_H_ 3.75, s; *δ*_C_ 52.9), a methylene (*δ*_H_ 4.14, dd, *J* = 11.8, 7.3 Hz; 4.05, dd, *J* = 11.8, 3.9 Hz; *δ*_C_ 61.6), a methine (*δ*_H_ 5.02, dd, *J* = 7.3, 3.9 Hz; *δ*_C_ 58.2), together with a carboxylic acid (*δ*_C_ 171.2). The ^1^H–^1^H COSY correlation between H-1″ and H-2″, in combination with the HMBC correlations from H-1″ to C-1/C-3/C-3″ and from H-4″ to C-3″, confirmed the linkage of the methyl 2-(hydroxymethyl)-substituted-propanoate moiety to the nitrogen atom. The ROESY correlations of H-1′/H-7′ and H-2′/H-4′ assigned the *E* configuration of the double bond. Consequently, the structure of **5** was named erinacenone Q.

Compound **6** was obtained as a yellow oil, and its molecular formula was determined to be C_19_H_23_NO_8_ (*m*/*z* 394.14969 [M+H]^+^, calculated for C_19_H_24_NO_8_, 394.14964) through the analysis of HRESIMS. The NMR spectra (Table 2) of **6** showed that it was structurally similar to **5**, with the only difference being the absence of a methyl group, which was supported by HRESIMS data. The configuration of the double bond between C-2′ and C-3′ was determined as described for compound **5**. Consequently, the structure of **6** was named erinacenone R.

Compound **7** was obtained as a yellow oil, and its molecular formula was determined to be C_23_H_31_NO_7_ (*m*/*z* 434.21725 [M+H]^+^, calculated for C_23_H_32_NO_7_, 434.21733) through the analysis of HRESIMS. The ^1^H and ^13^C NMR spectra (Table 2) of **7** were similar to those of erinacerin G, except for the presence of three additional methyl groups [an oxygenated (*δ*_H_ 3.72, s; *δ*_C_ 52.9); other (*δ*_H_ 0.98, d, *J* = 6.2 Hz; *δ*_C_ 23.4); (*δ*_H_ 0.96, d, *J* = 6.2 Hz; *δ*_C_ 21.4)], a methylene (*δ*_H_ 1.94, m; 1.83, m; *δ*_C_ 39.4), two methine [(*δ*_H_ 5.05, dd, *J* = 11.4, 4.5 Hz; *δ*_C_ 53.6); (*δ*_H_ 1.46, m; *δ*_C_ 26.1)], together with a carboxylic acid (*δ*_C_ 173.4). The HMBC correlations from H-1″ to C-1/C-3/C-6″, from H-7″ to C-6″, as well as the COSY correlations between H-2″ and H-1″/H-3″, H-3″ and H-4″/H-5″, established that the methyl 2-isopropyl-2-substituted-propanoate moiety was linked to the nitrogen atom. The configuration of the double bond between C-2′ and C-3′ was determined as described for compound **6**. Consequently, the structure of **7** was named erinacenone S.

Compound **8** was obtained as a yellow oil, and its molecular formula was determined to be C_21_H_26_N_2_O_8_ (*m*/*z* 435.17612 [M+H]^+^, calculated for C_21_H_27_N_2_O_8_, 435.17619) through the analysis of HRESIMS. The ^1^H and ^13^C NMR data (Table 2) of **8** were similar to those of erinacerin G, except for the presence of an additional oxygenated methyl group (*δ*_H_ 3.74, s; *δ*_C_ 53.0), two methylenes (*δ*_H_ 2.45, m; 2.21, overlapped; *δ*_C_ 26.4; *δ*_H_ 2.27, m; 2.23, overlapped; *δ*_C_ 32.7), a methine (*δ*_H_ 4.95, dd, *J* = 10.6, 4.8 Hz; *δ*_C_ 55.2), as well as two carboxylic acids (*δ*_C_ 177.2, 172.5). The HMBC correlations from H-1″ to C-1/C-3/C-5″, from H-2″ to C-4″, from H-6″ to C-5″, as well as the COSY correlations between H-2″ and H-1″/H-3″, established that the methyl 5-carbamoyl-2-substituted pentanoate moiety was linked to the nitrogen atom. The configuration of the double bond between C-2′ and C-3′ was determined as described for compound **7**. Consequently, the structure of **8** was named erinacenone T.

Compounds **9** and **10** were isolated as yellow oils. They possessed the same molecular formula of C_22_H_27_NO_9_, with nine degrees of unsaturation, as established by the HRESIMS and NMR data analyses. The NMR data (Table 3 and Table 4) indicated structural similarity to erinacerin G, with both compounds sharing the (*E*)-methyl 6-(4,6-dihydroxy-1-oxoisoindolin-5-yl)-4-methylhex-4-enoate moiety, but differing only in the nitrogen-linked moiety. A comparison of the NMR data of **9** with those obtained for erinacerin G revealed the presence of an additional oxygenated methyl group (*δ*_H_ 3.56, s; *δ*_C_ 52.2), two methylenes (*δ*_H_ 2.46, m; 2.19, m; *δ*_C_ 26.3; *δ*_H_ 2.37, overlapped; *δ*_C_ 31.8), a methine (*δ*_H_ 4.89, overlapped; *δ*_C_ 55.7), as well as two carboxylic acids (*δ*_C_ 174.8, 172.2) in **9**. The HMBC correlations from H-1″ to C-1/C-3/C-6″, from H-3″ to C-4″, from H-6″ to C-4″, as well as the COSY correlations between H-2″ and H-1″/H-3″, established that the methyl 5-(methoxycarbonyl)-2-substituted pentanoic acid moiety was present in the molecule. The double bond configuration between C-2′ and C-3′ was determined as described in compound **8**. As a result, the structure of **9** was elucidated as erinacenone U. In a similar way, the structure of **10** was determined as erinacenone V after a thorough analysis of its HMBC and ROESY spectra (Figure 2). Compounds **9** and **10** are a pair of double-bond positional isomers.

Compound **11** was isolated as a yellow oil. Based on the HRESIMS at *m*/*z* 450.17581 [M+H]^+^, the molecular formula was determined to be C_22_H_27_NO_9_, with nine degrees of unsaturation. The NMR spectra (Table 3 and Table 4) of **11** revealed a structural similarity to **9**, distinguished primarily by the relocation of a methyl group. This change was confirmed by an HMBC correlation from H-6″ to C-5″. ROESY correlations of H-1′/H-7′ and H-2′/H-4′ assigned the *E* configuration to the double bond. As a result, the structure of **11** was named erinacenone W.

Compound **12** was isolated as a yellow oil. Based on the HRESIMS at *m*/*z* 458.14215 [M+Na]^+^, the molecular formula of **12** was determined to be C_21_H_25_NO_9_, with nine degrees of unsaturation. The primary difference between the NMR spectra (Table 3 and Table 4) of **12** and **11** was that the methyl carboxylate of C-6″ was converted into a carboxylic acid. This change was confirmed by HRESIMS data. ROESY correlations of H-1′/H-7′ and H-2′/H-4′ assigned the *E* configuration to the double bond. As a result, the structure of **12** was named erinacenone X.

Compound **13** was isolated as a yellow oil. Based on the HRESIMS at *m*/*z* 464.19147 [M+H]^+^, the molecular formula was determined to be C_23_H_29_NO_9_, with nine degrees of unsaturation. Comparison of the ^1^H and ^13^C NMR spectra (Table 3 and Table 4) of **13** with those of **11** showed that the primary distinction was the presence of an extra methyl group, which was further confirmed by the HMBC correlations from H-5″ to C-4″. ROESY correlations of H-1′/H-7′ and H-2′/H-4′ assigned the *E* configuration to the double bond. As a result, the structure of **13** was named erinacenone Y.

To determine the absolute configurations of compounds **1**–**13**, phthalimidines **14**–**23** were synthesized by the reaction of o-phthalaldehyde with the enantiomerically pure α-amino acids (L/D-alanine, L/D-glutamic acid, L/D-serine, L/D-leucine, and L/D-glutamine). Comparative analysis of the specific optical rotations of compounds **1**–**13** and their corresponding synthetic phthalimidines **14**–**23** revealed that compounds **1**–**12** showed (+)-specific optical rotations, indicating the R configuration at C-1″. In contrast, compound **13** showed (−)-specific optical rotation, confirming an S configuration at C-1″.

## 3. Materials and Methods

### 3.1. General Experimental Procedures

Optical rotations were measured on a JASCO P-2000 Automatic polarimeter (JASCO, Tokyo, Japan). CD spectra were recorded on a JASCO J-1500 CD Spectrometer (JASCO, Tokyo, Japan), and the UV spectra were obtained on an Analytik Jena SPECORD S600 UV spectrophotometer (Analytik Jena, Jena, Germany). NMR spectra were obtained on Bruker Avance 600 MHz and Bruker Avance III 500 spectrometers (Bruker, Ettlingen, Germany) with tetramethylsilane as an internal standard at room temperature. HRESIMS was recorded on a Q Exactive Orbitrap mass spectrometer (Thermo Scientific, Cambridge, MA, USA). Preparative HPLC was performed on an Agilent 1260 liquid chromatography system with DAD detector, using Zorbax SB-C18 column (5 µm, 9.4 × 150 mm) (Agilent, Santa Clara, CA, USA). Preparative HPLC was performed on a Hanbon NP7005C liquid chromatography system, using Zorbax SB-C18 column (5 µm, 9.4 × 150 mm) (Agilent, Santa Clara, CA, USA). On a PuriFlash 450 equipment (Interchim, Montluçon, France), medium pressure liquid chromatography (MPLC) was carried out. Silica gel (80–100, 300–400 mesh) (Qingdao Marine Chemical Factory, Qingdao, China) and Sephadex LH-20 (20–45 μm) (GE Healthcare, Uppsala, Sweden) were used for column chromatography (CC). TLC was carried out using GF254 plates (Qingdao Marine Chemical Factory, Qingdao, China).

### 3.2. Fungal Material

The fungus *H. erinaceus* was collected in Shangri-La County, Yunnan Province, China, in August 2007 and identified by Prof. Zang from Kunming Institute of Botany, Chinese Academy of Sciences. A voucher specimen (No. KU855351.1) has been deposited at South-Central Minzu University, Wuhan. *H. erinaceus* was cultured on slants of potato dextrose agar (20% potato, 2% glucose, 0.3% KH_2_PO_4_, 0.15% MgSO_4_, 0.01% citric acid, pH 6.5) at 25 °C for 7 days. Agar plugs (1 cm^2^) were inoculated in liquid medium (glucose 5%, yeast extract 0.4%, peptone 0.15%, KH_2_PO_4_ 0.05%, MgSO_4_ 0.05%, pH 6.5) and then cultured for 30 days at 25 °C on a rotary shaker at 150 rpm.

### 3.3. Extraction and Isolation

A 100 L culture broth filtrate was concentrated to 10 L. Mycelium was extracted with acetone, and the extract was concentrated under reduced pressure to dryness. The combined extracts were extracted with EtOAc, then they were concentrated under reduced pressure to give a crude extract (110.98 g), which was fractionated by silica gel CC eluting with a gradient of CHCl_3_–MeOH (*v*/*v* 1:0–0:1) to obtain six fractions, A–F. Fraction B was separated by MPLC (MeOH–H_2_O, 5:1, 20:1, 30:1, 45:1, 65:1, 75:1, 85:1, 100:1; 4 L for each step) to offer twelve subfractions (B1–B12). Subfraction B6 was subjected to silica gel CC (*v*/*v* 1:0–0:1) to yield subfractions (B6A–B6P). Subfraction B6L was purified by HPLC (MeCN–H_2_O, 34% isocratic, 4 mL/min) to give compounds **5** (*t*_R_ = 9.93 min, 9.8 mg), **6** (*t*_R_ = 9.92 min, 1.8 mg), **7** (*t*_R_ = 10.13 min, 9.0 mg), and **9** (*t*_R_ = 10.61 min, 2.6 mg). Fraction C was separated by MPLC (MeOH–H_2_O, 5:1, 20:1, 30:1, 45:1, 65:1, 75:1, 85:1, 100:1; 4 L for each step) into twenty subfractions (C1–C20). Subfraction C6 was further separated by silica gel CC (CHCl_3_–MeOH from *v*/*v* 1:0–0:1) to yield ten subfractions (C6A–C6J). Compound **11** (*t*_R_ = 9.90 min, 1.1 mg) was obtained from subfraction C6D by HPLC (MeCN–H_2_O, 32% isocratic, 4 mL/min). Subfraction C10 was separated using silica gel CC (*v*/*v* 1:0–0:1) to get eleven subfractions (C10A–C10K). Compound **4** (*t*_R_ = 9.84 min, 28.3 mg) was purified from subfraction C10A by HPLC (MeCN–H_2_O, 20% isocratic, 4 mL/min). Subfraction C11 was separated by silica gel CC (*v*/*v* 1:0–0:1) to get fifteen subfractions (C11A–C11O). Compound **8** was purified from subfraction C11J by HPLC (MeCN–H_2_O, 28% isocratic, 4 mL/min). Subfraction C11L was subjected to Sephadex LH-20 (MeOH) and further purified by HPLC (MeCN–H_2_O, 25% isocratic, 4 mL/min) to yield compounds **12** (*t*_R_ = 8.69 min, 9.1 mg) and **10** (*t*_R_ = 9.61 min, 1.0 mg). Subfraction C12 was separated by silica gel CC (*v*/*v* 1:0–0:1) to obtain nine subfractions (C12A–C12I). Fraction F was separated by MPLC (MeOH–H_2_O, 5:1, 20:1, 30:1, 45:1, 65:1, 75:1, 85:1, 100:1; 4 L for each step) to offer twenty-three subfractions (F1–F23). Subfraction F8 was subjected to silica gel CC (*v*/*v* 1:0–0:1) to obtain eight subfractions (F8A–F8H). Subfraction F8B was further separated on HPLC (MeCN–H_2_O, 13% isocratic, 4 mL/min) to give compound **13** (*t*_R_ = 6.43 min, 1.1 mg). Subfraction F10 was separated using silica gel CC (*v*/*v* 1:0–0:1) to get subfractions (F10A–F10L). Compounds **2** (*t*_R_ = 5.45 min, 16.2 mg) and **3** (*t*_R_ = 6.28 min, 5.2 mg) were purified from subfraction F10E by HPLC (MeCN–H_2_O, 10% isocratic, 4 mL/min). Compound **1** (*t*_R_ = 7.58 min, 17.3 mg) was obtained from subfraction F10G by HPLC (MeCN–H_2_O, 20% isocratic, 4 mL/min).

### 3.4. Characterization Data

#### 3.4.1. Erinacenone M (**1**)

yellow oil; [α]^25^_D_ +55.5 (*c* 0.02, MeOH); UV (MeOH) *λ*_max_ (log *ε*) 215 (4.62), 260 (4.11), 300 (3.47) nm; ^1^H (600 MHz) and ^13^C NMR (150 MHz) data (CD_3_OD), see Table 1; HRESIMS *m*/*z* 404.17075 (calcd for C_21_H_26_NO_7_ [M+H]^+^, 404.17038).

#### 3.4.2. Erinacenone N (**2**)

yellow oil; [α]^25^_D_ +37.7 (*c* 0.03, MeOH); UV (MeOH) *λ*_max_ (log *ε*) 220 (4.51), 260 (4.22), 300 (3.62) nm; ^1^H (500 MHz) and ^13^C NMR (125 MHz) data (CD_3_OD), see Table 1; HRESIMS *m*/*z* 392.17026 (calcd for C_20_H_26_NO_7_ [M+H]^+^, 392.17038).

#### 3.4.3. Erinacenone O (**3**)

yellow oil; [α]^25^_D_ +63.1 (*c* 0.02, MeOH); UV (MeOH) *λ*_max_ (log *ε*) 215 (4.50), 260 (4.03), 300 (3.46) nm; ^1^H (600 MHz) and ^13^C NMR (150 MHz) data (CD_3_OD), see Table 1; HRESIMS *m*/*z* 400.13681 (calcd for C_19_H_23_NO_7_ [M+Na]^+^, 400.13667).

#### 3.4.4. Erinacenone P (**4**)

yellow oil; [α]^25^_D_ +48.2 (*c* 0.03, MeOH); UV (MeOH) *λ*_max_ (log *ε*) 220 (4.46), 260 (4.19), 300 (3.60) nm; ^1^H (600 MHz) and ^13^C NMR (150 MHz) data (CD_3_OD), see Table 1; HRESIMS *m*/*z* 386.12094 (calcd for C_18_H_21_NO_7_Na [M+Na]^+^, 386.12102).

#### 3.4.5. Erinacenone Q (**5**)

yellow oil; [α]^25^_D_ +23.1 (*c* 0.03, MeOH); UV (MeOH) *λ*_max_ (log *ε*) 215 (4.44), 260 (4.03), 300 (3.42) nm; ^1^H (500 MHz) and ^13^C NMR (125 MHz) data (CD_3_OD), see Table 2; HRESIMS *m*/*z* 430.14725 (calcd for C_20_H_25_NO_8_Na [M+Na]^+^, 430.14724).

#### 3.4.6. Erinacenone R (**6**)

yellow oil; [α]^25^_D_ +37.5 (*c* 0.02, MeOH); UV (MeOH) *λ*_max_ (log *ε*) 215 (4.53), 260 (4.13), 300 (3.52) nm; ^1^H (500 MHz) and ^13^C NMR (125 MHz) data (CD_3_OD), see Table 2; HRESIMS *m*/*z* 394.14969 (calcd for C_19_H_24_NO_8_ [M+H]^+^, 394.14964).

#### 3.4.7. Erinacenone S (**7**)

yellow oil; [α]^25^_D_ +12.5 (*c* 0.03, MeOH); UV (MeOH) *λ*_max_ (log *ε*) 220 (4.47), 260 (4.26), 300 (3.66) nm; ^1^H (600 MHz) and ^13^C NMR (150 MHz) data (CD_3_OD), see Table 2; HRESIMS *m*/*z* 434.21725 (calcd for C_23_H_32_NO_7_ [M+H]^+^, 434.21733).

#### 3.4.8. Erinacenone T (**8**)

yellow oil; [α]^25^_D_+11.5 (*c* 0.03, MeOH); UV (MeOH) *λ*_max_ (log *ε*) 210 (4.50), 260 (4.23), 300 (3.67) nm; ^1^H (600 MHz) and ^13^C NMR (150 MHz) data (CD_3_OD), see Table 2; HRESIMS *m*/*z* 435.17612 (calcd for C_21_H_27_N_2_O_8_ [M+H]^+^, 435.17619).

#### 3.4.9. Erinacenone U (**9**)

yellow oil; [α]^25^_D_ +6.6 (*c* 0.03, MeOH); UV (MeOH) *λ*_max_ (log *ε*) 215 (4.47), 260 (4.13), 300 (3.50) nm; ^1^H (500 MHz) and ^13^C NMR (125 MHz) data (CD_3_OD), see Table 3 and Table 4; HRESIMS *m*/*z* 450.17572 (calcd for C_22_H_28_NO_9_ [M+H]^+^, 450.17586).

#### 3.4.10. Erinacenone V (**10**)

yellow oil; [α]^25^_D_ +7.7 (*c* 0.02, MeOH); UV (MeOH) *λ*_max_ (log *ε*) 215 (4.52), 260 (4.10), 300 (3.48) nm; ^1^H (500 MHz) and ^13^C NMR (125 MHz) data (CD_3_OD), see Table 3 and Table 4; HRESIMS *m*/*z* 450.17578 (calcd for C_22_H_28_NO_9_ [M+H]^+^, 450.17586).

#### 3.4.11. Erinacenone W (**11**)

yellow oil; [α]^25^_D_ +10.0 (*c* 0.03, MeOH); UV (MeOH) *λ*_max_ (log *ε*) 215 (4.55), 260 (4.22), 300 (3.63) nm; ^1^H (600 MHz) and ^13^C NMR (150 MHz) data (CD_3_OD), see Table 3 and Table 4; HRESIMS *m*/*z* 450.17581 (calcd for C_22_H_28_NO_9_ [M+H]^+^, 450.17586).

#### 3.4.12. Erinacenone X (**12**)

yellow oil; [α]^25^_D_ +14.3 (*c* 0.02, MeOH); UV (MeOH) *λ*_max_ (log *ε*) 215 (4.51), 260 (4.09), 300 (3.47) nm; ^1^H (600 MHz) and ^13^C NMR (150 MHz) data (CD_3_OD), see Table 3 and Table 4; HRESIMS *m*/*z* 458.14215 (calcd for C_21_H_25_NO_9_Na [M+Na]^+^, 458.14215).

#### 3.4.13. Erinacenone Y (**13**)

yellow oil; [α]^25^_D_ −19.0 (*c* 0.05, MeOH); UV (MeOH) *λ*_max_ (log *ε*) 225 (4.48), 260 (4.33), 300 (3.81) nm; ^1^H (600 MHz) and ^13^C NMR (150 MHz) data (CD_3_OD), see Table 3 and Table 4; HRESIMS *m*/*z* 464.19147 (calcd for C_23_H_30_NO_9_ [M+H]^+^, 464.19151).

Synthesis of Phthalimidines (**14**–**23**). L-Alanine (0.5 mmol) was added to a solution of phathalic dicarboxaldehyde (0.5 mmol) in 10 mL of acetonitrile. The mixture was heated under relux for 16 h; then the reaction mixture was filtered while hot. The solvent was allowed to cool to yield the desired phathalimidine 14 as crystalline solids (92 mg). Using the same method, compounds **15** (71 mg), **16** (8 mg), **17** (53 mg), **18** (98 mg), **19** (55 mg), **20** (112 mg), **21** (82 mg), **22** (65 mg), and **23** (48 mg) were synthesized from D-alanine, L-glutamic acid, D-glutamic acid, L-serine, D-serine, L-leucine, D-leucine, L-glutamine, and D-glutamine, respectively.

Compound **14**: colorless microcrystalline powder; [α]^25^_D_ +16.537 (*c* 0.08, MeOH); ^1^H NMR (CD_3_OD, 600 MHz): 7.79 (1H, d, *J* = 7.6 Hz, H-7), 7.63 (1H, td, *J* = 7.6, 1.0 Hz, H-5), 7.60 (1H, d, *J* = 7.5 Hz, H-4), 7.51 (1H, t, *J* = 7.4 Hz, H-6), 5.02 (1H, dd, *J* = 15.0, 7.5 Hz, H-1′), 4.62 (1H, d, *J* = 17.2 Hz, H-3), 4.57 (1H, d, *J* = 17.2 Hz, H-3), 1.63 (3H, d, *J* = 7.5 Hz, H-2′); ^13^C NMR (CD_3_OD, 150 MHz): 174.7 (C-3′), 171.0 (C-1), 143.8 (C-3a), 133.2 (C-5), 133.0 (C-7a), 129.2 (C-6), 124.4 (C-4), 124.3 (C-7), 51.0 (C-1′), 48.6 (C-3), 15.9 (C-2′); HRESIMS *m*/*z* 206.08122 (calcd for C_11_H_12_NO_3_ [M+H]^+^, 206.08117).

Compound **15**: colorless microcrystalline powder; [α]^25^_D_ −26.527 (*c* 0.08, MeOH); ^1^H (600 MHz) and ^13^C NMR (150 MHz) data of **15** are identical with those of **14**; HRESIMS *m*/*z* 206.08127 (calcd for C_11_H_12_NO_3_ [M+H]^+^, 206.08117).

Compound **16**: colorless microcrystalline powder; [α]^25^_D_ +43.206 (c 0.09, MeOH); ^1^H NMR (CD_3_OD, 600 MHz): 7.80 (1H, d, *J* = 7.6 Hz, H-7), 7.63 (1H, td, *J* = 7.6, 1.0 Hz, H-5), 7.60 (1H, d, *J* = 7.5 Hz, H-4), 7.51 (1H, t, *J* = 7.5 Hz, H-6), 5.06 (1H, dd, *J* = 7.6, 3.8 Hz, H-1′), 4.74 (1H, d, *J* = 17.4 Hz, H-3), 4.68 (1H, d, *J* = 17.4 Hz, H-3), 4.17 (1H, dd, *J* = 11.8, 7.6 Hz, H-2′), 4.10 (1H, dd, *J* = 11.8, 3.8 Hz, H-2′); ^13^C NMR (CD_3_OD, 150 MHz): 172.2 (C-3′), 171.7 (C-1), 144.2 (C-3a), 133.2 (C-5), 132.8 (C-7a), 129.1 (C-6), 124.3 (C-4, 7), 61.5 (C-2′), 58.1 (C-1′), 49.9 (C-3); HRESIMS *m*/*z* 222.07600 (calcd for C_11_H_12_NO_4_ [M+H]^+^, 222.07608).

Compound **17**: colorless microcrystalline powder; [α]^25^_D_ −53.101 (*c* 0.08, MeOH); ^1^H (600 MHz) and ^13^C NMR (150 MHz) data of **17** are identical with those of **16**; HRESIMS *m*/*z* 222.07605 (calcd for C_11_H_12_NO_4_ [M+H]^+^, 222.07608).

Compound **18**: colorless microcrystalline powder; [α]^25^_D_ −3.731 (*c* 0.13, MeOH); ^1^H NMR (CD_3_OD, 600 MHz): 7.79 (1H, d, *J* = 7.6 Hz, H-7), 7.64 (1H, td, *J* = 7.6, 1.0 Hz, H-5), 7.61 (1H, d, *J* = 7.5 Hz, H-4), 7.52 (1H, t, *J* = 7.5 Hz, H-6), 5.07 (1H, dd, *J* = 11.6, 4.3 Hz, H-1′), 4.68 (1H, d, *J* = 17.2 Hz, H-3), 4.48 (1H, d, *J* = 17.2 Hz, H-3), 1.99 (1H, m, H-2′); 1.88 (1H, m, H-2′); 1.48 (1H, m, H-3′); 0.99 (6H, d, *J* = 6.6 Hz, H-4′, 5′); ^13^C NMR (CD_3_OD, 150 MHz): 174.6 (C-6′), 171.5 (C-1), 143.8 (C-3a), 133.2 (C-5), 132.8 (C-7a), 129.2 (C-6), 124.4 (C-4, 7), 53.5 (C-1′), 48.1 (C-3), 39.4 (C-2′), 26.3 (C-3′), 23.5 (C-5′), 21.3 (C-4′); HRESIMS *m*/*z* 248.12804 (calcd for C_14_H_18_NO_3_ [M+H]^+^, 248.12812).

Compound **19**: colorless microcrystalline powder; [α]^25^_D_ +6.02 (*c* 0.03, MeOH); ^1^H (600 MHz) and ^13^C NMR (150 MHz) data of **19** are identical with those of **18**; HRESIMS *m*/*z* 248.12801 (calcd for C_14_H_18_NO_3_ [M+H]^+^, 248.12812).

Compound **20**: colorless microcrystalline powder; [α]^25^_D_ −11.588 (c 0.11, MeOH); ^1^H NMR (CD_3_OD, 600 MHz): 7.79 (1H, d, *J* = 7.6 Hz, H-7), 7.64 (1H, td, *J* = 7.6, 1.0 Hz, H-5), 7.60 (1H, d, *J* = 7.5 Hz, H-4), 7.51 (1H, t, *J* = 7.5 Hz, H-6), 4.97 (1H, dd, *J* = 10.8, 4.5 Hz, H-1′), 4.63 (1H, d, *J* = 17.1 Hz, H-3), 4.56 (1H, d, *J* = 17.1 Hz, H-3), 2.49 (1H, m, H-2′), 2.30 (1H, m, H-3′), 2.25 (1H, m, H-3′), 2.23 (1H, m, H-2′); ^13^C NMR (CD_3_OD, 150 MHz): 177.3 (C-4′), 173.5 (C-5′), 171.7 (C-1), 143.9 (C-3a), 133.3 (C-5), 132.7 (C-7a), 129.2 (C-6), 124.4 (C-4, 7), 55.1 (C-1′), 48.6 (C-3), 33.1 (C-3′), 26.5 (C-2′); HRESIMS *m*/*z* 263.10266 (calcd for C_13_H_15_N_2_O_4_ [M+H]^+^, 263.10263).

Compound **21**: colorless microcrystalline powder; [α]^25^_D_ +6.822 (*c* 0.03, MeOH); ^1^H (600 MHz) and ^13^C NMR (150 MHz) data of **21** are identical with those of **20**; HRESIMS *m*/*z* 263.10263 (calcd for C_13_H_15_N_2_O_4_ [M+H]^+^, 263.10263).

Compound **22**: colorless microcrystalline powder; [α]^25^_D_ −29.073 (*c* 0.06, MeOH); ^1^H NMR (CD_3_OD, 600 MHz): 7.80 (1H, d, *J* = 7.6 Hz, H-7), 7.64 (1H, td, *J* = 7.6, 1.0 Hz, H-5), 7.60 (1H, d, *J* = 7.5 Hz, H-4), 7.52 (1H, t, *J* = 7.5 Hz, H-6), 5.02 (1H, dd, *J* = 11.0, 4.7 Hz, H-1′), 4.62 (1H, d, *J* = 17.1 Hz, H-3), 4.54 (1H, d, *J* = 17.1 Hz, H-3), 2.48 (1H, m, H-2′), 2.37 (2H, m, H-3′), 2.23 (1H, m, H-2′); ^13^C NMR (CD_3_OD, 150 MHz): 176.0 (C-4′), 173.5 (C-5′), 171.7 (C-1) 143.8 (C-3a), 133.3 (C-5), 132.7 (C-7a), 129.2 (C-6), 124.4 (C-4, 7), 55.0 (C-1′), 48.7 (C-3), 31.6 (C-3′), 25.9 (C-2′); HRESIMS *m*/*z* 264.08667 (calcd for C_13_H_14_NO_5_ [M+H]^+^, 264.08665).

Compound **23**: colorless microcrystalline powder; [α]^25^_D_ +23.427 (*c* 0.09, MeOH); ^1^H (600 MHz) and ^13^C NMR (150 MHz) data of **23** are identical with those of **22**; HRESIMS *m*/*z* 264.08667 (calcd for C_13_H_14_NO_5_ [M+H]^+^, 264.08665).

### 3.5. α-Glucosidase Inhibitory Assay

The inhibitory effect on α-glucosidase was evaluated using a modified protocol based on earlier reports [19,20]. Briefly, each well of a 96-well plate received 125 µL of phosphate buffer (50 mM, pH 6.8), 25 µL of 10 mM p-nitrophenyl-α-D-glucopyranoside, and 25 µL of the test compound solution, yielding final concentrations of 1.25, 2.5, 5, 10, 12.5, 25, and 50 µM. After a 10 min pre-incubation at 37 °C, the enzymatic reaction was initiated by adding 25 µL of α-glucosidase (0.25 U/mL, from *Saccharomyces cerevisiae*). The plate was then incubated at 37 °C for 30 min, and the reaction was terminated by the addition of 50 µL of 1 M Na_2_CO_3_. The absorbance of the liberated p-nitrophenol was read at 405 nm using a microplate reader. Acarbose was included as the positive control, and a blank sample (without enzyme) was prepared by substituting the enzyme with an equal volume of buffer. The inhibition percentage was calculated as: Inhibition (%) = [(ODcontrol − ODcontrol blank) − (ODtest − ODtest blank)]/(ODcontrol − ODcontrol blank) × 100%. All experiments were performed in triplicate, and the data are presented as mean ± SD. 

## 4. Conclusions

This study utilized the same batch of fermented material from *H. erinaceus* (preliminary results reported in our previous study [13]) to isolate and identify thirteen new isoindolin-1-one alkaloids, designated as erinacenones M–Y (**1**–**13**), further enriching the structural diversity of isoindolinone skeletons from this edible fungus. The α-glucosidase inhibitory activities of compounds **1**–**13** were evaluated at concentrations ranging from 1.25 to 50 µM. The inhibition percentages at 50 µM are summarized in Table 5. Among them, compound **11** was identified as a modest α-glucosidase inhibitor, warranting further structural optimization and more comprehensive biological evaluations. This work provides new chemical and pharmacological evidence for the development of functional products derived from *H. erinaceus*. However, the current study remains limited to enzyme-level assays, with unclear mechanisms and binding modes, and the insufficient yields of certain minor components preclude in-depth evaluation. To address these bottlenecks, subsequent research will focus on large-scale fermentation to produce sufficient quantities of active compounds for in vivo efficacy validation, combined with molecular docking and molecular dynamics simulations to elucidate the inhibition mechanism, alongside targeted isolation of trace constituents. Ultimately, these efforts aim to establish a foundation for the development of *H. erinaceus*-based health foods or novel antidiabetic drugs.

## Figures and Tables

**Table 1:** ^1^H NMR and ^13^C NMR spectroscopic data for compounds **1**–**4**.

No.	1		2		3		4	
1	171.6		171.6		171.6		171.7	
3	46.3	4.50, d (16.7) 4.37, d (16.7)	46.4	4.37, d (1.3)	46.4	4.37, m	46.4	4.50, d (16.5) 4.33, d (16.5)
3a	131.4		131.3		131.3		131.8	
4	151.7		151.7		151.7		151.9	
5	121.8		121.7		121.9		122.1	
6	157.9		158.2		158.2		158.0	
7	101.9	6.75, s	101.8	6.75, s	101.9	6.75, s	101.7	6.73, s
7a	121.1		120.9		121.0		121.2	
1′	23.6	3.41, m	23.6	3.41, d (7.2)	23.6	3.42, d (7.2)	23.3	2.83, m
2′	124.6	5.29, t (7.2)	124.5	5.28, t (7.2)	123.7	5.29, t (6.9)	39.7	2.23, m
3′	134.6		134.3		135.3		139.8	
4′	39.4	2.09, t (7.2)	36.0	2.26, t (7.6)	36.8	2.27, m	117.8	5.23, t (6.8)
5′	28.2	2.27, dd (14.6, 7.2)	33.9	2.39, m	39.7	2.21, m	35.2	2.97, t (7.5)
6′	143.4	6.67, t (7.2)	175.8		overlapped		177.6	
7′	129.2		16.1	1.80, s	16.3	1.81, s	16.4	1.57, s
8′	172.1		51.9	3.55, s				
9′	16.3	1.81, s						
10′	12.4	1.72, s						
1″	51.8	4.98, overlapped	51.2	4.99, dd (14.8, 7.4)	51.2	4.99, dd (14.8, 7.4)	52.2	4.89, overlapped
2″	15.8	1.55, d (7.3)	15.8	1.59, d (7.4)	15.8	1.59, d (7.4)	16.5	1.57, d (7.4)
3″	173.5		173.5		173.6		177.2	
4″			52.9	3.73, s	52.9	3.73, s		

Note: All spectra were measured in methanol-*d*_4_. The NMR spectra of compound **2** were recorded at 500 MHz (^1^H) and 125 MHz (^13^C), whereas compounds **1**, **3** and **4** were measured at 600 MHz (^1^H) and 150 MHz (^13^C).

**Table 2:** ^1^H NMR and ^13^C NMR spectroscopic data for compounds **5**–**8**.

No.	5		6		7		8	
1	172.2		172.3		172.0		172.2	
3	47.8	4.51, d (5.5)	47.6	4.58, d (16.7) 4.44, d (16.7)	46.2	4.43, d (16.5) 4.26, d (16.5)	46.5	4.38, d (16.4) 4.33, d (16.4)
3a	131.1		131.8		131.1		131.0	
4	151.7		151.6		151.7		151.8	
5	121.8		121.4		121.8		122.0	
6	158.1		157.9		158.2		158.2	
7	101.8	6.76, s	101.8	6.76, s	102.0	6.76, s	102.0	6.75, s
7a	121.5		121.3		120.9		121.0	
1′	23.6	3.42, d (7.1)	23.6	3.41, d (7.0)	23.6	3.41, d (7.2)	23.6	3.42, d (6.9)
2′	124.6	5.29, t (6.8)	124.7	5.28, t (7.0)	124.5	5.29, t (7.2)	123.9	5.30, t (7.2)
3′	134.3		134.1		134.3		135.0	
4′	36.0	2.26, t (7.5)	36.0	2.26, t (7.6)	36.0	2.26, t (7.6)	36.4	2.26, overlapped
5′	33.9	2.40, m	33.9	2.39, (m)	33.9	2.39, m	35.1	2.32, m
6′	175.8		175.8		175.8		179.2	
7′	16.1	1.80, s	16.1	1.80, s	16.1	1.80, s	16.3	1.81, s
8′	51.9	3.55, s	52.0	3.56, s	51.9	3.55, s		
1″	58.2	5.02, dd (7.3, 3.9)	59.5	4.90, overlapped	53.6	5.05, dd (11.4, 4.5)	55.2	4.95, dd (10.6, 4.8)
2″	61.6	4.14, dd (11.8, 7.3) 4.05, dd (11.8, 3.9)	62.1	4.08, m	39.4	1.94, m 1.83, m	26.4	2.45, m 2.21, overlapped
3″	171.2		174.8		26.1	1.46, m	32.7	2.27, m 2.23, overlapped
4″	52.9	3.75, s			21.4	0.96, d (6.2)	177.2	
5″					23.4	0.98, d (6.2)	172.5	
6″					173.4		53.0	3.74, s
7″					52.9	3.72, s		

Note: All spectra were measured in methanol-*d*_4_. The NMR spectra of compound **5**, **6** were recorded at 500 MHz (^1^H) and 125 MHz (^13^C), whereas compounds **7**, **8** were measured at 600 MHz (^1^H) and 150 MHz (^13^C).

**Table 3:** ^13^C NMR spectroscopic data for compounds **9**–**13**.

No.	9	10	11	12	13
1	172.2	172.2	172.2	172.2	172.1
3	46.4	46.3	46.6	46.4	46.5
3a	131.3	131.3	131.1	131.5	130.9
4	151.7	151.8	151.7	151.7	151.7
5	121.6	122.2	121.8	121.6	121.9
6	158.1	158.2	158.2	158.0	158.2
7	101.9	101.8	101.9	101.9	101.9
7a	120.9	120.9	120.9	121.0	120.9
1′	23.6	23.4	23.6	23.6	23.6
2′	124.6	39.6	124.5	124.2	124.5
3′	134.2	140.7	134.3	134.5	134.3
4′	36.0	116.8	36.0	36.0	36.0
5′	33.9	34.3	33.9	34.2	33.9
6′	175.8	174.8	175.8	177.9	175.8
7′	16.1	16.4	16.1	16.2	16.1
8′	51.9	52.1	51.9		51.9
1″	55.7	55.8	55.2	56.3	55.0
2″	26.3	26.3	26.5	26.5	25.8
3″	31.8	31.8	32.8	31.9	31.3
4″	174.8	174.7	177.7	174.9	174.6
5″	52.2	52.2	172.7	174.9	52.2
6″	172.2	174.7	52.9	52.1	172.4
7″					53.0

Note: All spectra were measured in methanol-*d*_4_. The ^13^C NMR spectra of compound **9**, **10** were recorded at 125 MHz, whereas compounds **11**, **12** and **13** were measured at 150 MHz.

**Table 4:** ^1^H NMR spectroscopic data for compounds **9**–**13**.

No.	9	10	11	12	13
3	4.44, d (16.4) 4.28, d (16.4)	4.44, d (16.4) 4.27, d (16.4)	4.39, d (16.4) 4.33, d (16.4)	4.46, d (16.5) 4.26, d (16.5)	4.36, d (16.4) 4.29, d (16.4)
7	6.75, s	6.74, s	6.75, s	6.75, s	6.75, s
1′	3.41, d (7.0)	2.82, m	3.41, d (7.1)	3.42, d (6.9)	3.41, d (7.1)
2′	5.28, t (7.0)	2.24, t (8.1)	5.28, t (7.2)	5.30, t (7.1)	5.28, t (7.1)
4′	2.26, t (7.6)	5.25, t (7.1)	2.26, overlapped	2.25, m	2.26, t (7.5)
5′	2.39, overlapped	3.03, d (7.2)	2.39, m	2.34, overlapped	2.39, overlapped
7′	1.80, s	1.74, s	1.80, s	1.81, s	1.80, s
8′	3.55, s	3.55, s	3.55, s		3.55, s
1″	4.89, overlapped	4.89, overlapped	4.98, dd (10.6, 4.8)	4.85, overlapped	4.98, dd (10.8, 4.6)
2″	2.46, m 2.19, m	2.45, m 2.19, m	2.43, m 2.19, m	2.47, m 2.17, m	2.44, m 2.22, m
3″	2.37, overlapped	2.37, m	2.28, overlapped	2.36, overlapped	2.38, overlapped
5″	3.56, s	3.63, s			3.57, s
6″			3.73, s	3.56, s	
7″					3.73, s

Note: Not detected. All spectra were measured in methanol-*d*_4_. The ^1^H NMR spectra of compound **9**, **10** were recorded at 500 MHz, whereas compounds **11**, **12** and **13** were measured at 600 MHz.

**Table 5:** α-glucosidase inhibitory activity of compounds.

Compound	Inhibition (%) at 50 µM	Compound	Inhibition (%) at 50 µM
**1**	8.3 ± 1.2	**8**	5.2 ± 1.5
**2**	6.3 ± 0.8	**9**	9.8 ± 1.7
**3**	5.6 ± 1.0	**10**	7.6 ± 1.4
**4**	4.1 ± 1.2	**11**	31.5 ± 1.2
**5**	8.8 ± 1.5	**12**	3.3 ± 2.1
**6**	10.7 ± 2.0	**13**	13.5 ± 0.7
**7**	12.2 ± 1.3	Acarbose ^a^	5 ± 1.1

^a^ Positive control.

## Data Availability

The original contributions presented in this study are included in the article/Supplementary Materials. Further inquiries can be directed to the corresponding author.

## Supplementary Materials

The following supporting information can be downloaded at: https://www.mdpi.com/article/10.3390/molecules31183333/s1, Figures S1–S91: HRESIMS, HNMR, CNMR, COSY, HSQC, HMBC, ROESY of compounds **1**–**13**. Figures S92–S111: HRESIMS, HNMR, CNMR of compounds **14**–**23** (synthesis of phthalimidines). Table S1: Cytotoxicity of compounds (in IC_50_, μM).
