# Supplementary material for: Erinacenones M–Y, Part 2: Thirteen New Isoindolinone Alkaloids with α-Glucosidase Inhibitory Activity from Liquid Cultures of the Edible and Medicinal Mushroom Hericium erinaceus
Source: Molecules. 2026 Sep 20;31(18):3333. doi: 10.3390/molecules31183333 (PMC13610404; doi:10.3390/molecules31183333)
Supplement: Supplementary file 1 [file molecules-31-03333-s001.zip › molecules-4523648-supplementary.pdf]

SUPPLEMENTARY MATERIAL

Erinacenones M–Y, Part 2: Thirteen New Isoindolinone Alkaloids with α-Glucosidase Inhibitory Activity from Liquid Cultures of the Edible and Medicinal Mushroom *Hericium erinaceus*

Lin-Lin Yuan [1], Ji-Kai Liu [2]*

[1]*College of Food Science and Engineering, Tarim University, Alar 843300, China*

[2]*Anhui Province Key Laboratory of Bioactive Natural Products, School of Pharmacy, Anhui University of Chinese Medicine, Hefei 230012, China*

Emails: yuanlinlin9281@163.com (L.-L. Yuan)

Contents

Figure S1. HRESIMS spectroscopic data of erinacenone M. 4
Figure S2. ^{1}H NMR (600 MHz) spectrum of erinacenone M. 5
Figure S3. ^{13}C NMR and DEPT (150 MHz) spectra of erinacenone M. 5
Figure S4. ^{1}H-^{1}H COSY spectrum of erinacenone M. 6
Figure S5. HSQC spectrum of erinacenone M. 6
Figure S6. HMBC spectrum of erinacenone M. 7
Figure S7. ROESY spectrum of erinacenone M. 7
Figure S8. HRESIMS spectroscopic data of erinacenone N. 8
Figure S9. ^{1}H NMR (500 MHz) spectrum of erinacenone N. 9
Figure S10. ^{13}C NMR and DEPT (125 MHz) spectra of erinacenone N. 9
Figure S11. ^{1}H-^{1}H COSY spectrum of erinacenone N. 10
Figure S12. HSQC spectrum of erinacenone N. 10
Figure S13. HMBC spectrum of erinacenone N. 11
Figure S14. ROESY spectrum of erinacenone N. 11
Figure S15. HRESIMS spectroscopic data of erinacenone O. 12
Figure S16. ^{1}H NMR (600 MHz) spectrum of erinacenone O. 13
Figure S17. ^{13}C NMR and DEPT (150 MHz) spectra of erinacenone O. 13
Figure S18. ^{1}H-^{1}H COSY spectrum of erinacenone O. 14
Figure S19. HSQC spectrum of erinacenone O. 14
Figure S20. HMBC spectrum of erinacenone O. 15
Figure S21. ROESY spectrum of erinacenone O. 15
Figure S22. HRESIMS spectroscopic data of erinacenone P. 16
Figure S23. ^{1}H NMR (600 MHz) spectrum of erinacenone P. 17
Figure S24. ^{13}C NMR and DEPT (150 MHz) spectra of erinacenone P. 17
Figure S25. ^{1}H-^{1}H COSY spectrum of erinacenone P. 18
Figure S26. HSQC spectrum of erinacenone P. 18
Figure S27. HMBC spectrum of erinacenone P. 19
Figure S28. ROESY spectrum of erinacenone P. 19
Figure S29. HRESIMS spectroscopic data of erinacenone Q. 20
Figure S30. ^{1}H NMR (500 MHz) spectrum of erinacenone Q. 21
Figure S31. ^{13}C NMR and DEPT (125 MHz) spectra of erinacenone Q. 21
Figure S32. ^{1}H-^{1}H COSY spectrum of erinacenone Q. 22
Figure S33. HSQC spectrum of erinacenone Q. 22
Figure S34. HMBC spectrum of erinacenone Q. 23
Figure S35. ROESY spectrum of erinacenone Q. 23
Figure S36. HRESIMS spectroscopic data of erinacenone R. 24
Figure S37. ^{1}H NMR (500 MHz) spectrum of erinacenone R. 25
Figure S38. ^{13}C NMR and DEPT (125 MHz) spectra of erinacenone R. 25
Figure S39. ^{1}H-^{1}H COSY spectrum of erinacenone R. 26
Figure S40. HSQC spectrum of erinacenone R. 26
Figure S41. HMBC spectrum of erinacenone R. 27

Figure S42. ROESY spectrum of erinacenone R. 27
Figure S43. HRESIMS spectroscopic data of erinacenone S. 28
Figure S44. ^{1}H NMR (600 MHz) spectrum of erinacenone S. 29
Figure S45. ^{13}C NMR and DEPT (150 MHz) spectra of erinacenone S. 29
Figure S46. ^{1}H-^{1}H COSY spectrum of erinacenone S. 30
Figure S47. HSQC spectrum of erinacenone S. 30
Figure S48. HMBC spectrum of erinacenone S. 31
Figure S49. ROESY spectrum of erinacenone S. 31
Figure S50. HRESIMS spectroscopic data of erinacenone T. 32
Figure S51. ^{1}H NMR (600 MHz) spectrum of erinacenone T. 32
Figure S52. ^{13}C NMR and DEPT (150 MHz) spectra of erinacenone T. 33
Figure S53. ^{1}H-^{1}H COSY spectrum of erinacenone T. 33
Figure S54. HSQC spectrum of erinacenone T. 34
Figure S55. HMBC spectrum of erinacenone T. 34
Figure S56. ROESY spectrum of erinacenone T. 35
Figure S57. HRESIMS spectroscopic data of erinacenone U. 35
Figure S58. ^{1}H NMR (500 MHz) spectrum of erinacenone U. 36
Figure S59. ^{13}C NMR and DEPT (125 MHz) spectra of erinacenone U. 36
Figure S60. ^{1}H-^{1}H COSY spectrum of erinacenone U. 37
Figure S61. HSQC spectrum of erinacenone U. 37
Figure S62. HMBC spectrum of erinacenone U. 38
Figure S63. ROESY spectrum of erinacenone U. 38
Figure S64. HRESIMS spectroscopic data of erinacenone V. 39
Figure S65. ^{1}H NMR (500 MHz) spectrum of erinacenone V. 40
Figure S66. ^{13}C NMR and DEPT (125 MHz) spectra of erinacenone V. 40
Figure S67. ^{1}H-^{1}H COSY spectrum of erinacenone V. 41
Figure S68. HSQC spectrum of erinacenone V. 41
Figure S69. HMBC spectrum of erinacenone V. 42
Figure S70. ROESY spectrum of erinacenone V. 42
Figure S71. HRESIMS spectroscopic data of erinacenone W. 43
Figure S72. ^{1}H NMR (600 MHz) spectrum of erinacenone W. 44
Figure S73. ^{13}C NMR and DEPT (150 MHz) spectra of erinacenone W. 44
Figure S74. ^{1}H-^{1}H COSY spectrum of erinacenone W. 45
Figure S75. HSQC spectrum of erinacenone W. 45
Figure S76. HMBC spectrum of erinacenone W. 46
Figure S77. ROESY spectrum of erinacenone W. 46
Figure S78. HRESIMS spectroscopic data of erinacenone X. 47
Figure S79. ^{1}H NMR (600 MHz) spectrum of erinacenone X. 48
Figure S80. ^{13}C NMR and DEPT (150 MHz) spectra of erinacenone X. 48
Figure S81. ^{1}H-^{1}H COSY spectrum of erinacenone X. 49
Figure S82. HSQC spectrum of erinacenone X. 49
Figure S83. HMBC spectrum of erinacenone X. 50
Figure S84. ROESY spectrum of erinacenone X. 50
Figure S85. HRESIMS spectroscopic data of erinacenone Y. 51

Figure S86. ^{1}H NMR (600 MHz) spectrum of erinacenone Y. 52
Figure S87. ^{13}C NMR and DEPT (150 MHz) spectra of erinacenone Y. 52
Figure S88. ^{1}H-^{1}H COSY spectrum of erinacenone Y. 53
Figure S89. HSQC spectrum of erinacenone Y. 53
Figure S90. HMBC spectrum of erinacenone Y. 54
Figure S91. ROESY spectrum of erinacenone Y. 54
Figure S92. HRESIMS spectroscopic data of compound 14. 55
Figure S93. HRESIMS spectroscopic data of compound 15. 55
Figure S94. ^{1}H NMR (600 MHz) spectrum of compound 14. 56
Figure S95. ^{13}C NMR and DEPT (150 MHz) spectra of compound 14. 56
Figure S96. HRESIMS spectroscopic data of compound 16. 57
Figure S97. HRESIMS spectroscopic data of compound 17. 57
Figure S98. ^{1}H NMR (600 MHz) spectrum of compound 16. 58
Figure S99. ^{13}C NMR and DEPT (150 MHz) spectra of compound 16. 58
Figure S100. HRESIMS spectroscopic data of compound 18. 59
Figure S101. HRESIMS spectroscopic data of compound 19. 59
Figure S102. ^{1}H NMR (600 MHz) spectrum of compound 18. 60
Figure S103. ^{13}C NMR and DEPT (150 MHz) spectra of compound 18. 60
Figure S104. HRESIMS spectroscopic data of compound 20. 61
Figure S105. HRESIMS spectroscopic data of compound 21. 61
Figure S106. ^{1}H NMR (600 MHz) spectrum of compound 20. 62
Figure S107. ^{13}C NMR and DEPT (150 MHz) spectra of compound 20. 62
Figure S108. HRESIMS spectroscopic data of compound 22. 63
Figure S109. HRESIMS spectroscopic data of compound 23. 63
Figure S110. ^{1}H NMR (600 MHz) spectrum of compound 22. 64
Figure S111. ^{13}C NMR and DEPT (150 MHz) spectra of compound 22. 64
Table S1 Cytotoxicity of compounds (in IC_{50}, μM).[a] 65

Figure S1. HRESIMS spectroscopic data of erinacenone M.

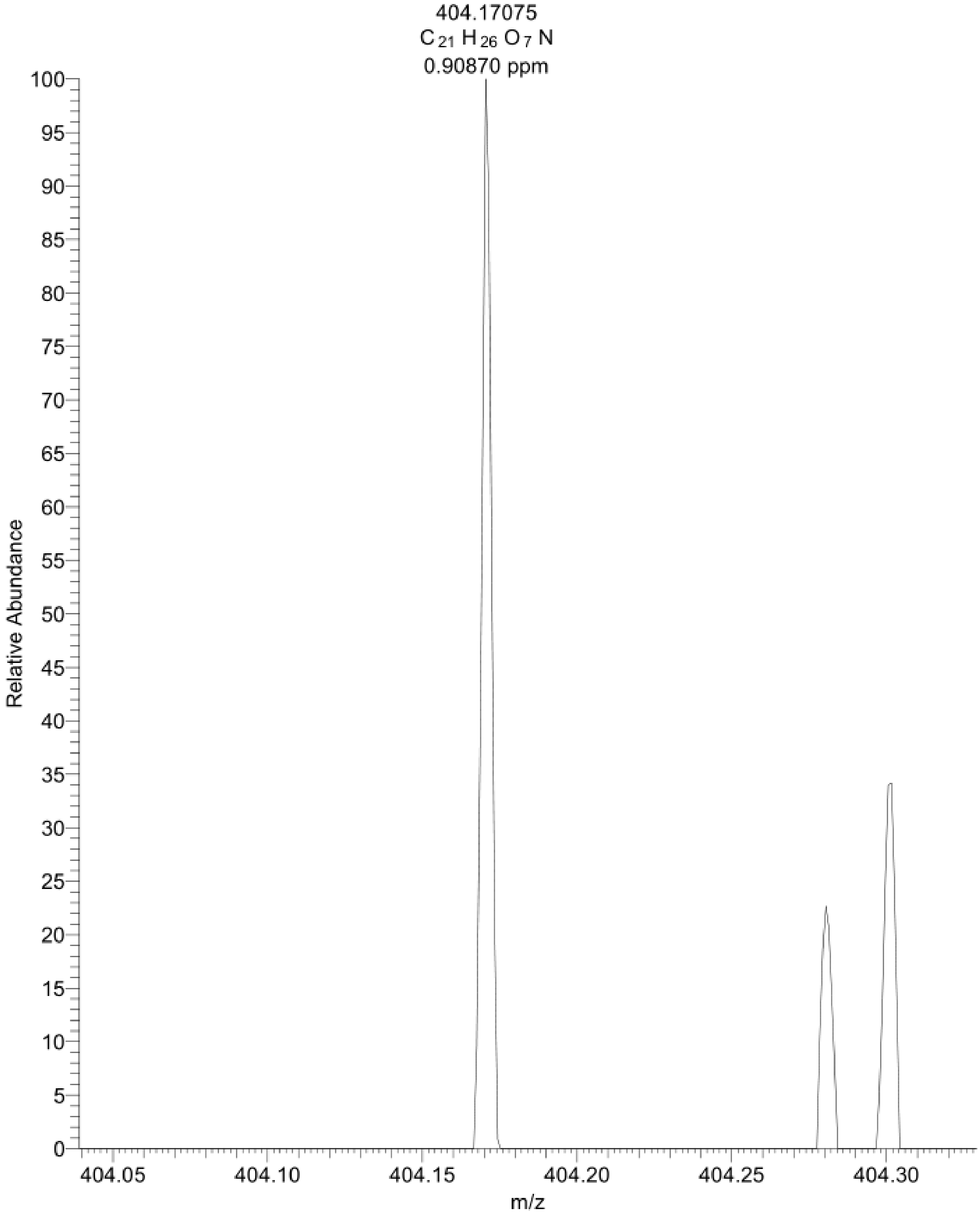

Figure S2. ^{1}H NMR (600 MHz) spectrum of erinacenone M.

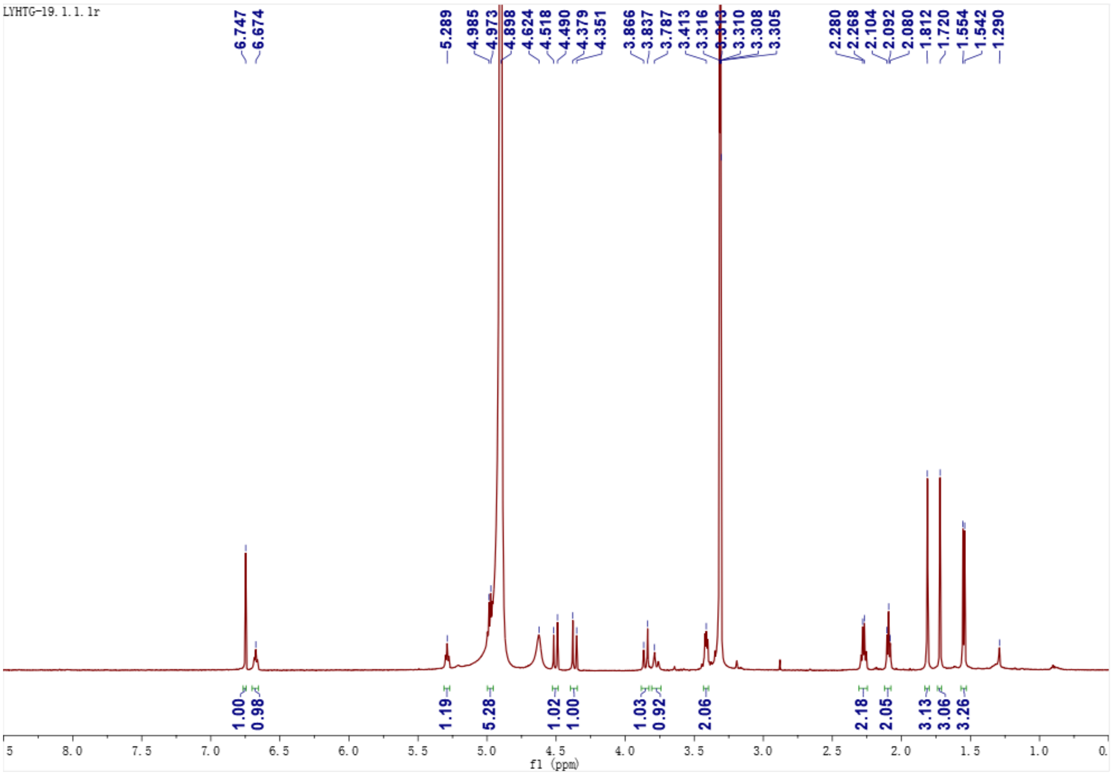

Figure S3. ^{13}C NMR and DEPT (150 MHz) spectra of erinacenone M.

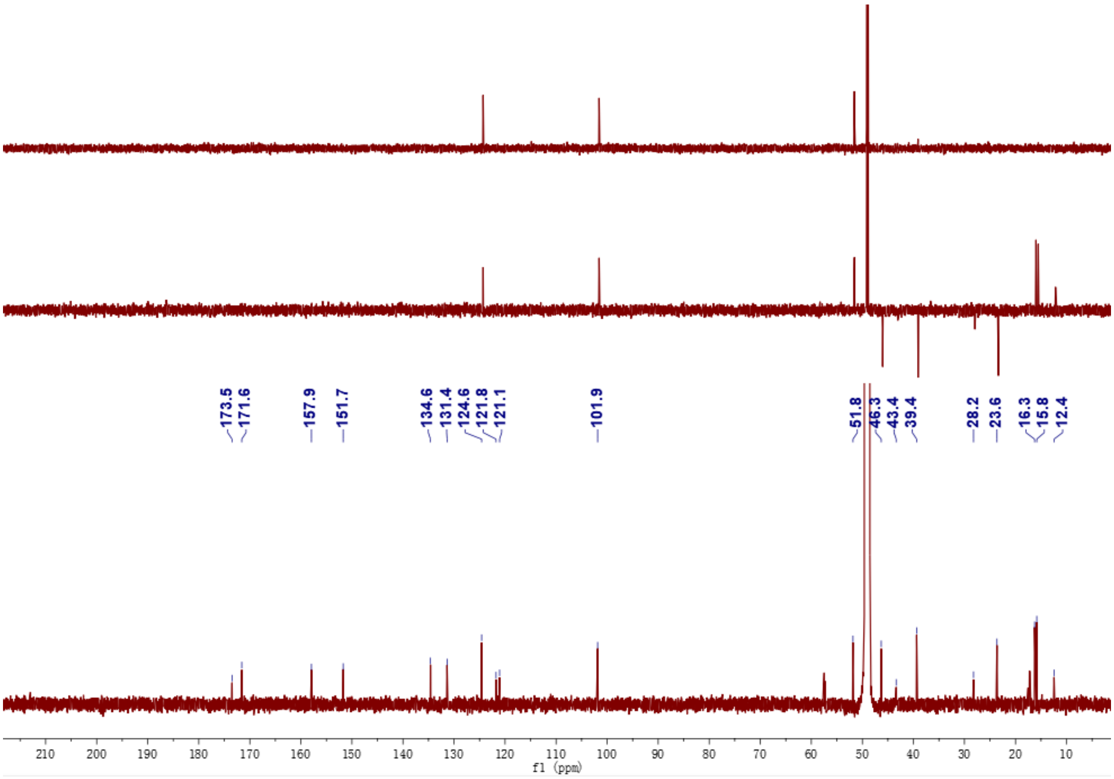

Figure S4. ^{1}H-^{1}H COSY spectrum of erinacenone M.

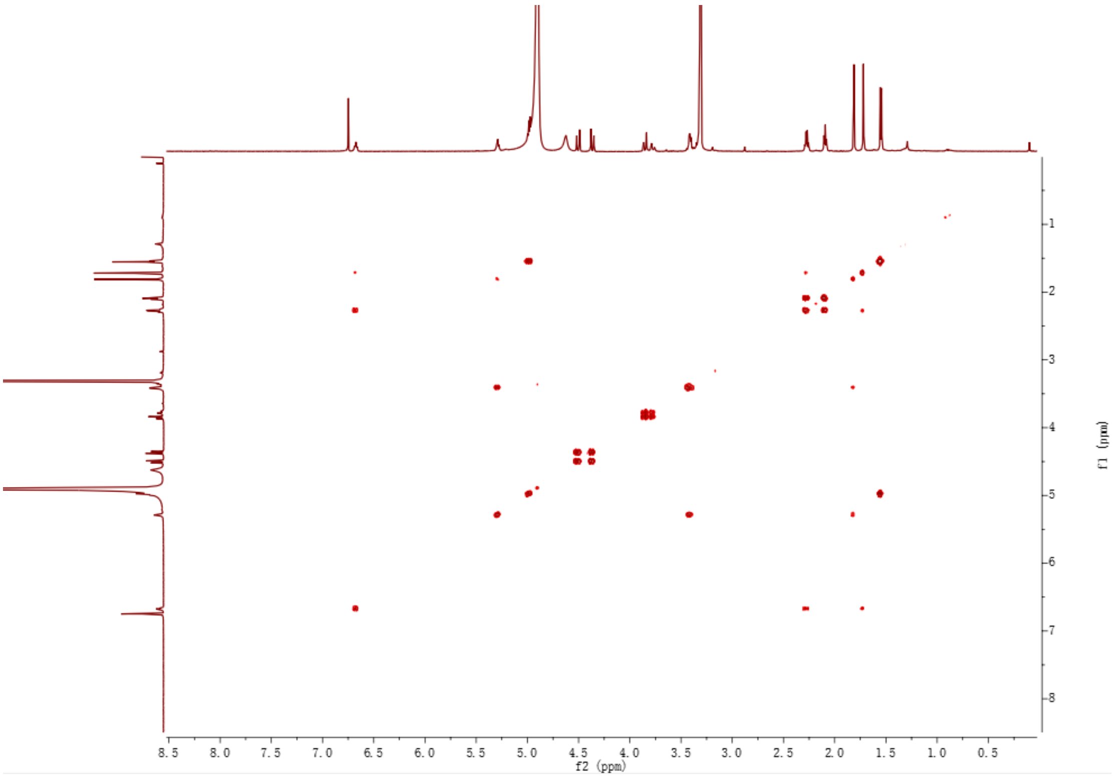

Figure S5. HSQC spectrum of erinacenone M.

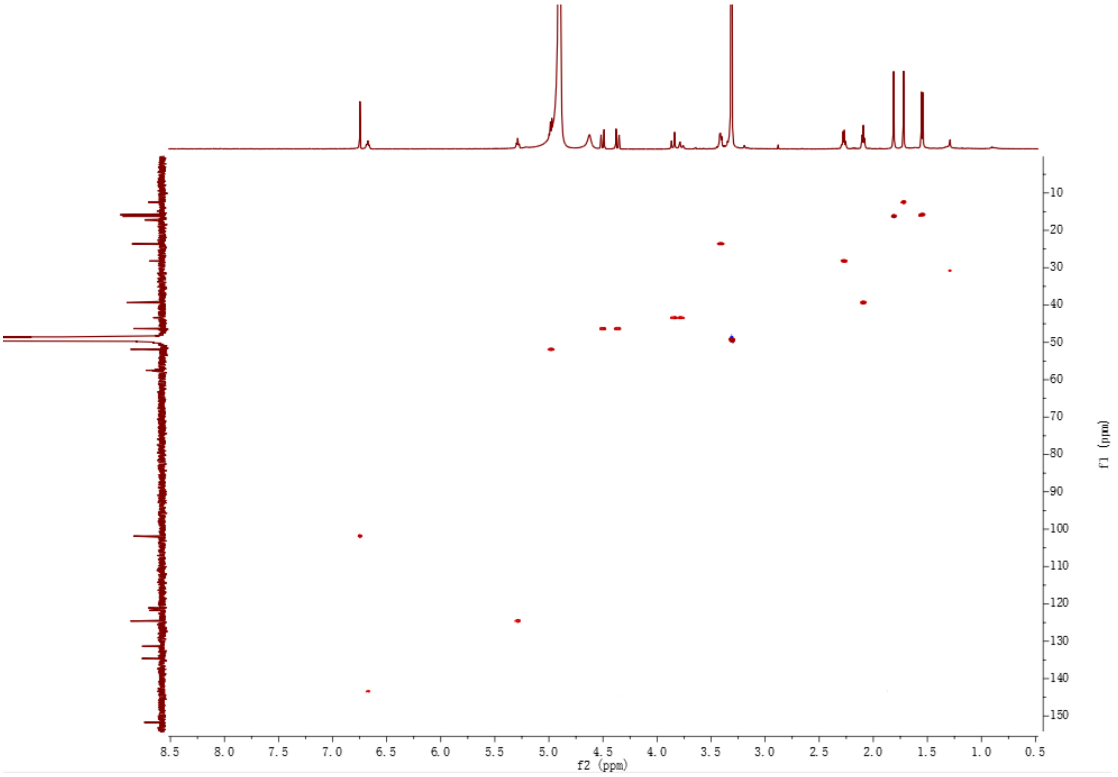

Figure S6. HMBC spectrum of erinacenone M.

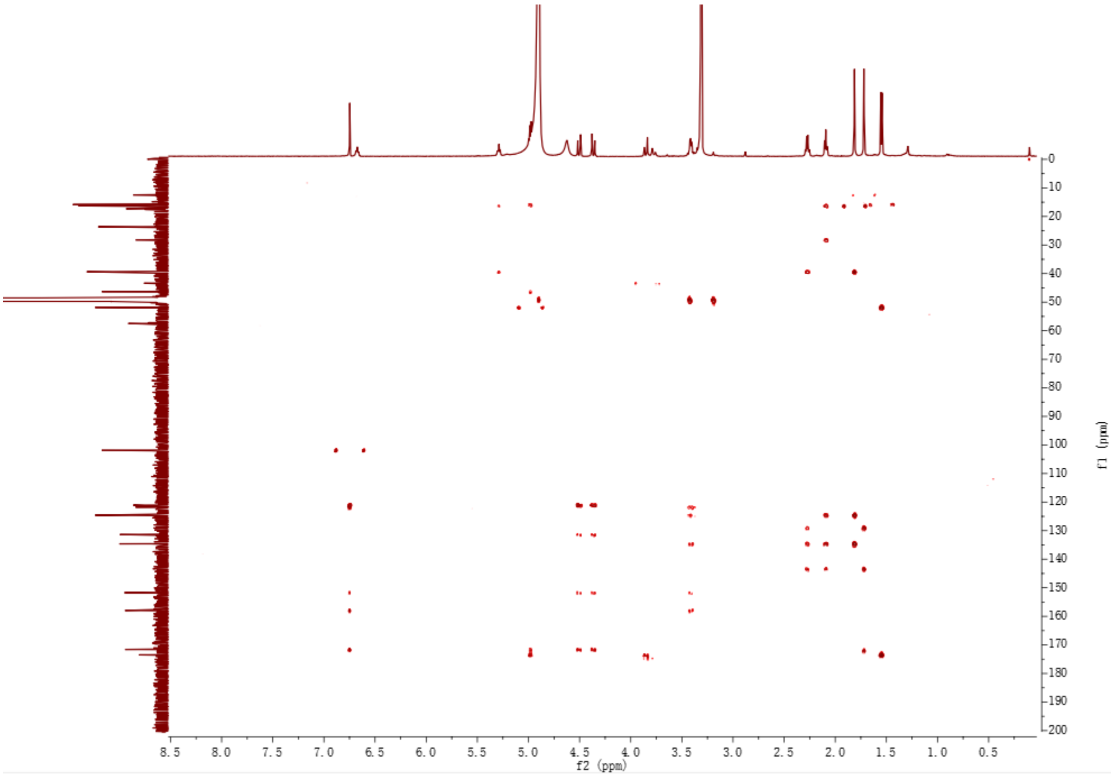

Figure S7. ROESY spectrum of erinacenone M.

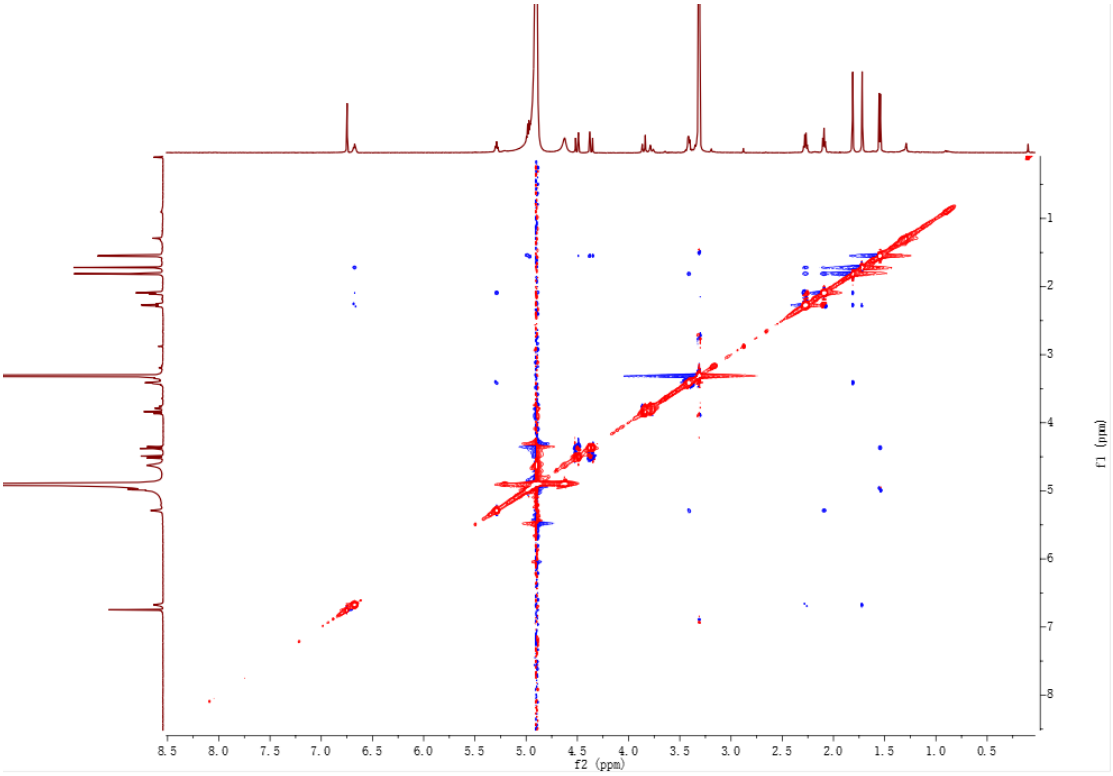

Figure S8. HRESIMS spectroscopic data of erinacenone N.

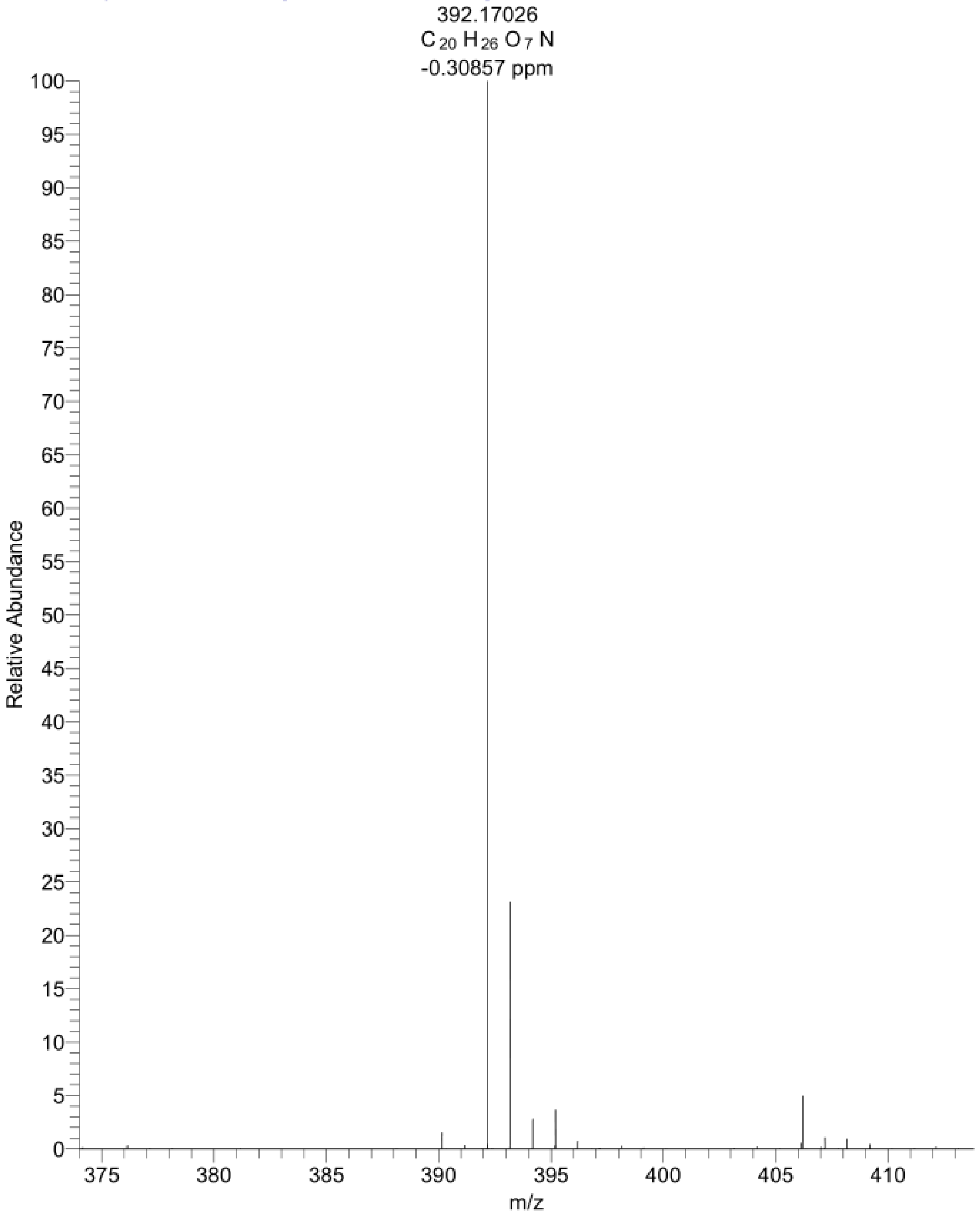

Figure S9. ^{1}H NMR (500 MHz) spectrum of erinacenone N.

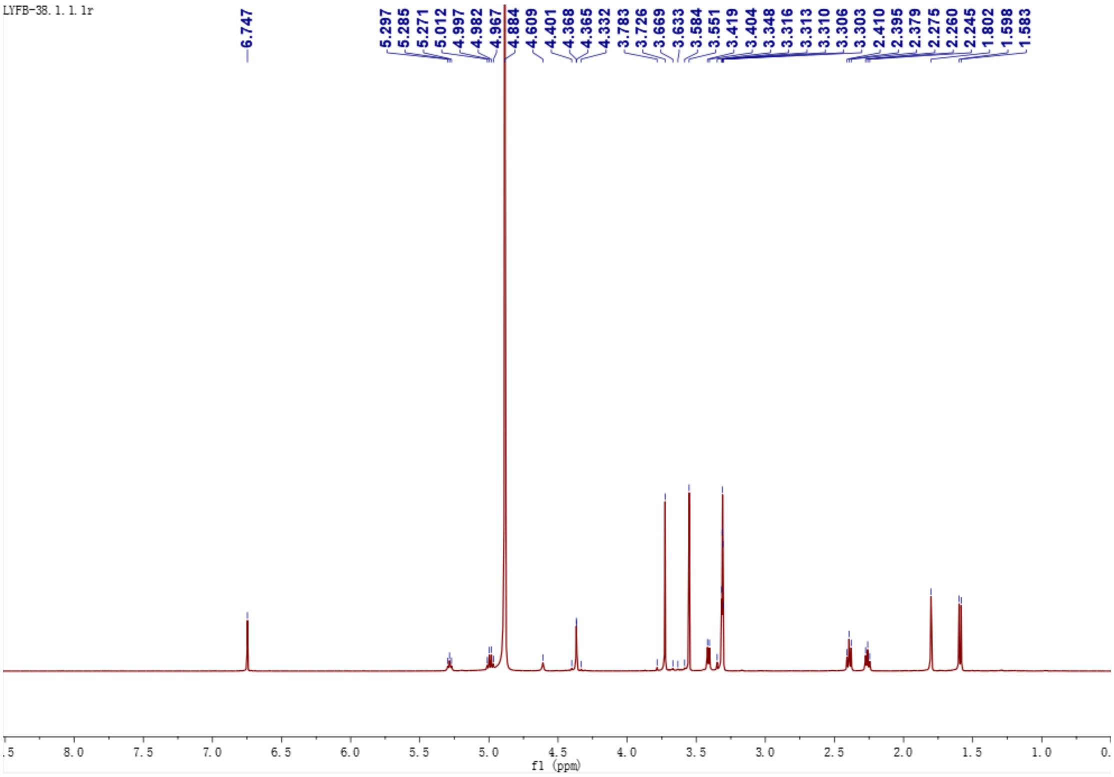

Figure S10. ^{13}C NMR and DEPT (125 MHz) spectra of erinacenone N.

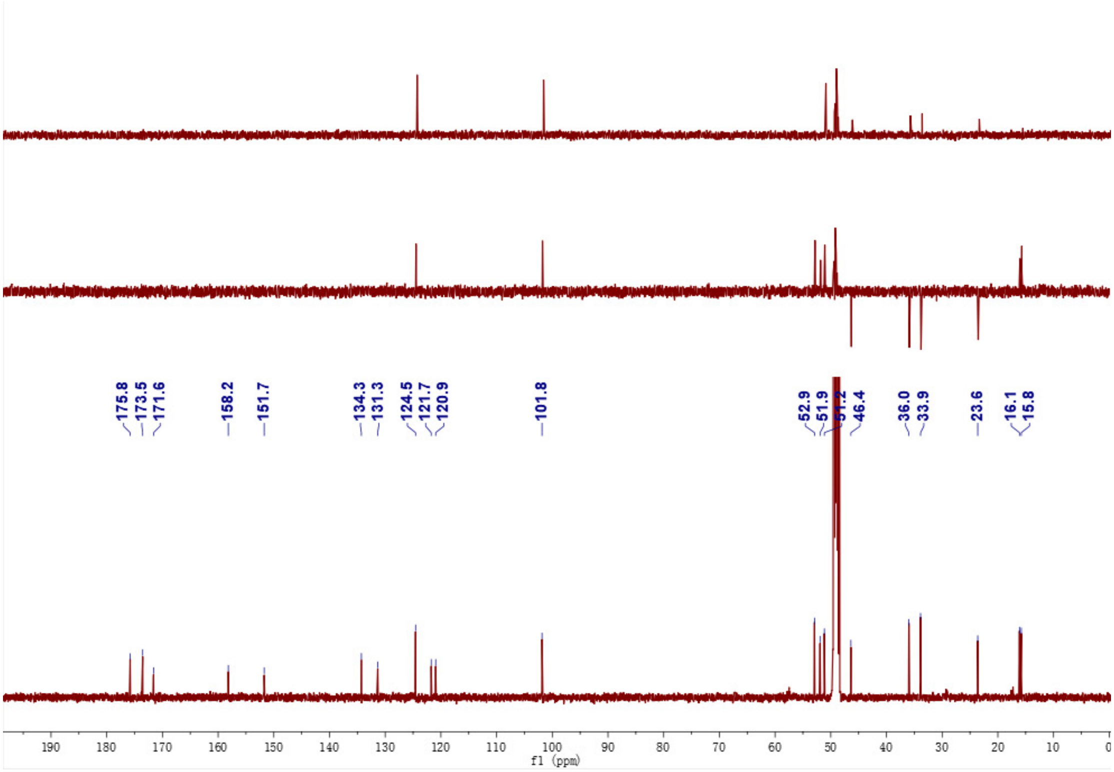

Figure S11. ^{1}H-^{1}H COSY spectrum of erinacenone N.

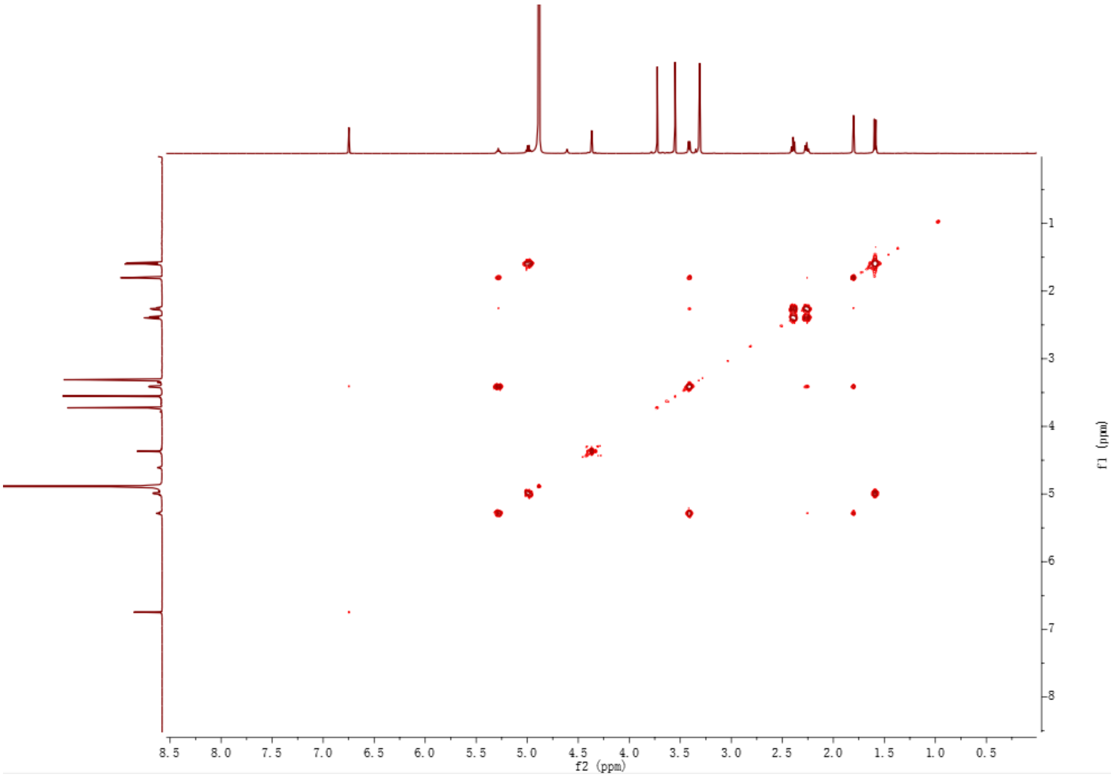

Figure S12. HSQC spectrum of erinacenone N.

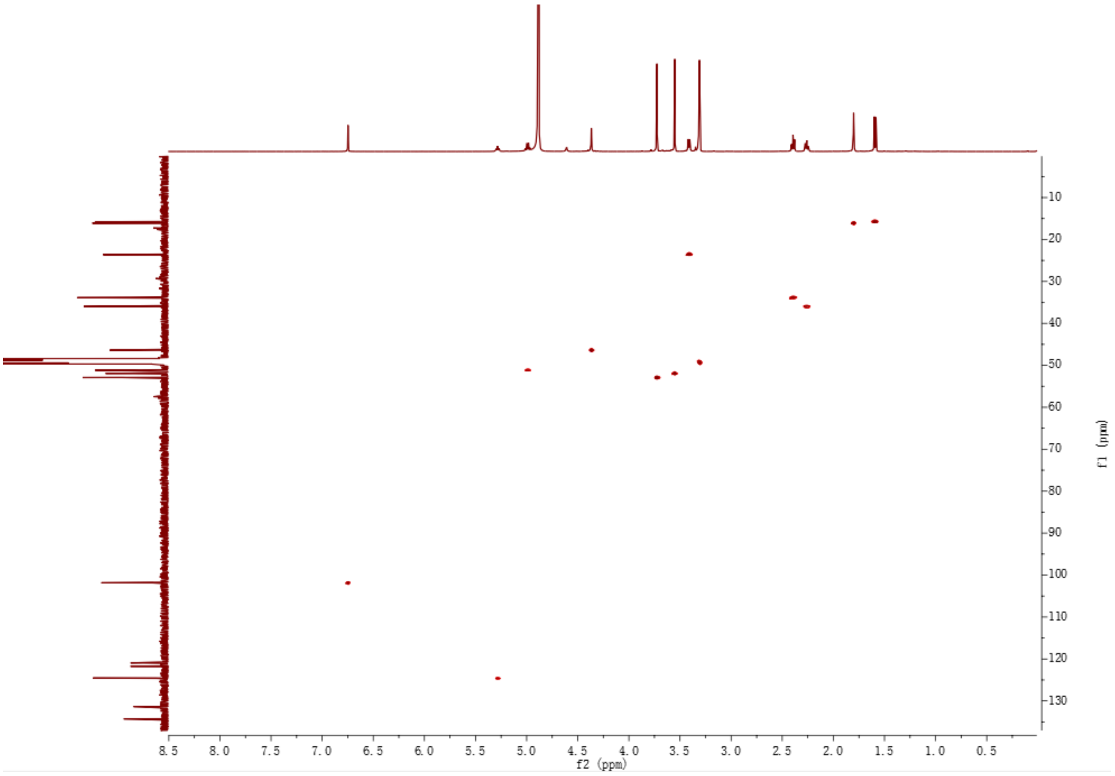

Figure S13. HMBC spectrum of erinacenone N.

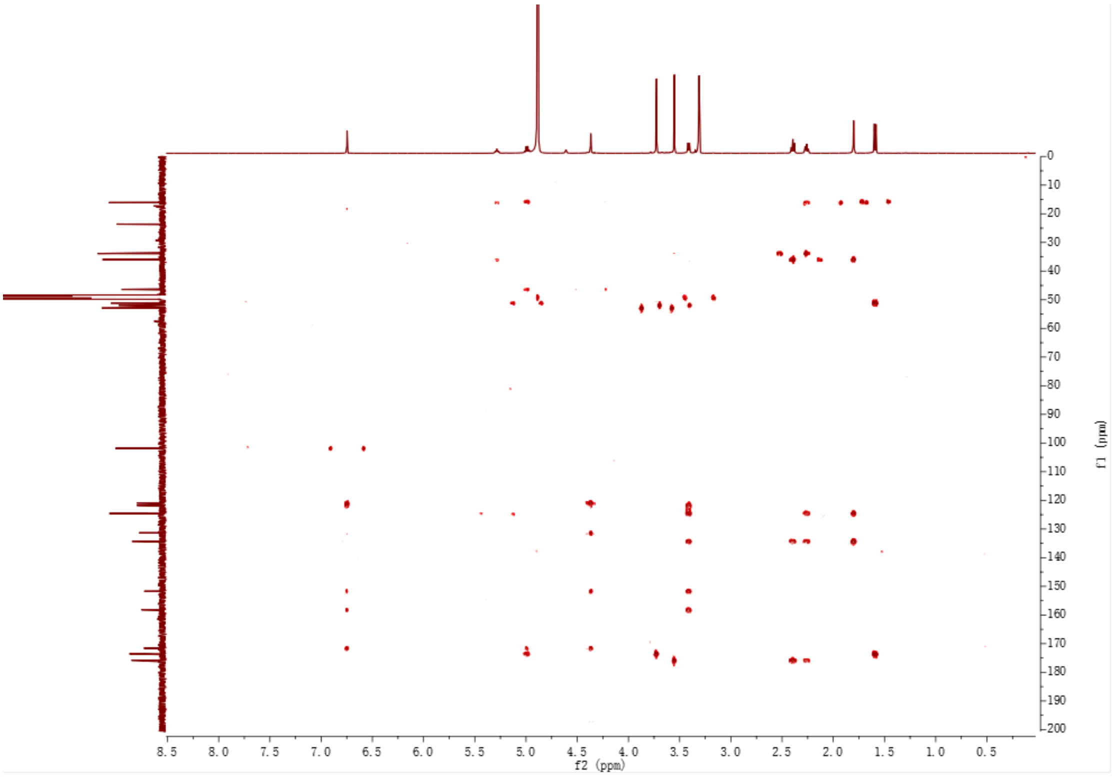

Figure S14. ROESY spectrum of erinacenone N.

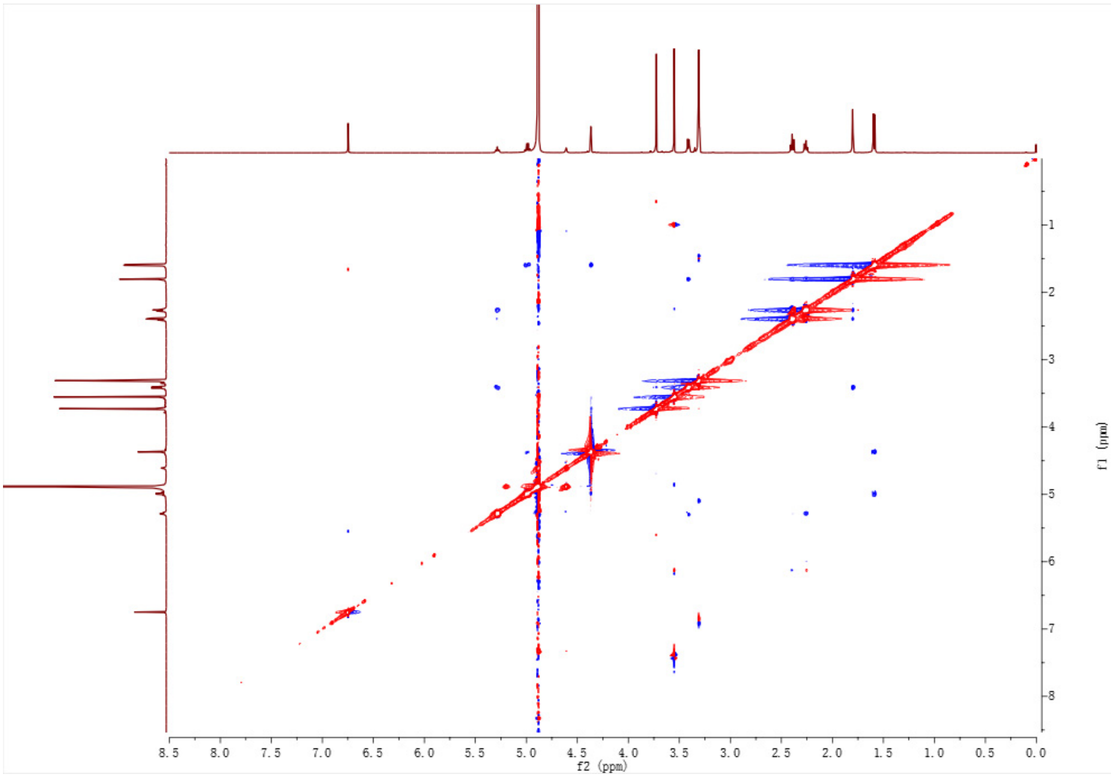

Figure S15. HRESIMS spectroscopic data of erinacenone O.

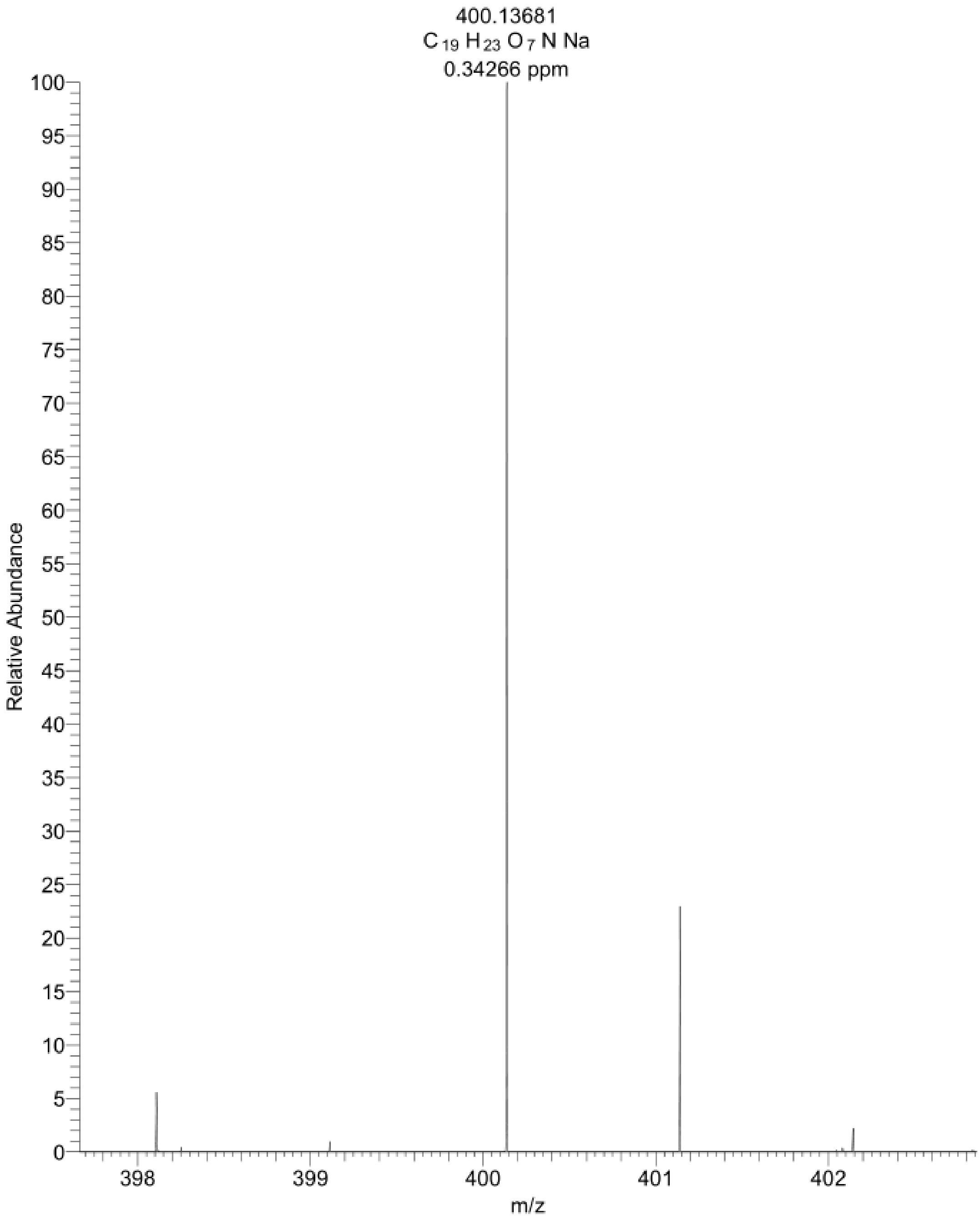

Figure S16. ^{1}**H NMR (600 MHz) spectrum of erinacenone O.**

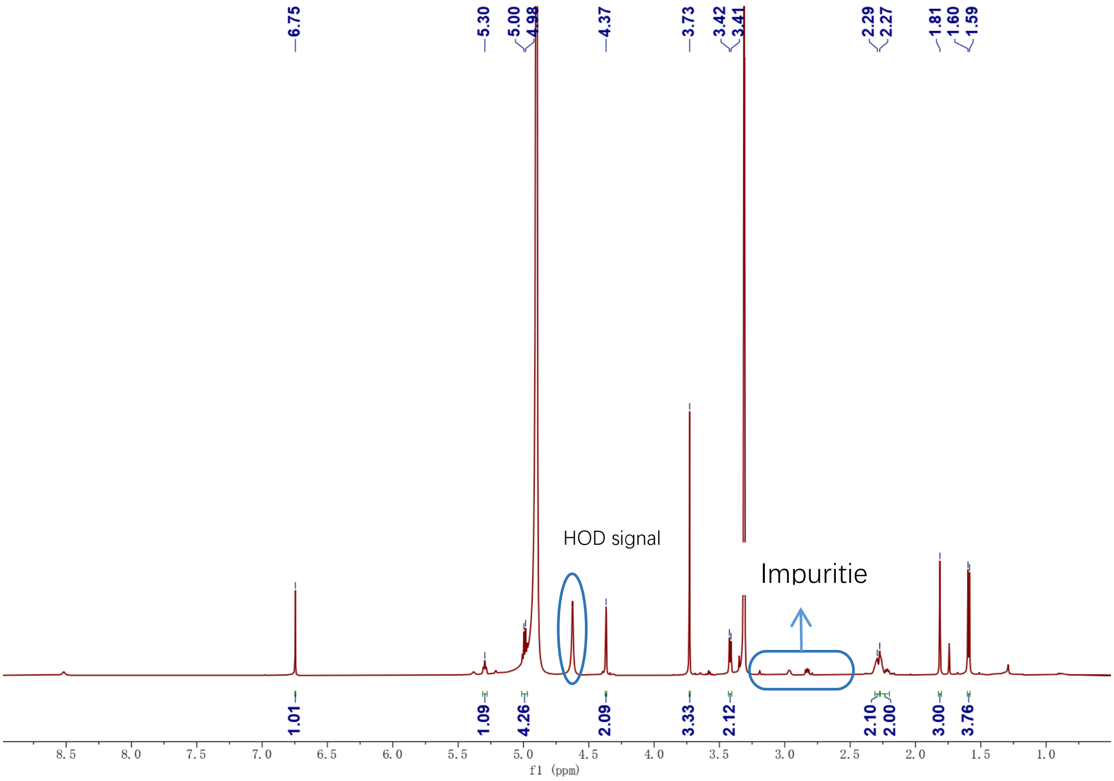

Figure S17. ^{13}**C NMR and DEPT (150 MHz) spectra of erinacenone O.**

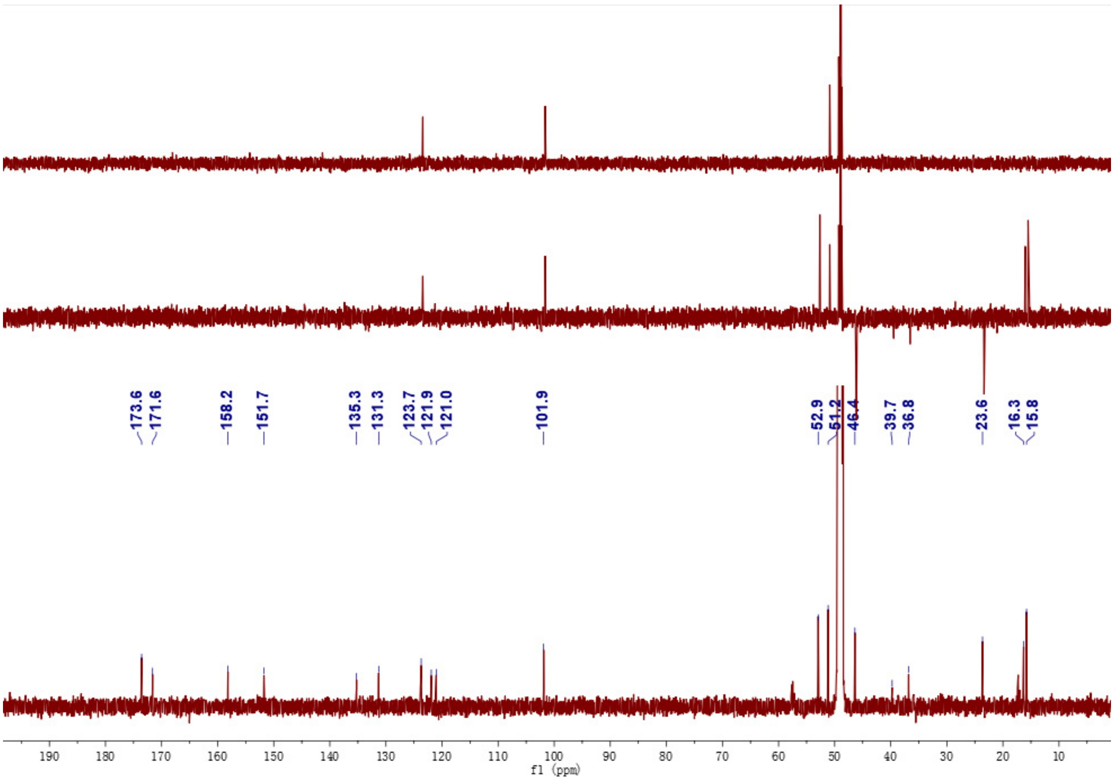

Figure S18. ^1H-^1H COSY spectrum of erinacenone O.

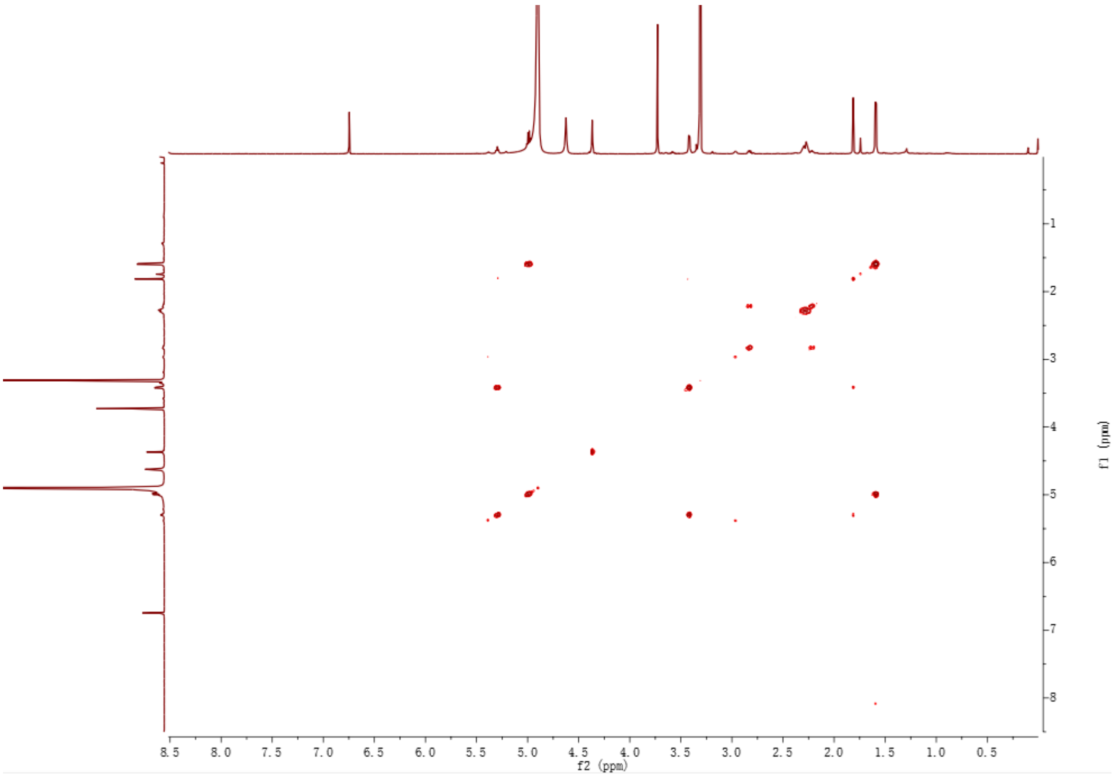

Figure S19. HSQC spectrum of erinacenone O.

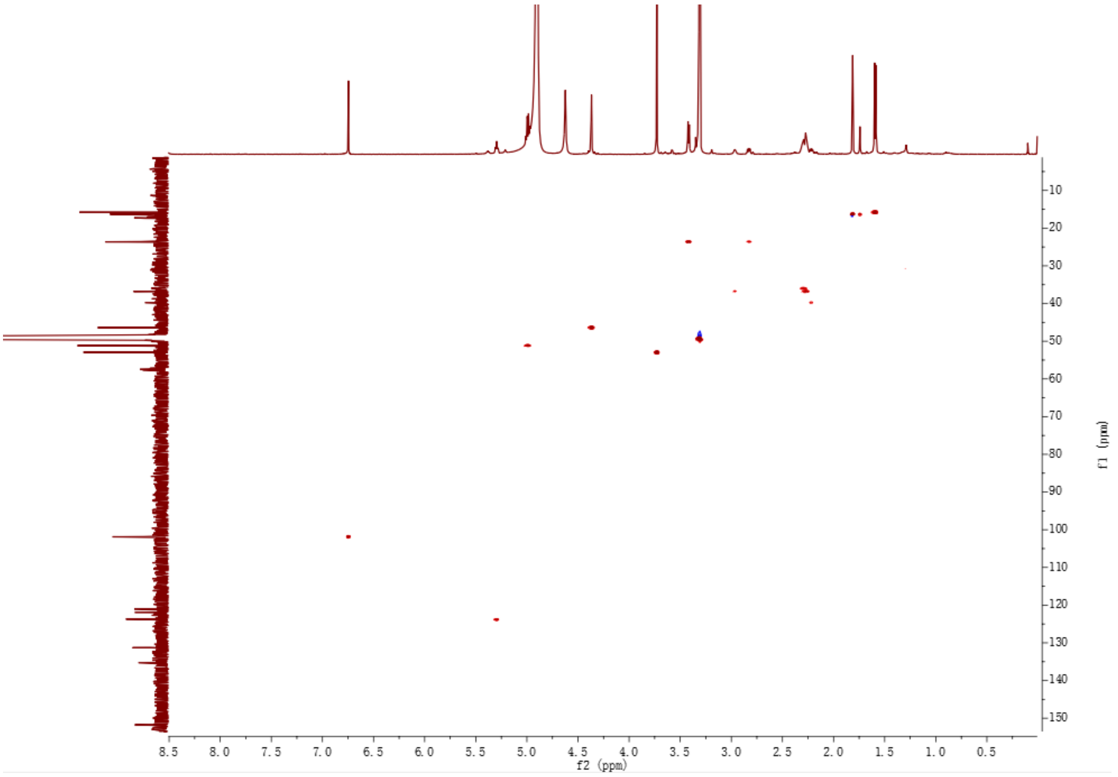

Figure S20. HMBC spectrum of erinacenone O.

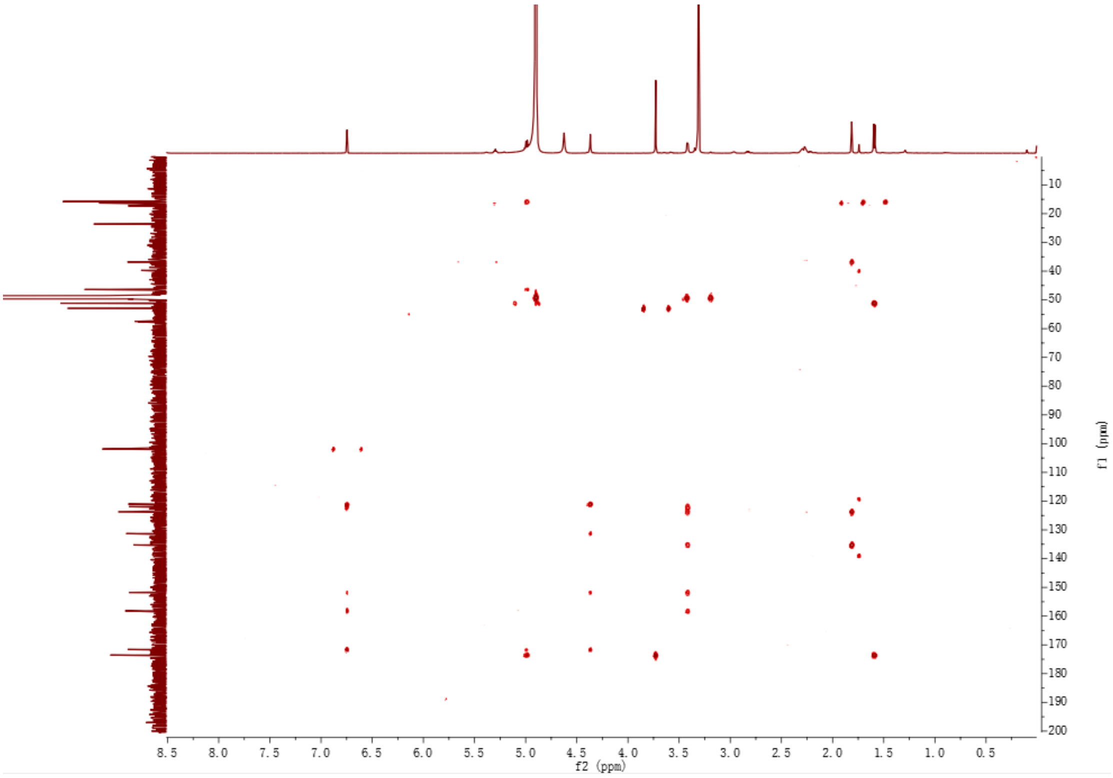

Figure S21. ROESY spectrum of erinacenone O.

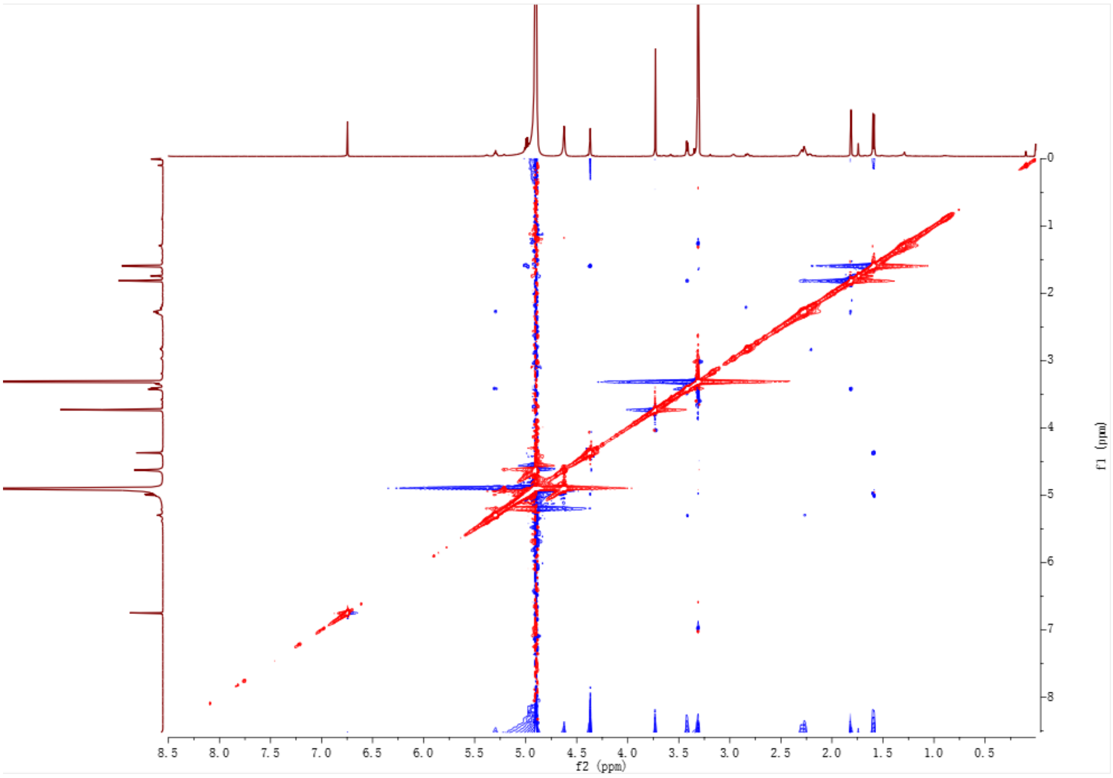

Figure S22. HRESIMS spectroscopic data of erinacenone P.

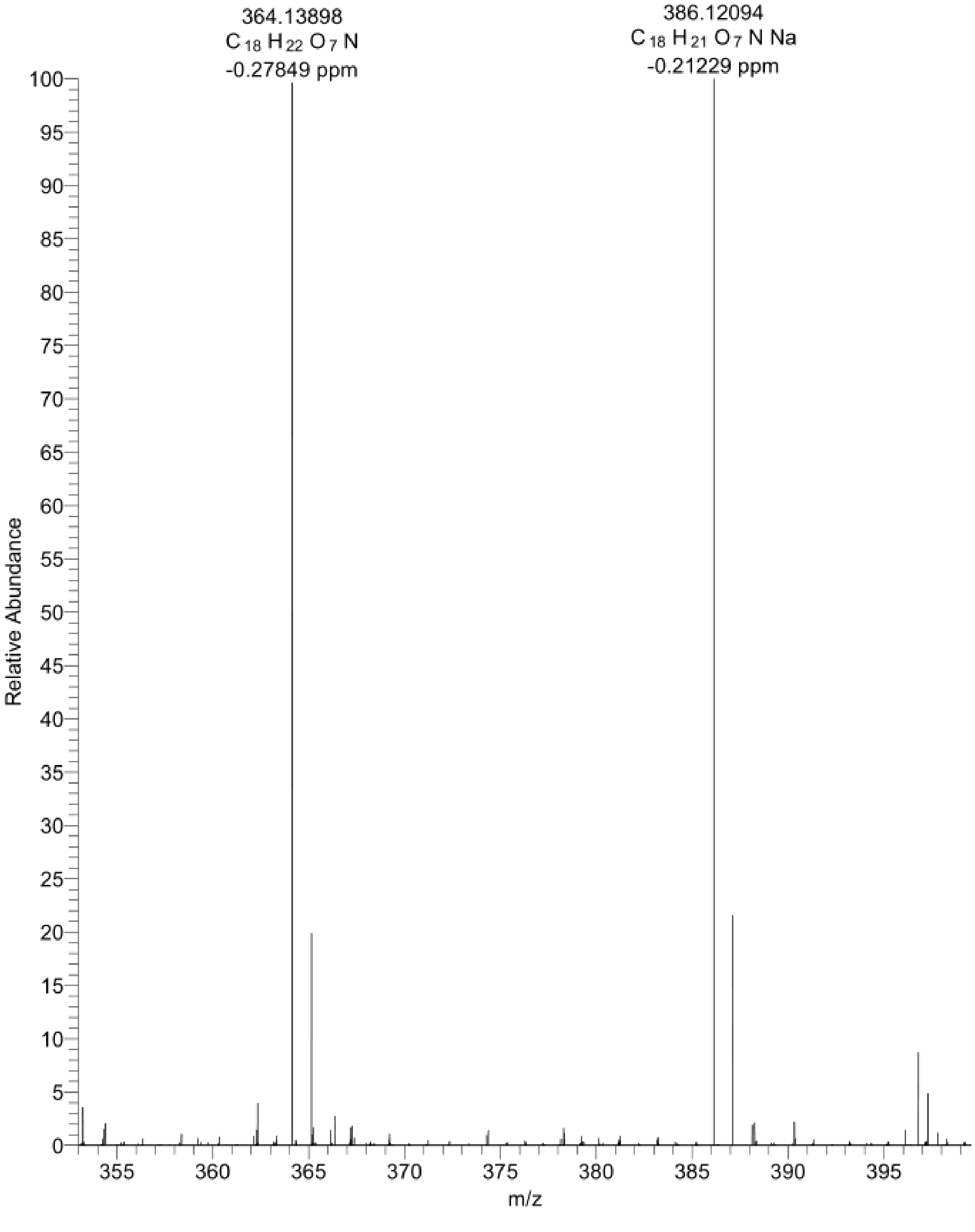

Figure S23. ^{1}H NMR (600 MHz) spectrum of erinacenone P.

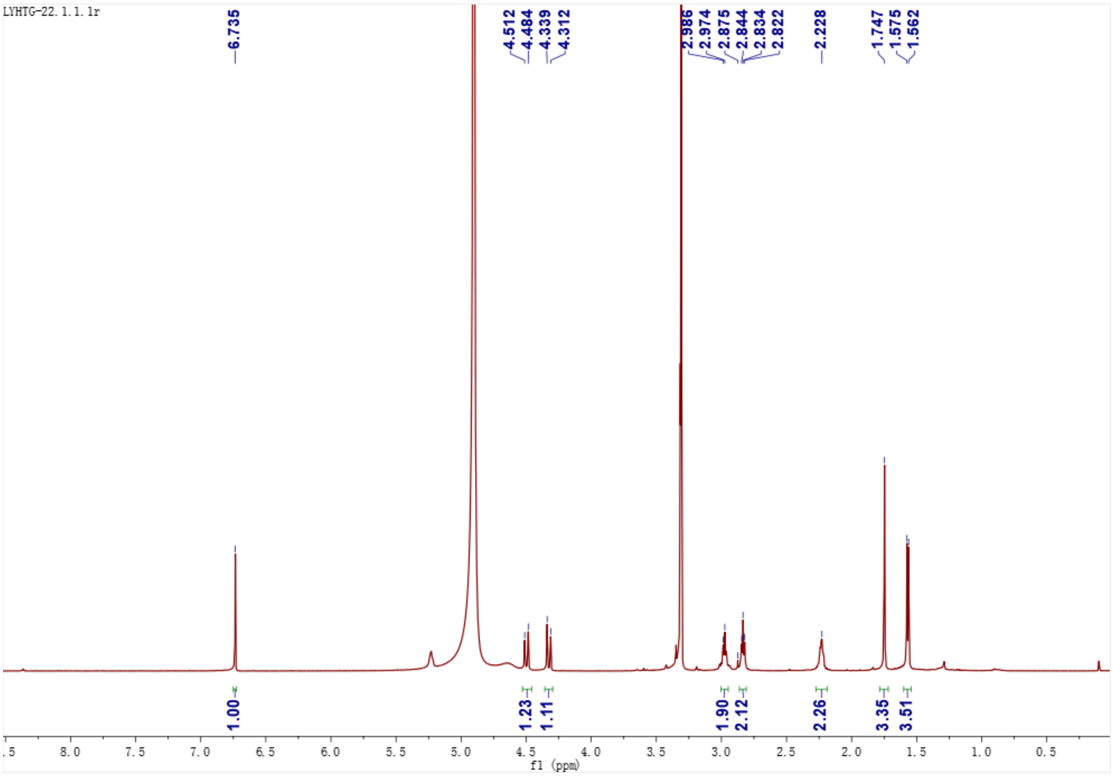

Figure S24. ^{13}C NMR and DEPT (150 MHz) spectra of erinacenone P.

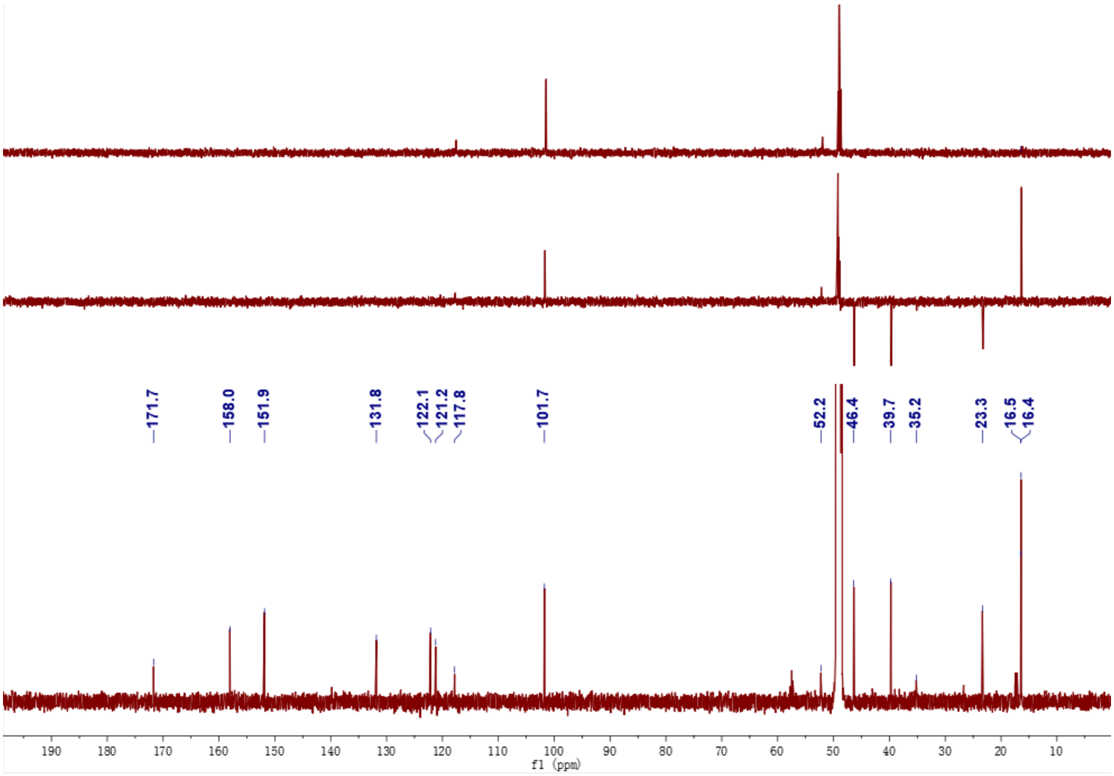

Figure S25. ¹H-¹H COSY spectrum of erinacenone P.

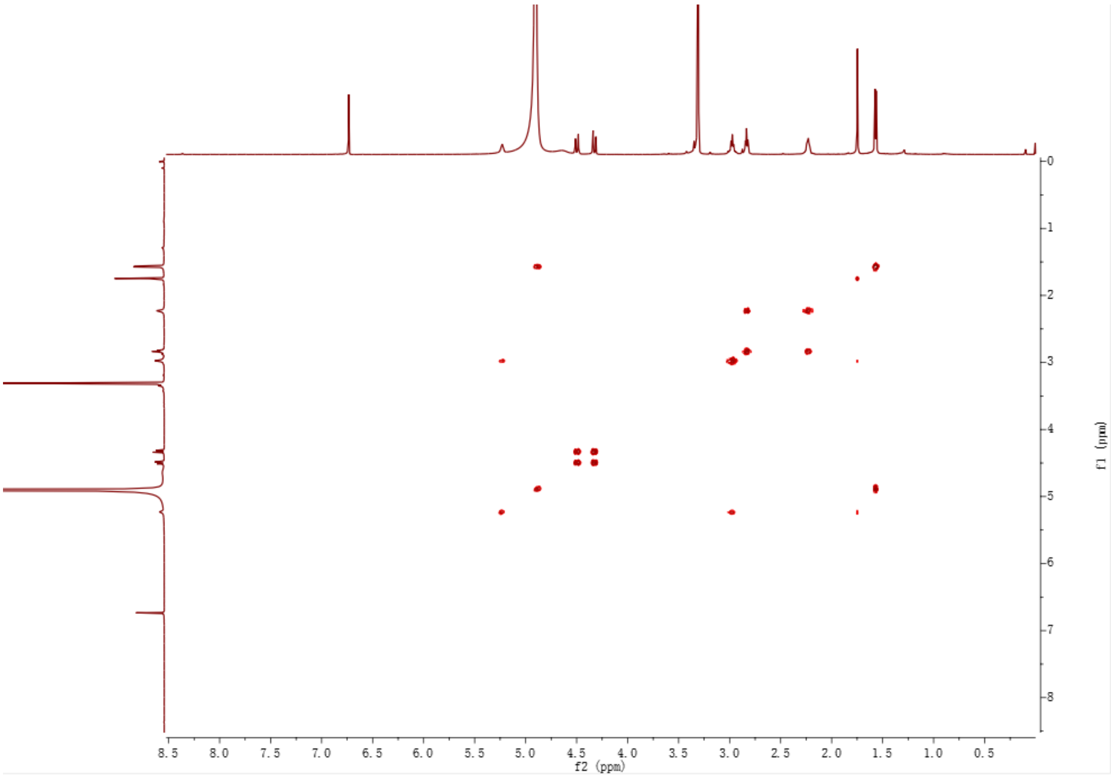

Figure S26. HSQC spectrum of erinacenone P.

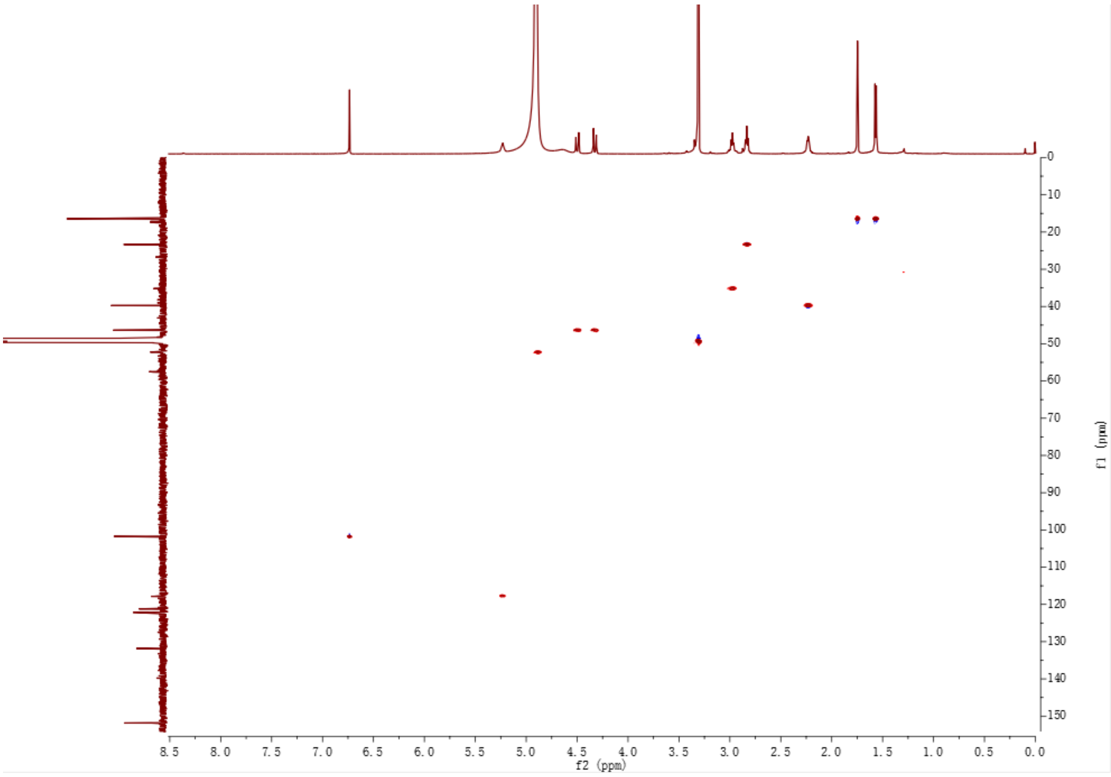

Figure S27. HMBC spectrum of erinacenone P.

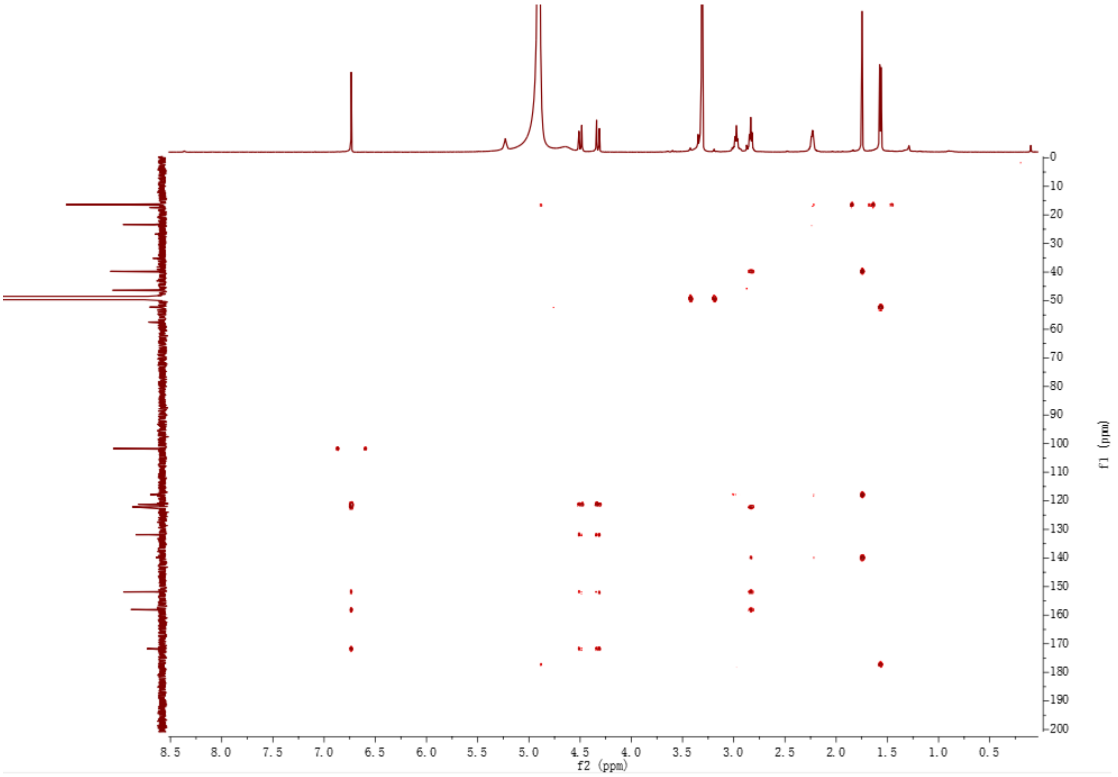

Figure S28. ROESY spectrum of erinacenone P.

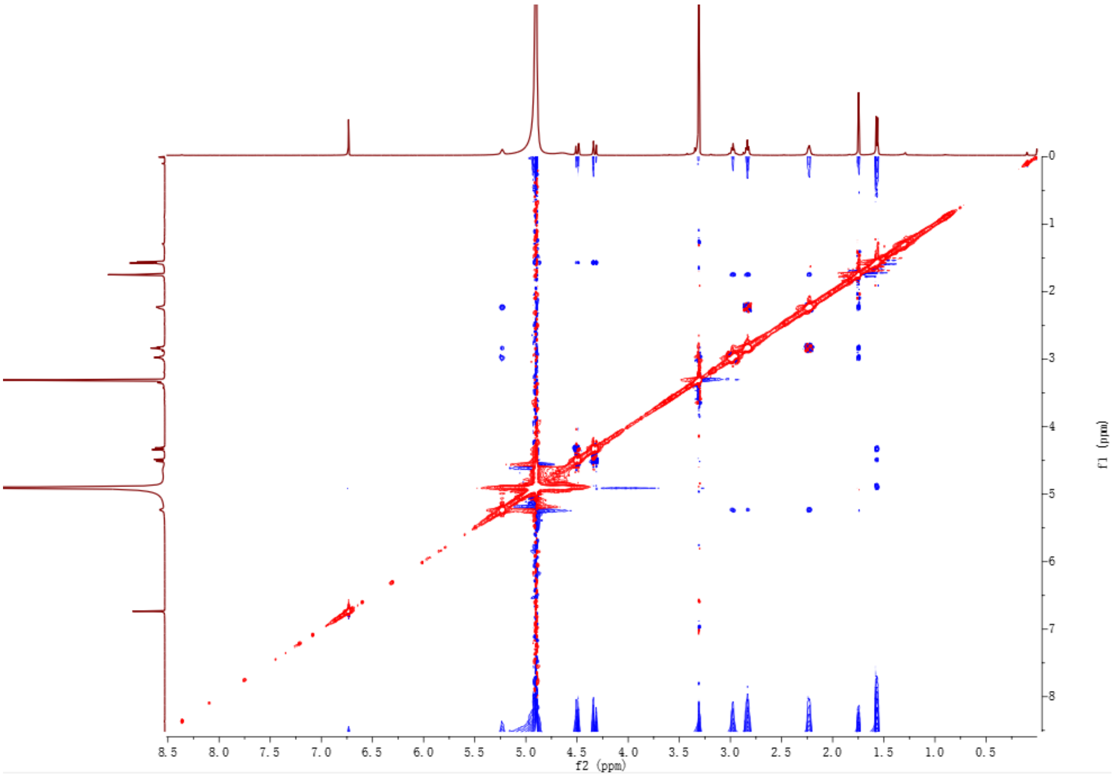

Figure S29. HRESIMS spectroscopic data of erinacenone Q.

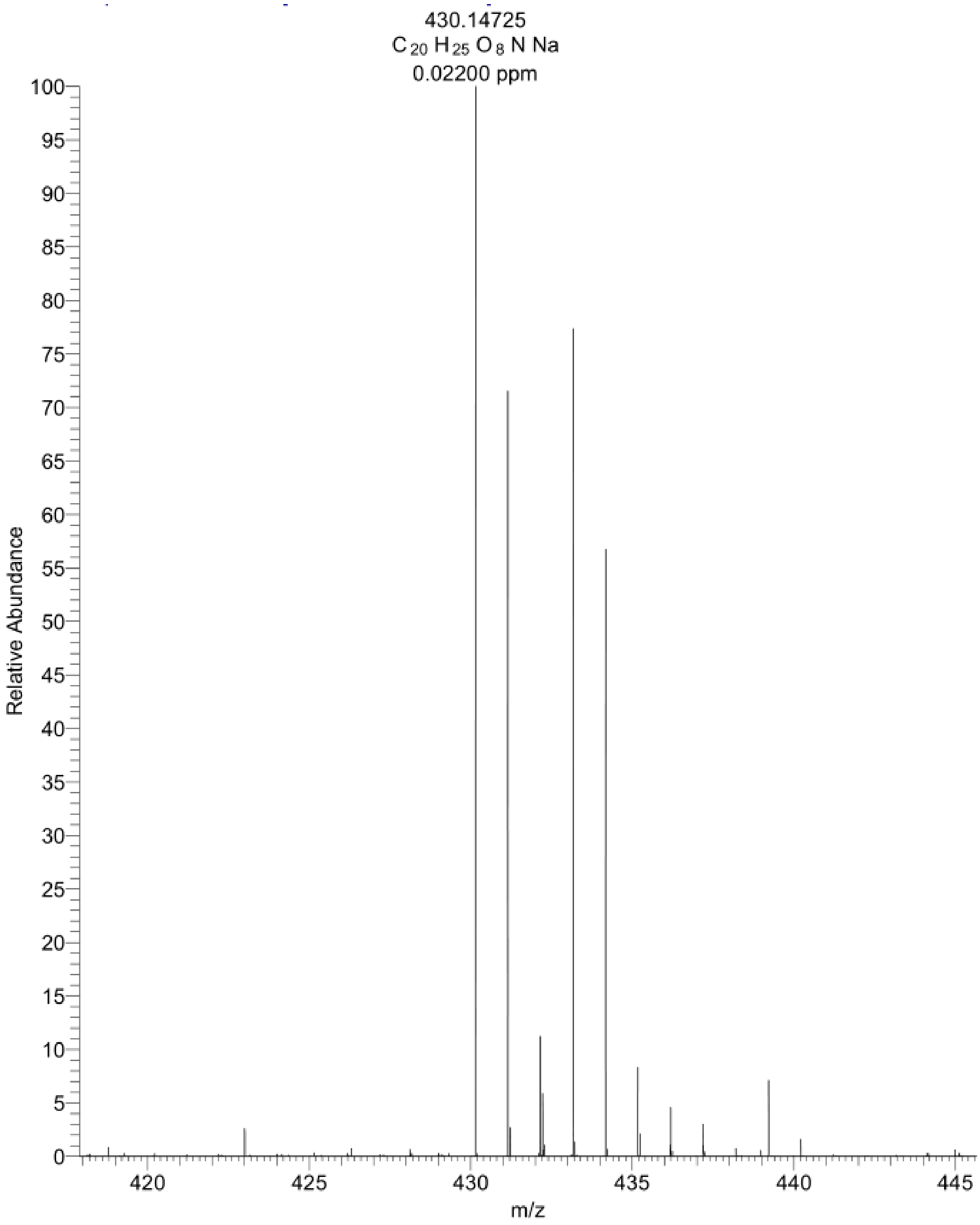

Figure S30. ^{1}H NMR (500 MHz) spectrum of erinacenone Q.

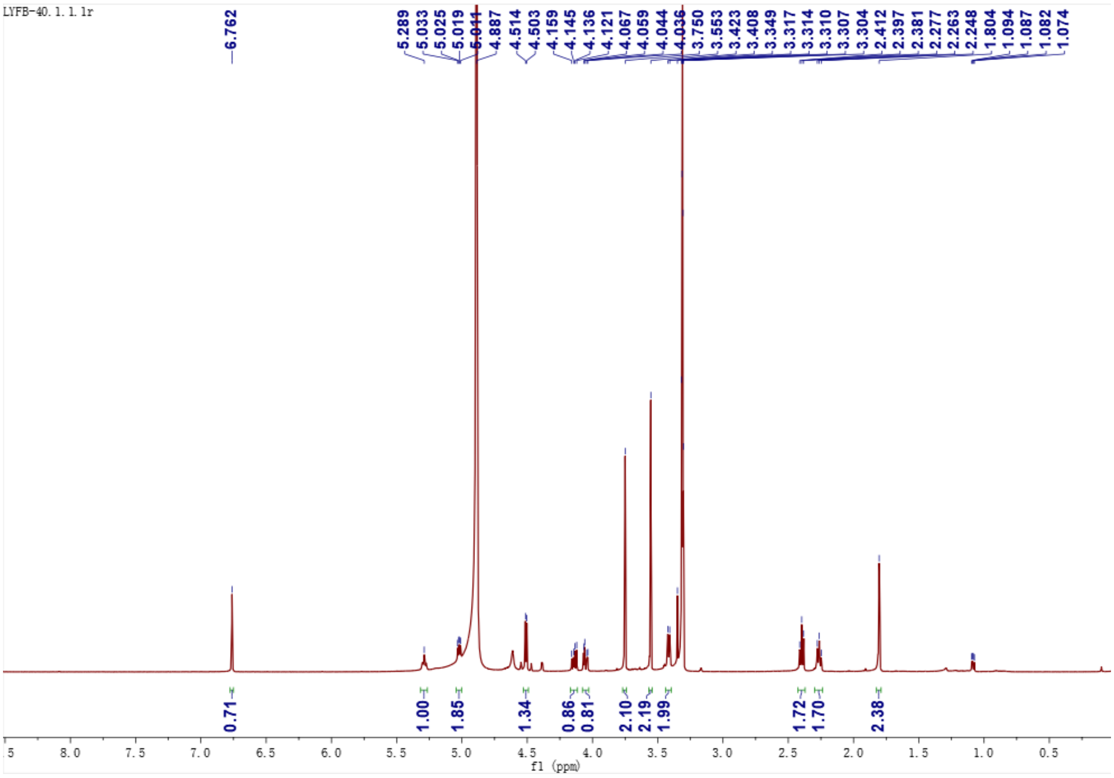

Figure S31. ^{13}C NMR and DEPT (125 MHz) spectra of erinacenone Q.

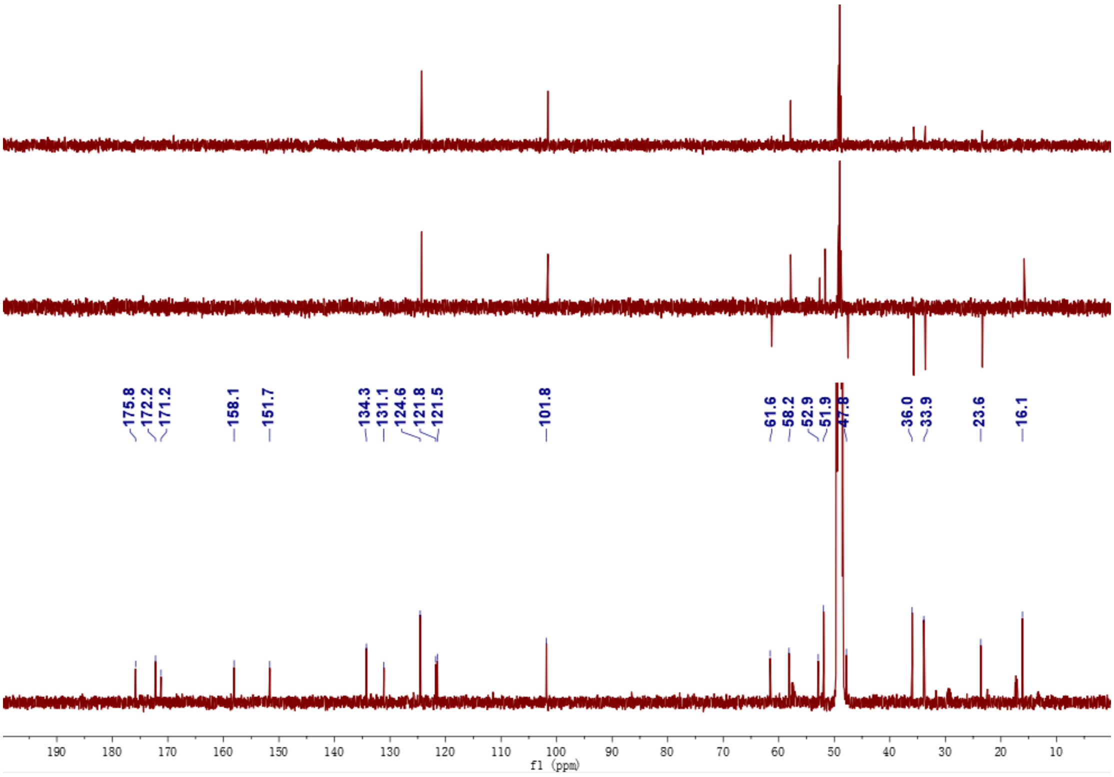

Figure S32. ^{1}H-^{1}H COSY spectrum of erinacenone Q.

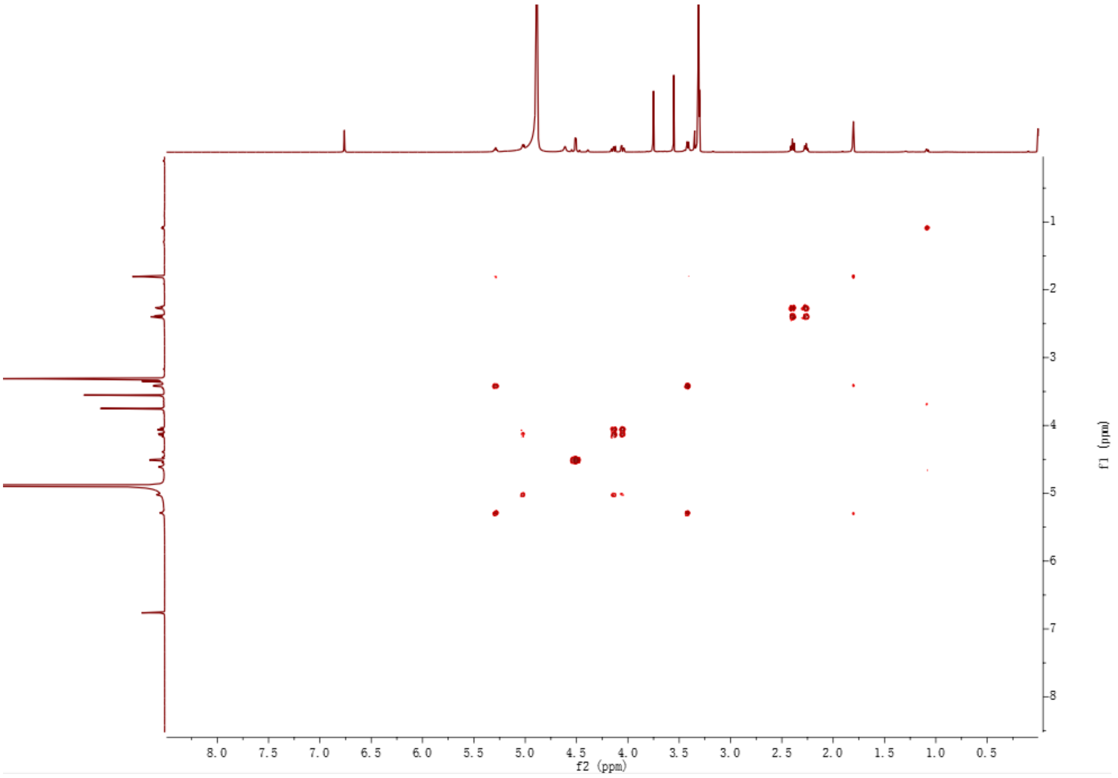

Figure S33. HSQC spectrum of erinacenone Q.

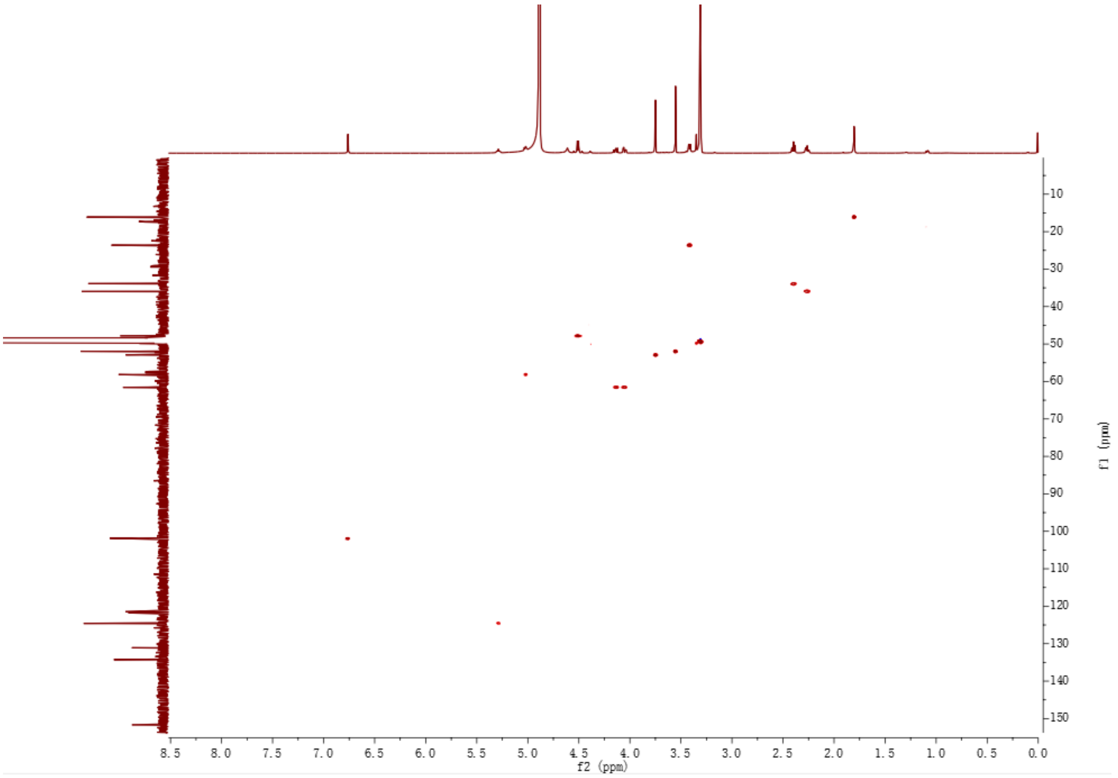

Figure S34. HMBC spectrum of erinacenone Q.

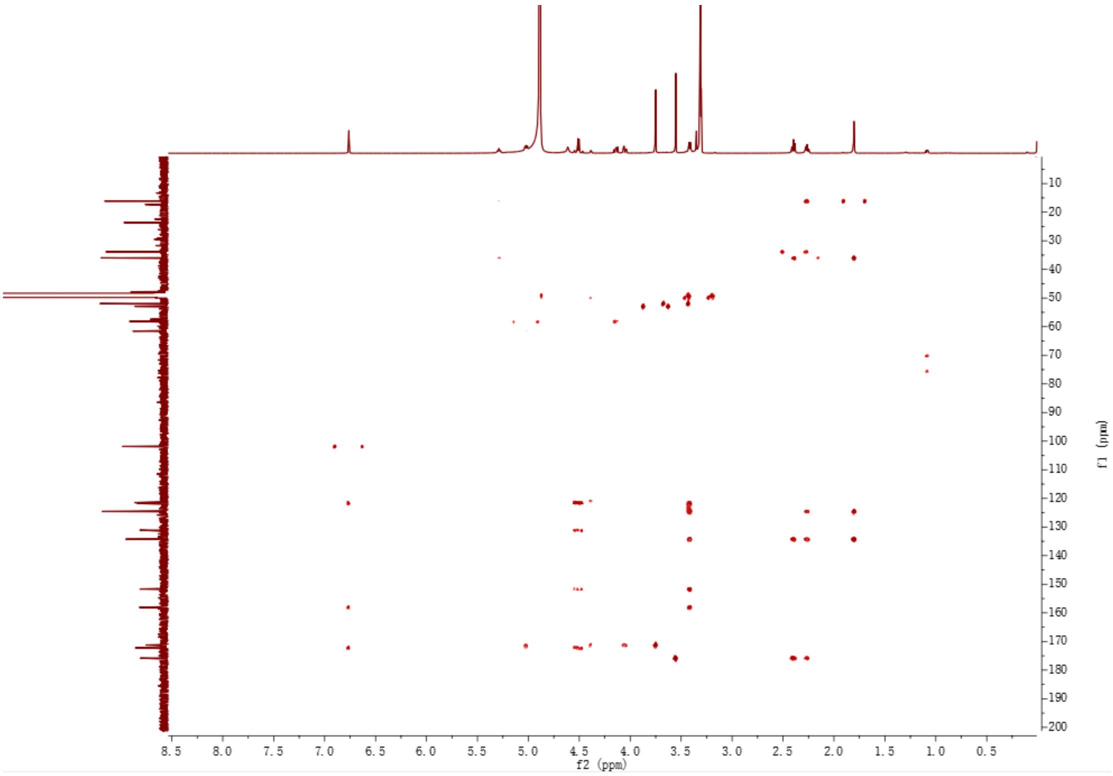

Figure S35. ROESY spectrum of erinacenone Q.

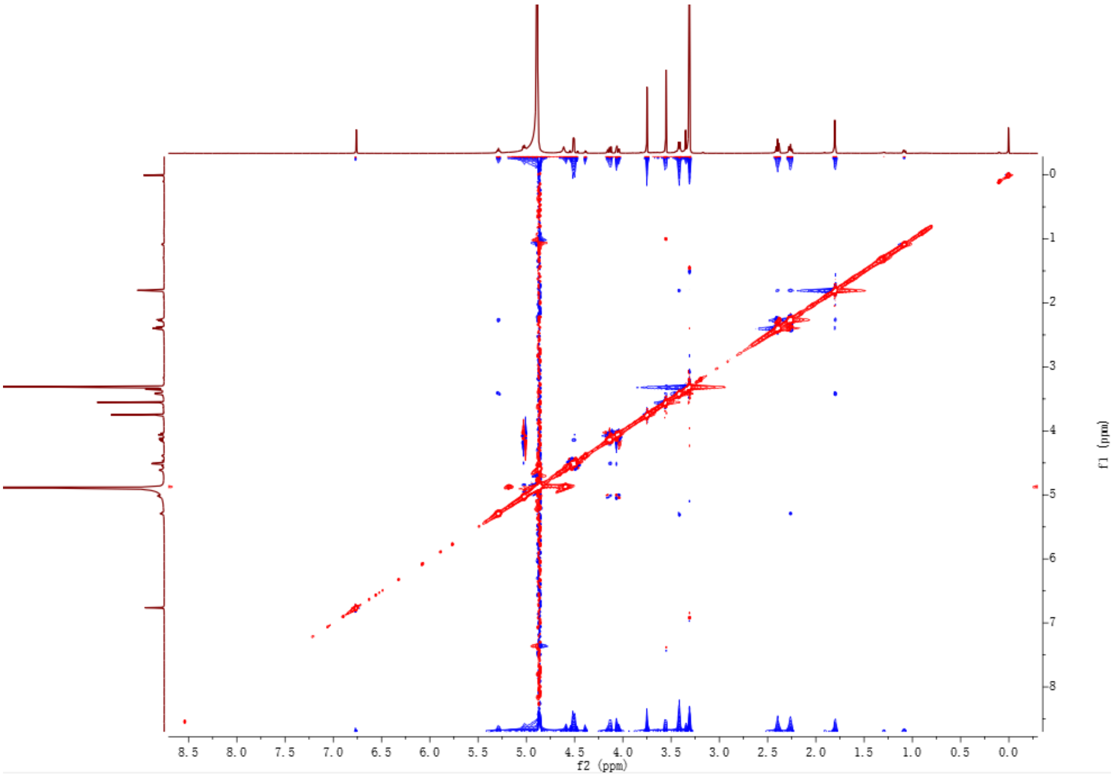

Figure S36. HRESIMS spectroscopic data of erinacenone R.

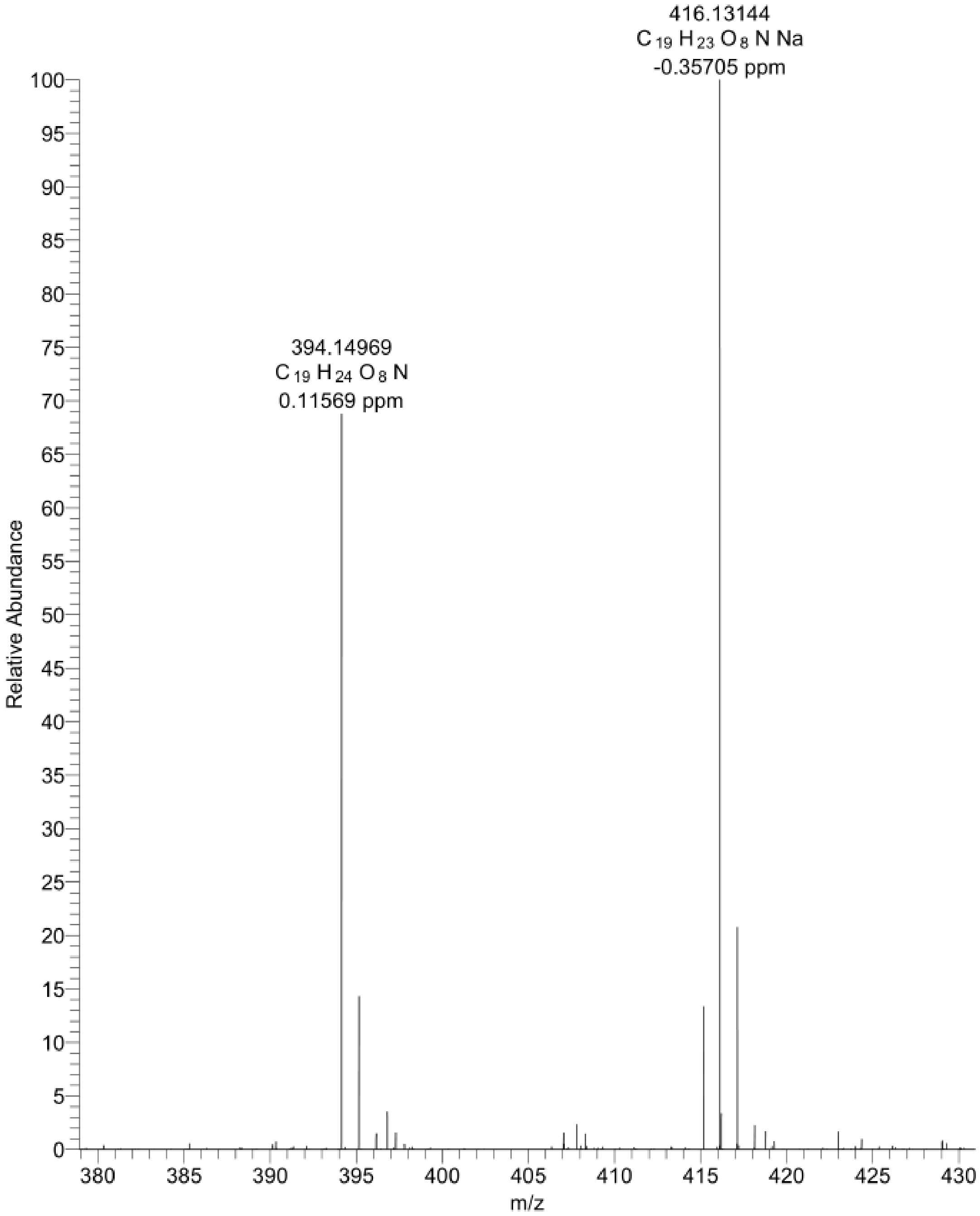

Figure S37. ^{1}H NMR (500 MHz) spectrum of erinacenone R.

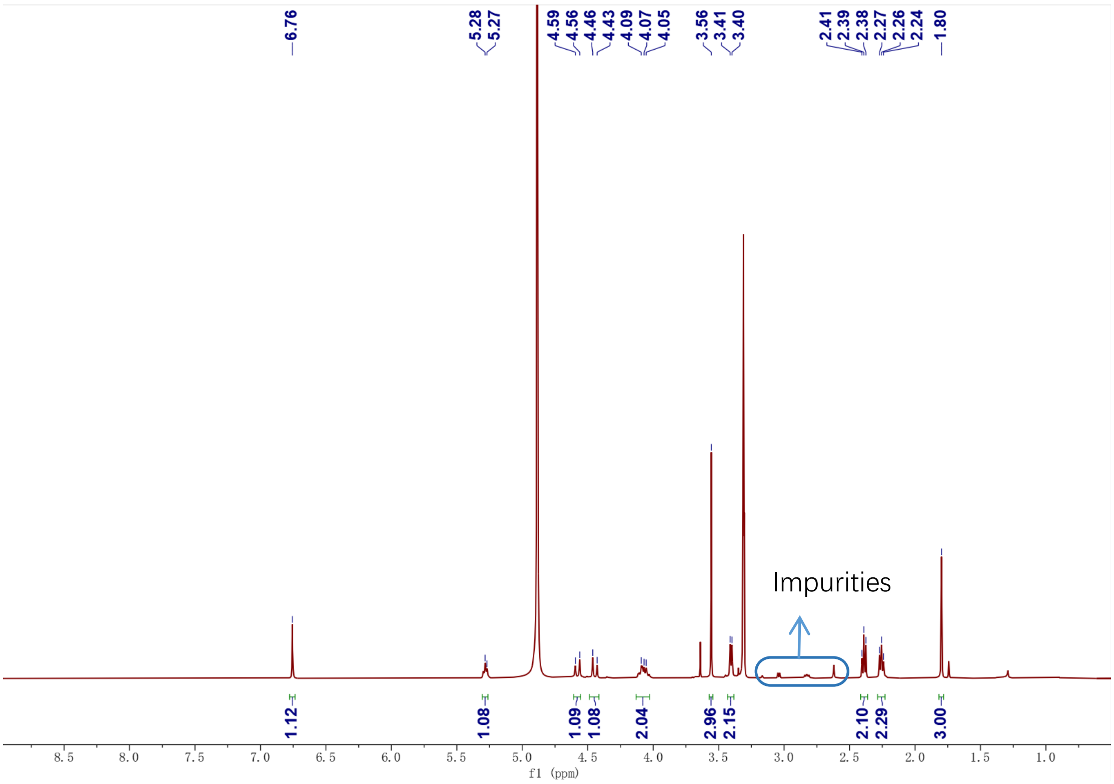

Figure S38. ^{13}C NMR and DEPT (125 MHz) spectra of erinacenone R.

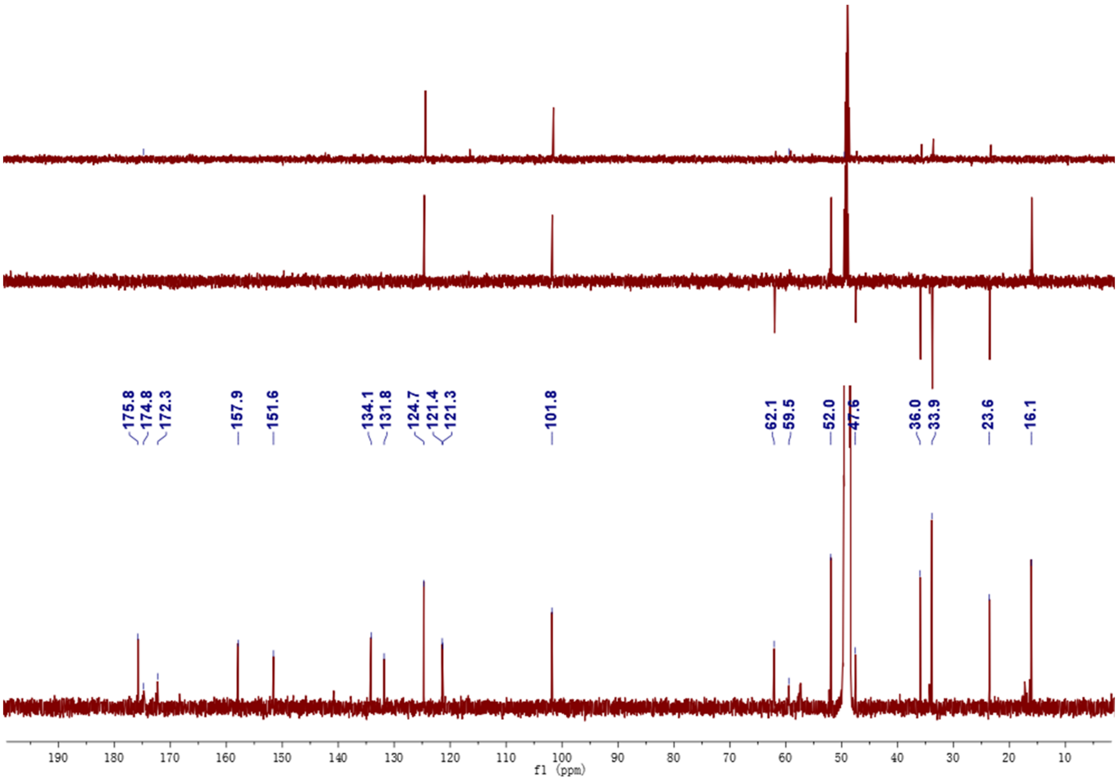

Figure S39. ^{1}H-^{1}H COSY spectrum of erinacenone R.

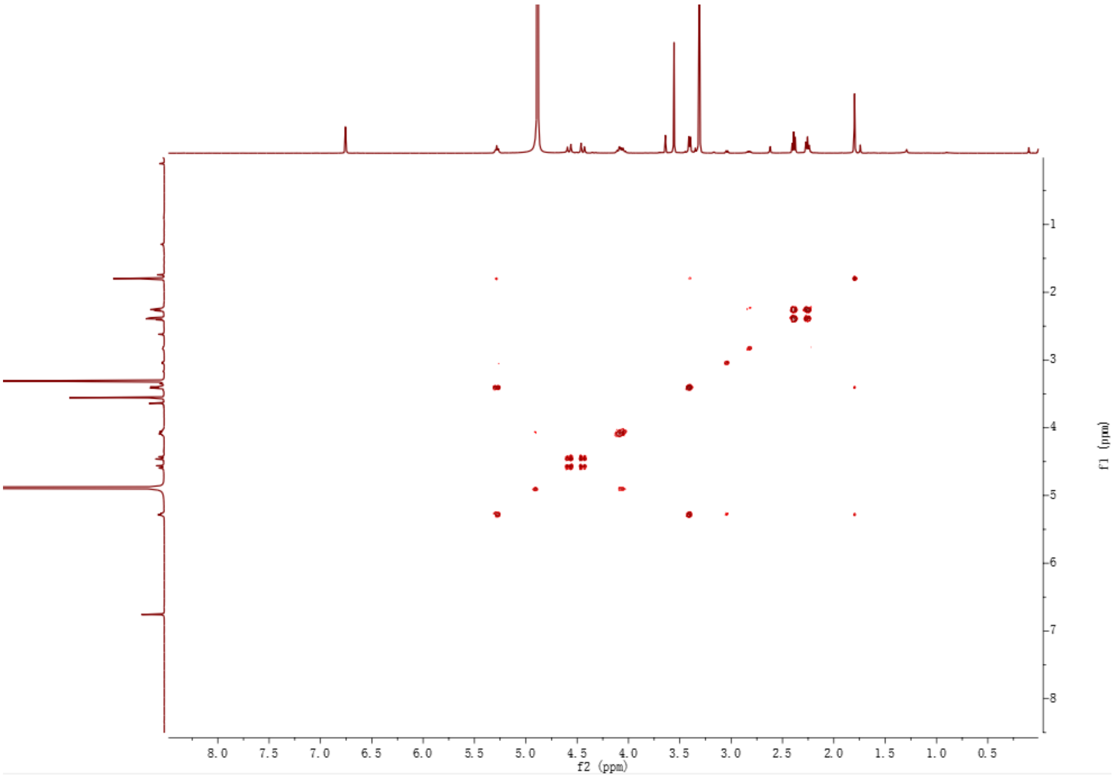

Figure S40. HSQC spectrum of erinacenone R.

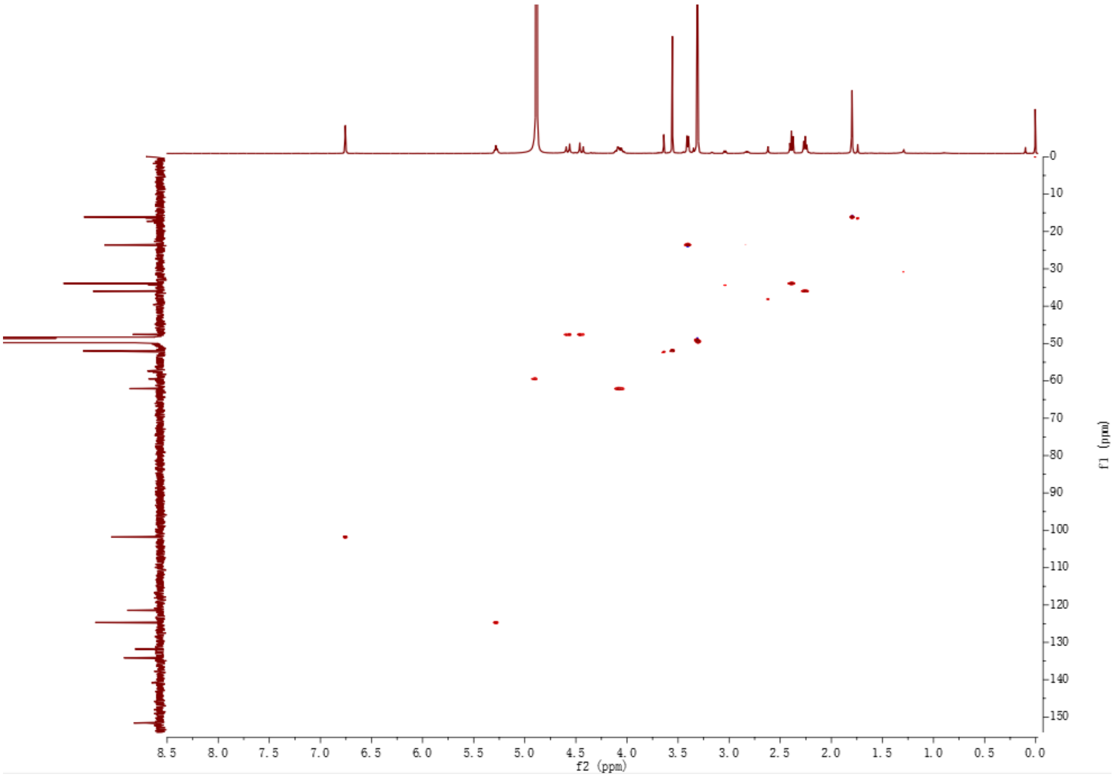

Figure S41. HMBC spectrum of erinacenone R.

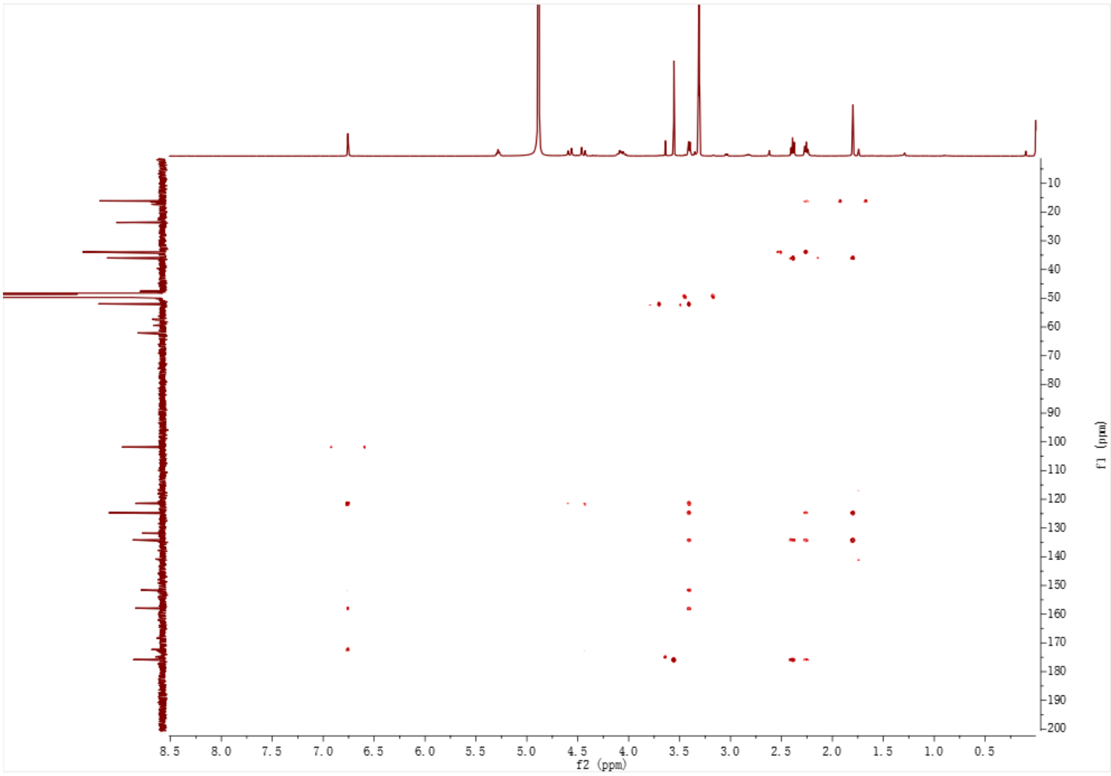

Figure S42. ROESY spectrum of erinacenone R.

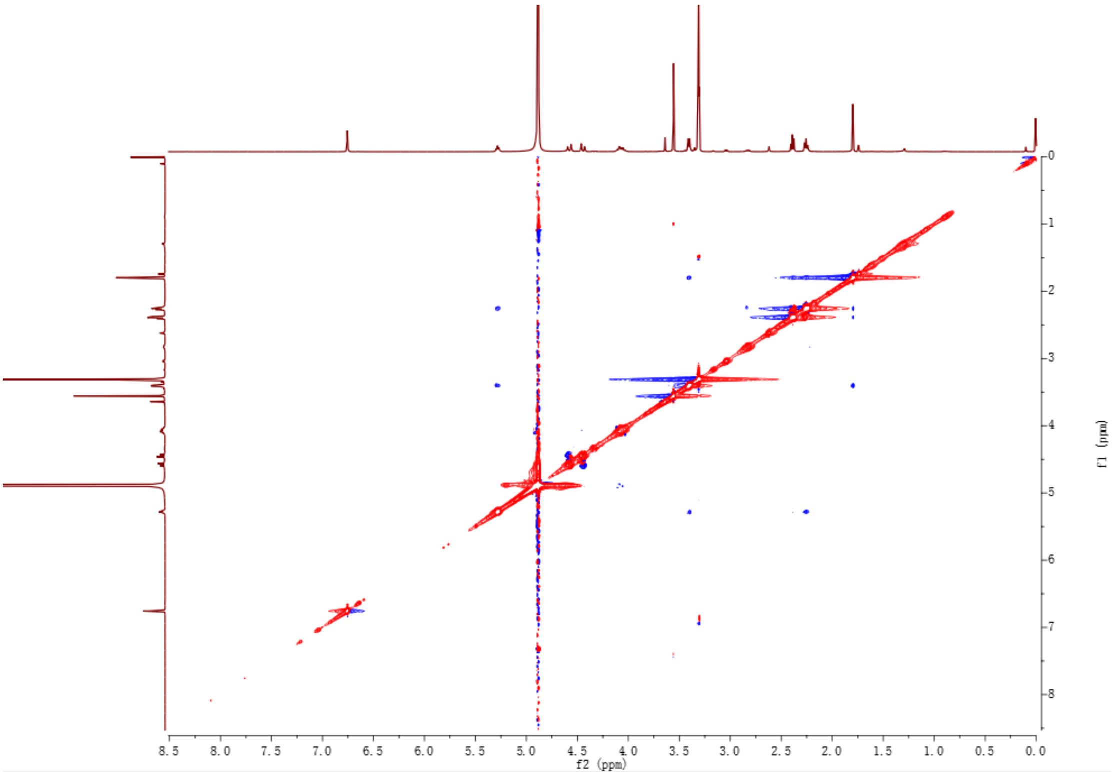

Figure S43. HRESIMS spectroscopic data of erinacenone S.

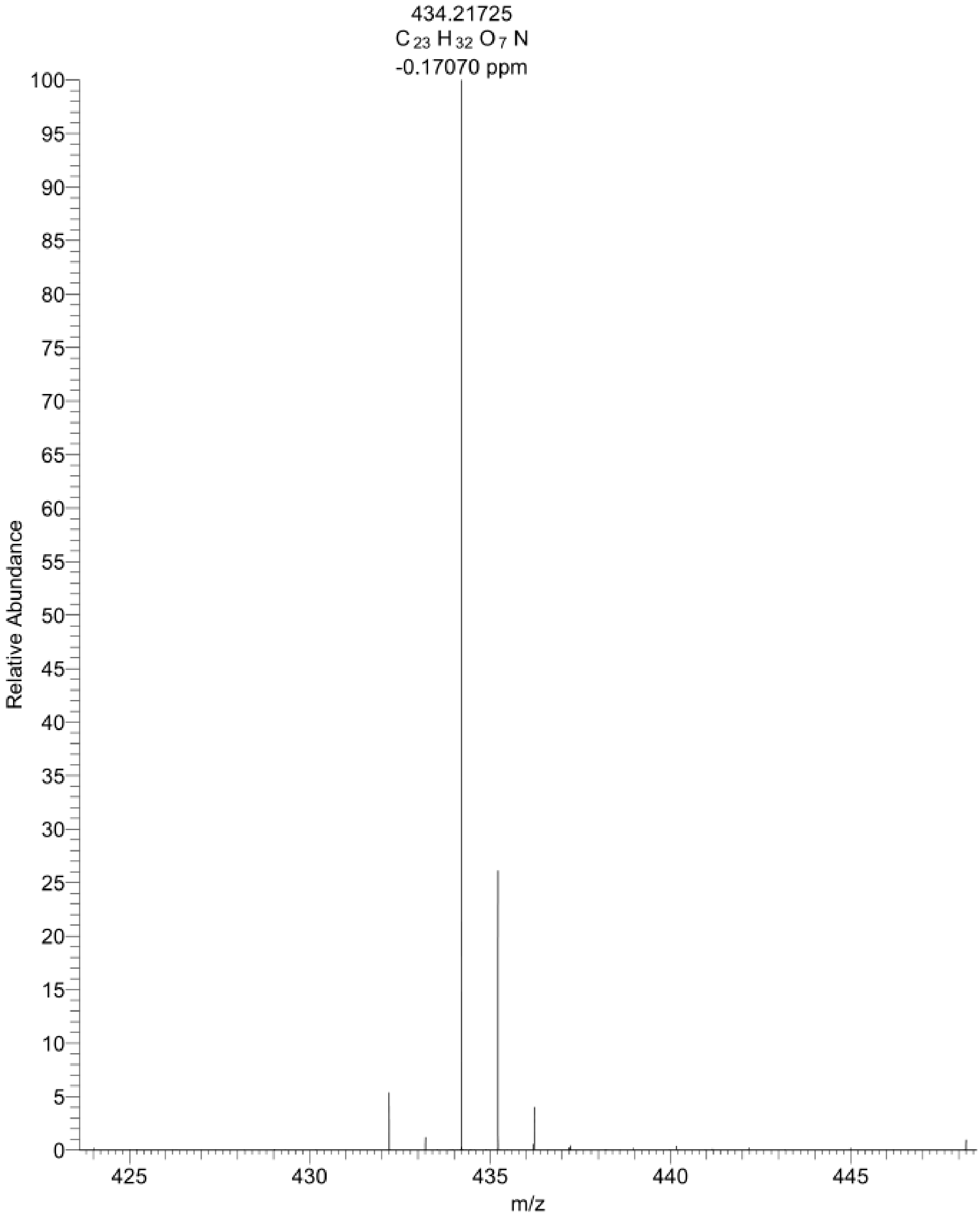

Figure S44. ^{1}H NMR (600 MHz) spectrum of erinacenone S.

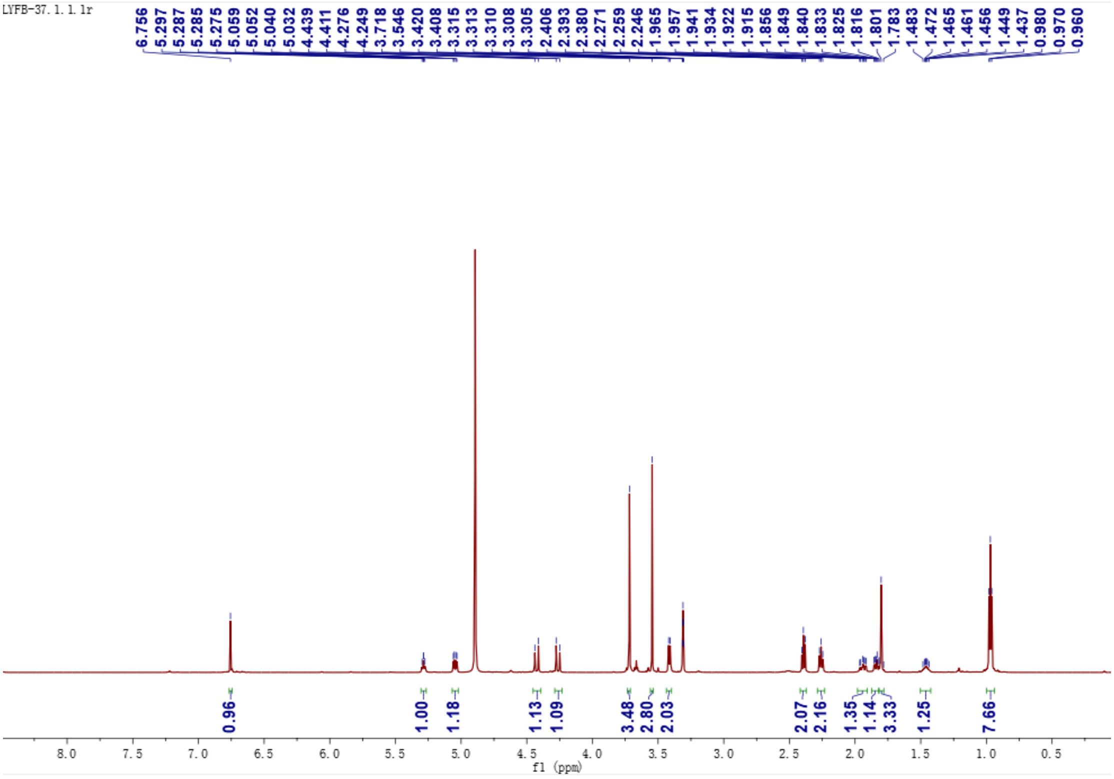

Figure S45. ^{13}C NMR and DEPT (150 MHz) spectra of erinacenone S.

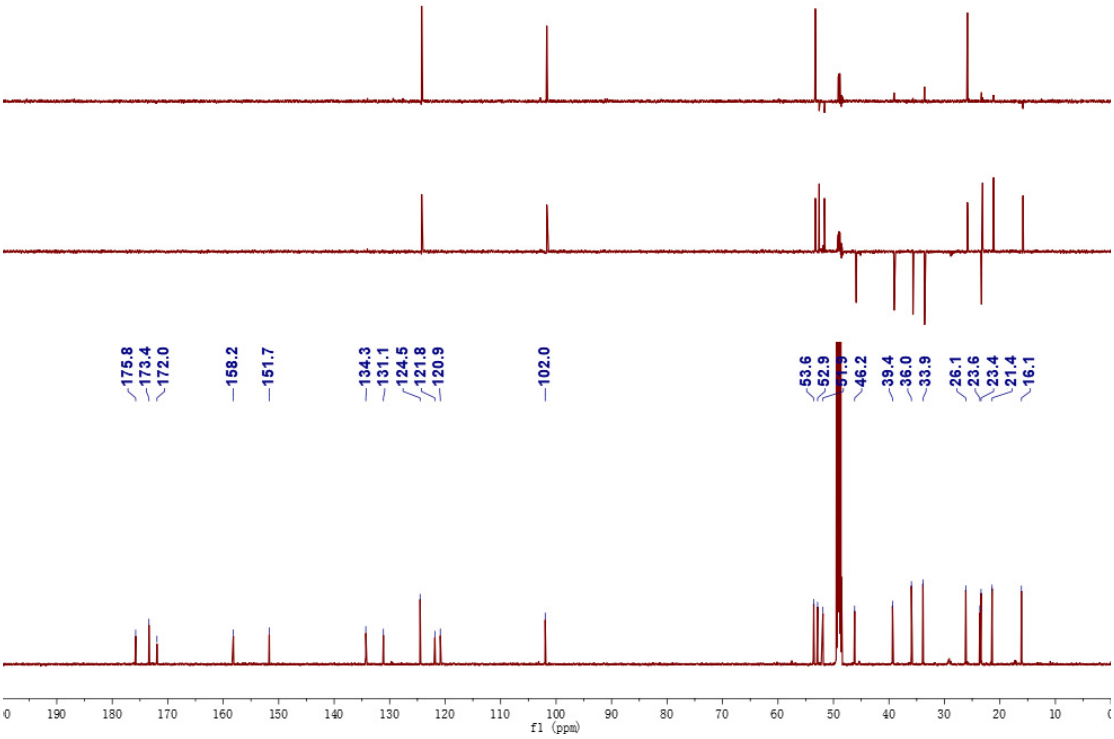

Figure S46. ^{1}H-^{1}H **COSY spectrum of erinacenone S.**

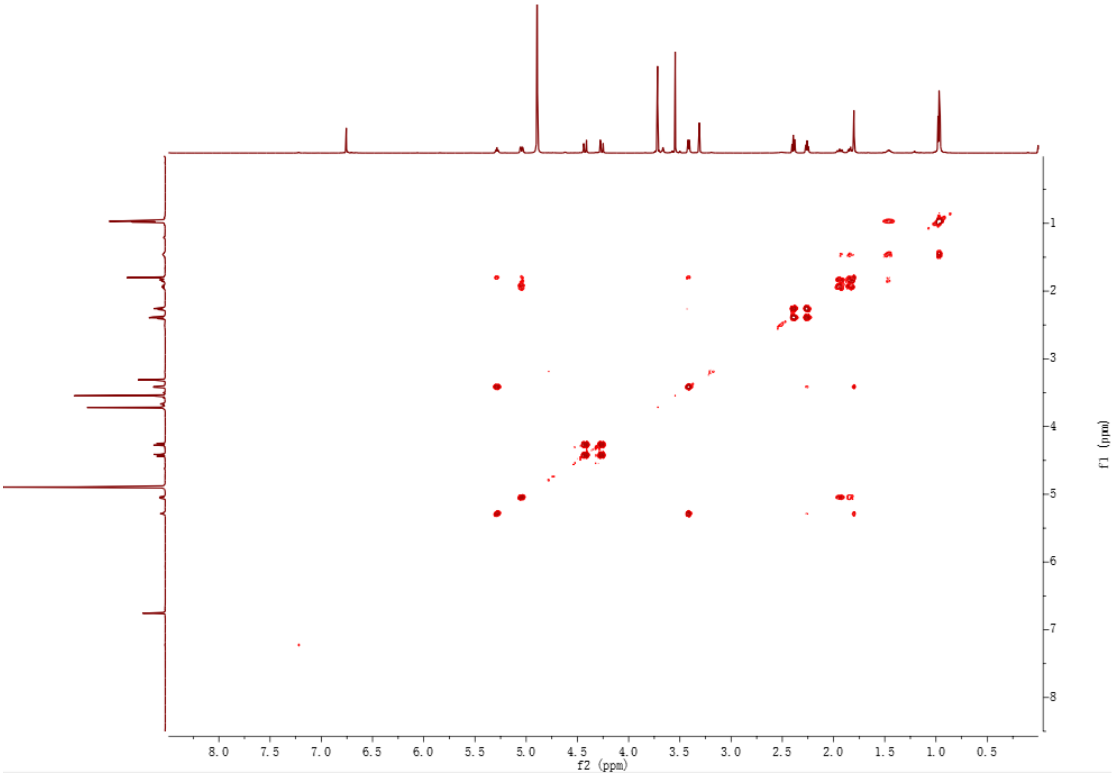

Figure S47. HSQC spectrum of erinacenone S.

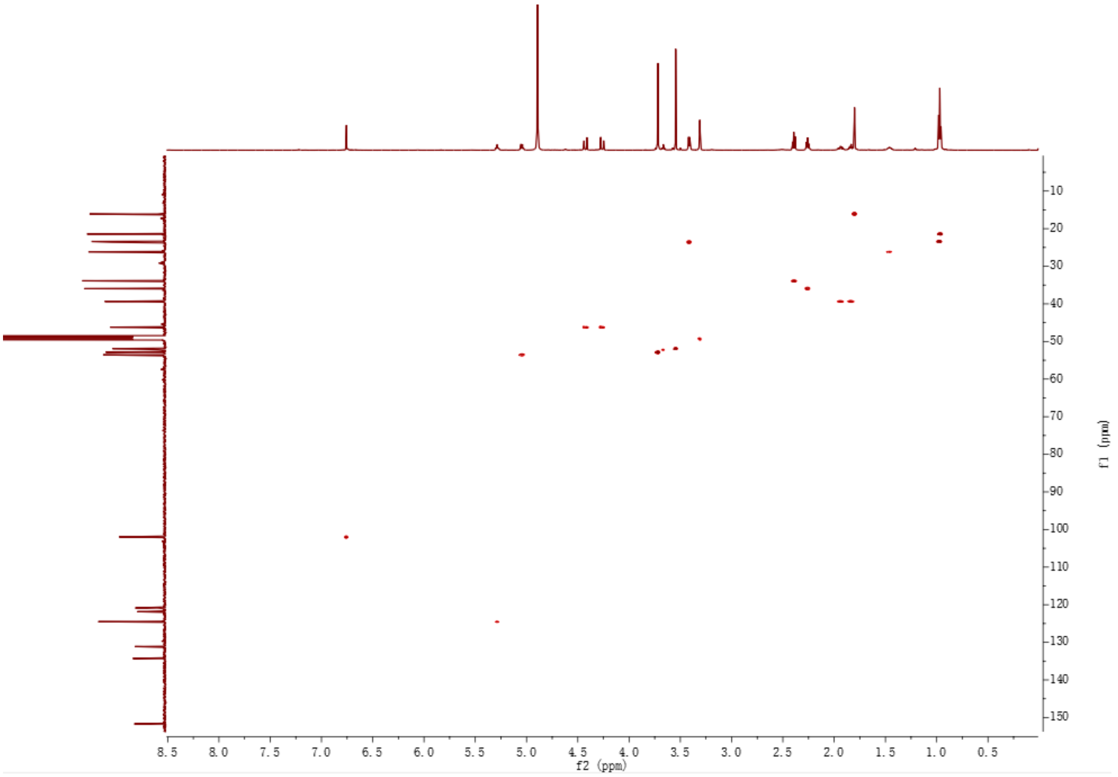

Figure S48. HMBC spectrum of erinacenone S.

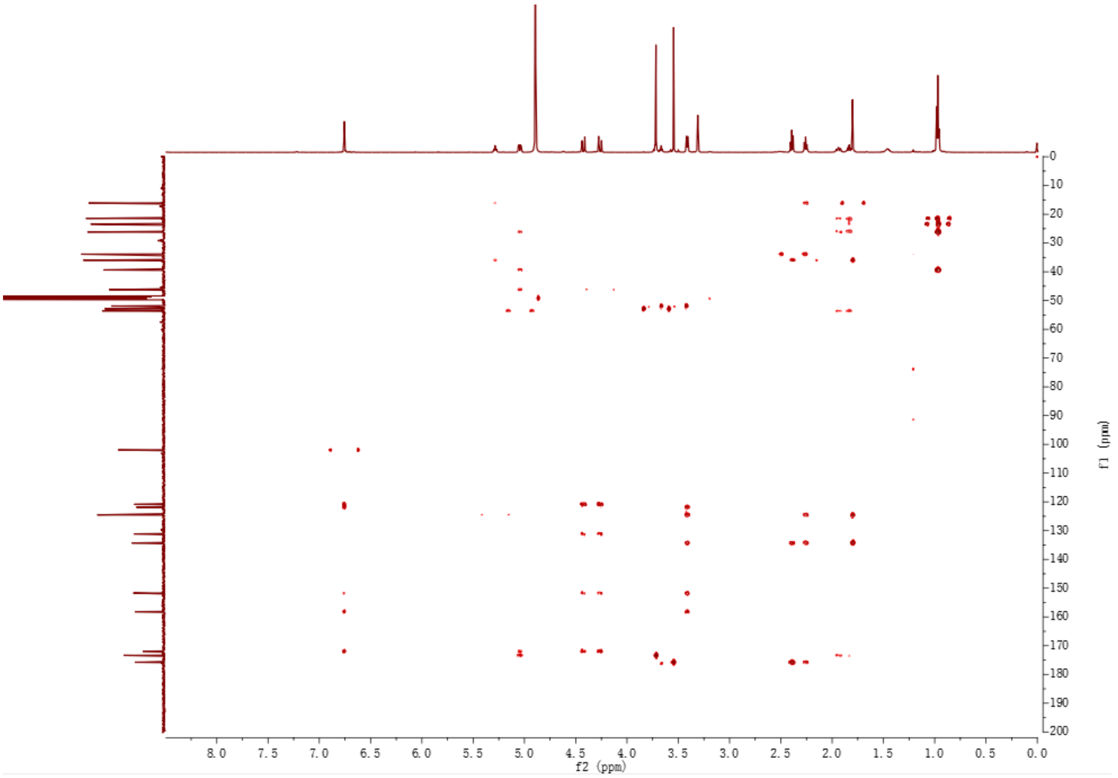

Figure S49. ROESY spectrum of erinacenone S.

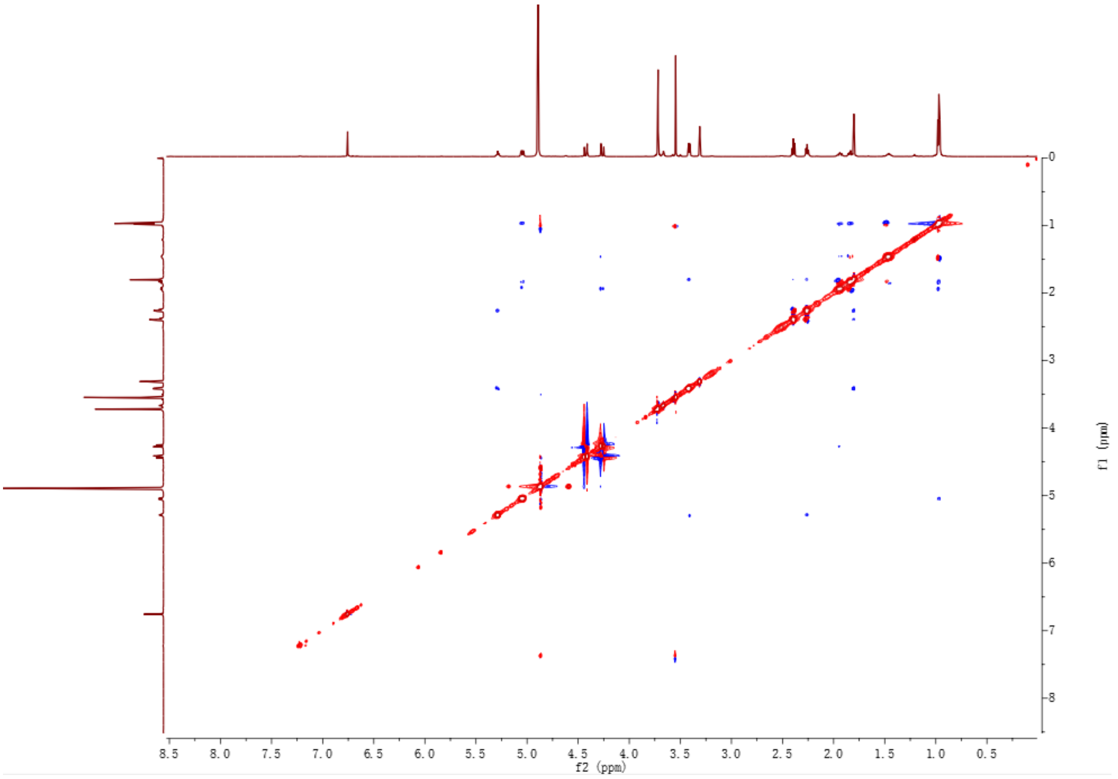

Figure S50. HRESIMS spectroscopic data of erinacenone T.

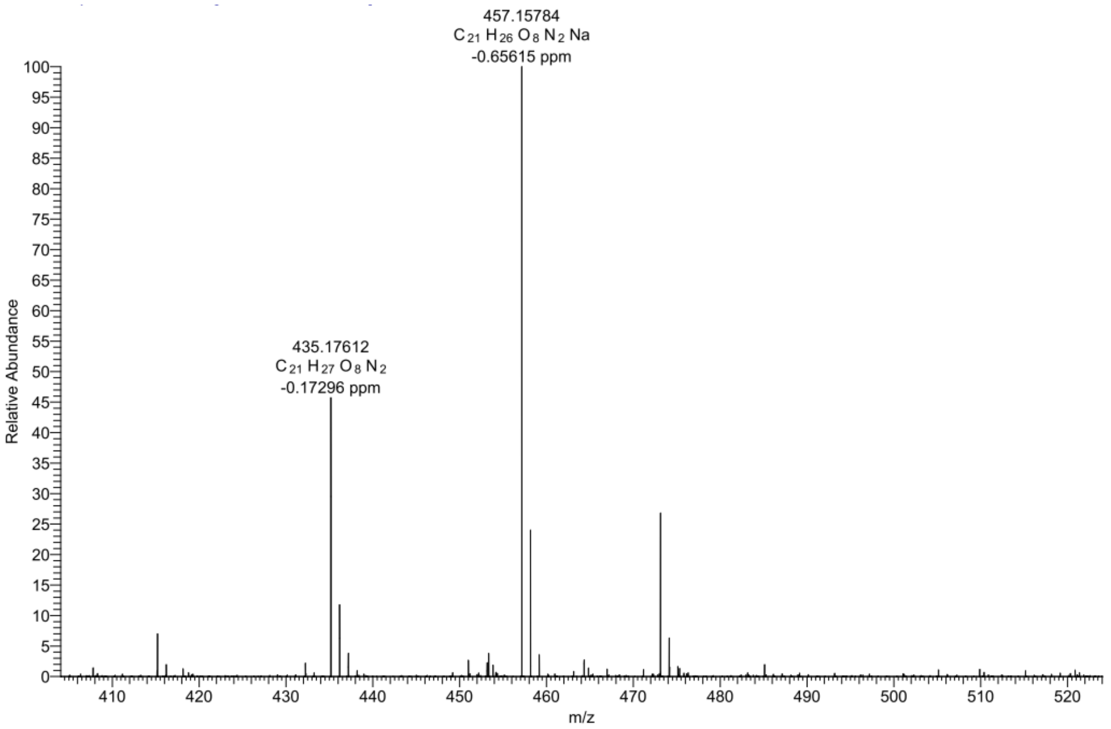

Figure S51. ^{1}H NMR (600 MHz) spectrum of erinacenone T.

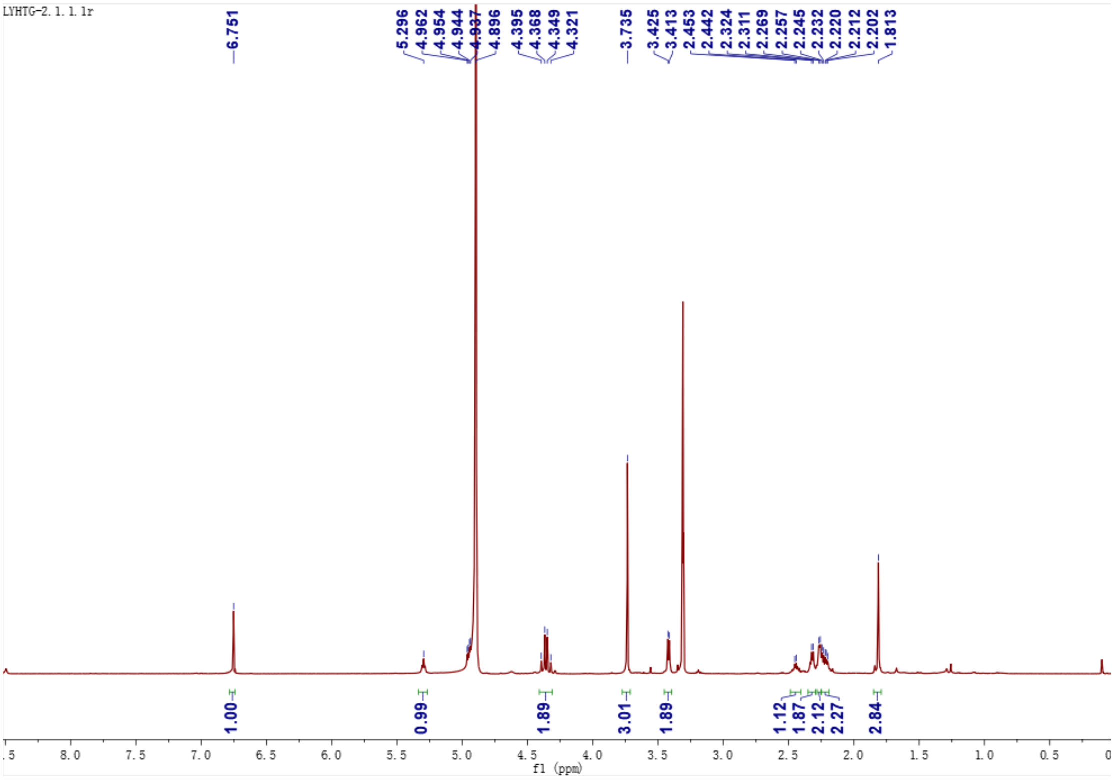

Figure S52. ^{13}C NMR and DEPT (150 MHz) spectra of erinacenone T.

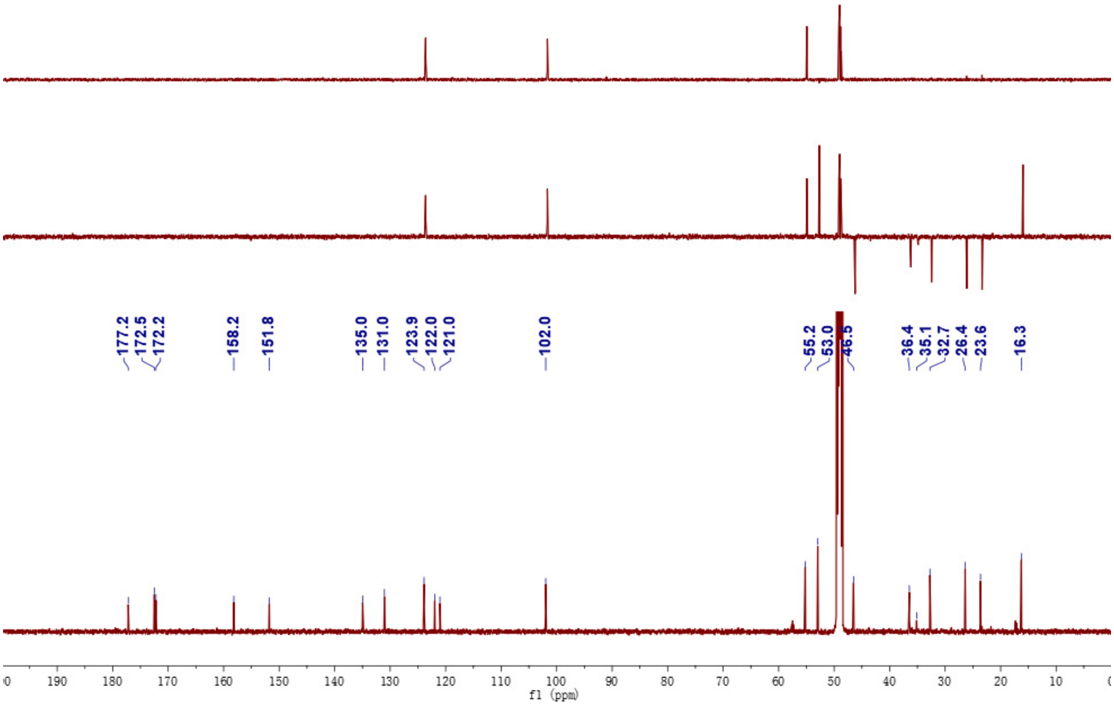

Figure S53. ^{1}H-^{1}H COSY spectrum of erinacenone T.

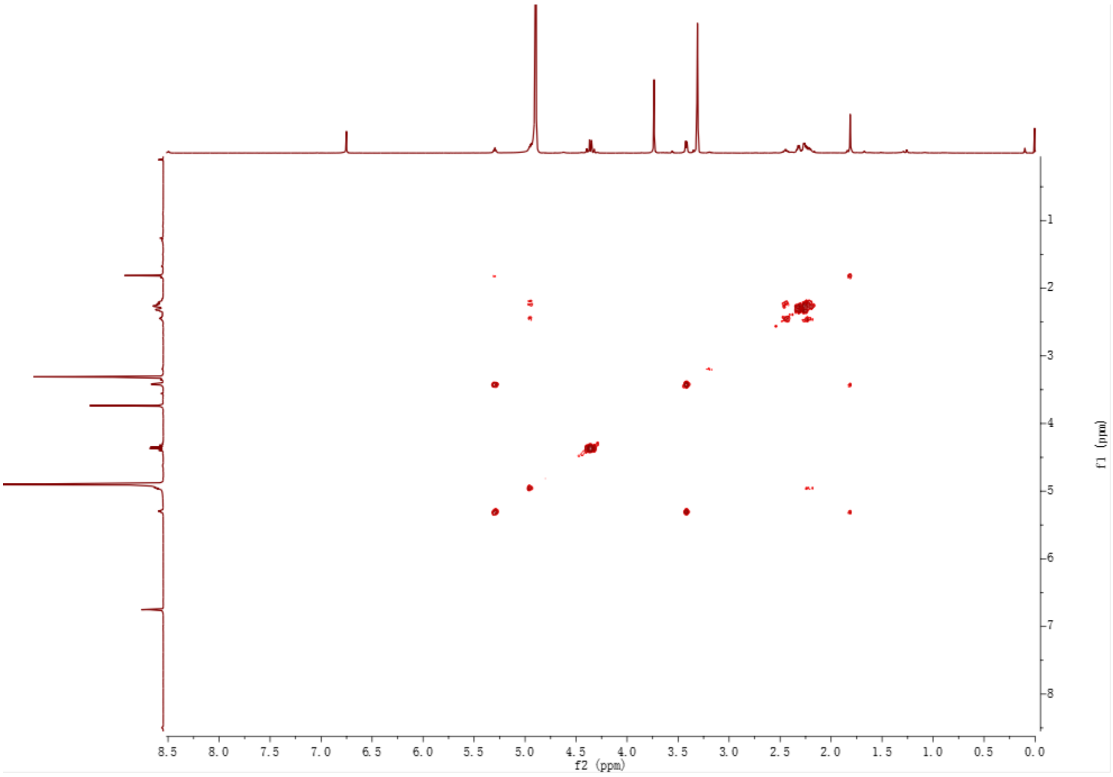

Figure S54. HSQC spectrum of erinacenone T.

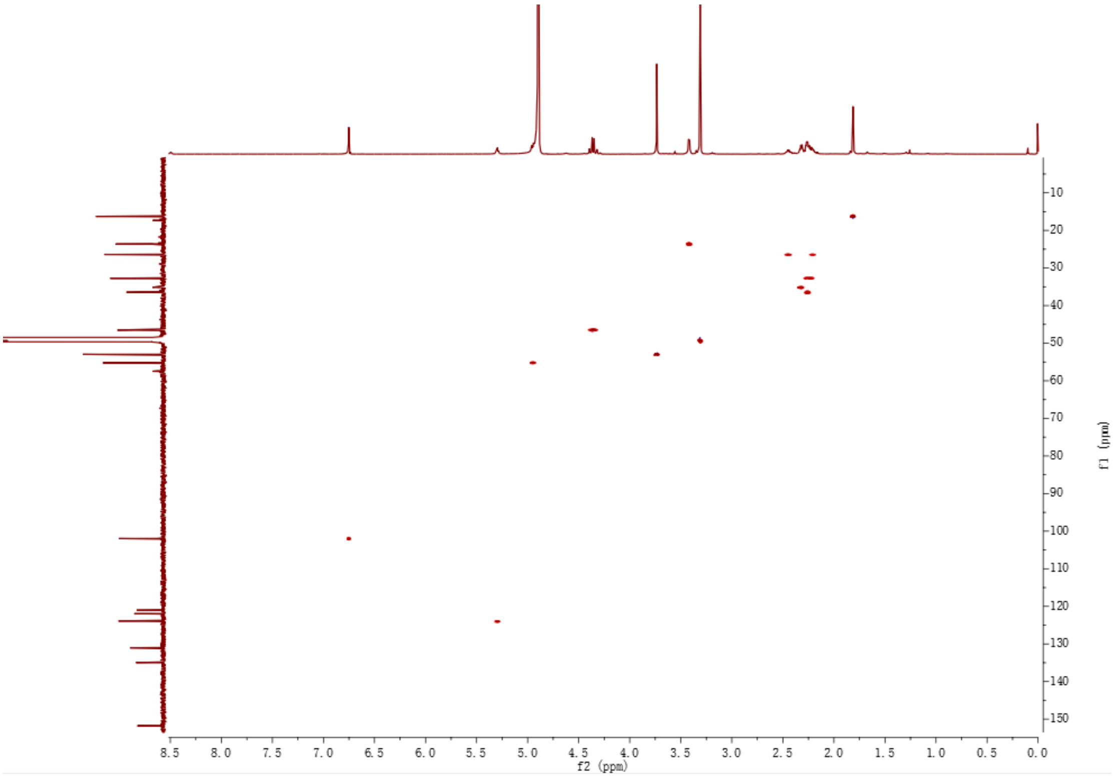

Figure S55. HMBC spectrum of erinacenone T.

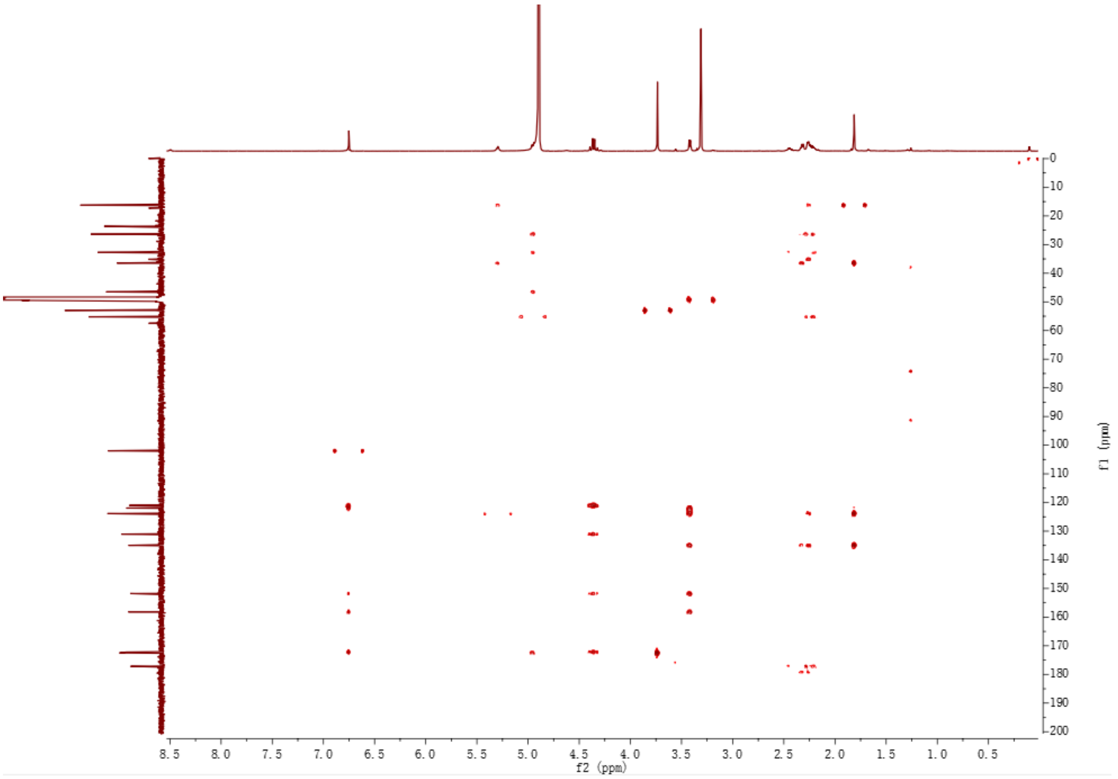

Figure S56. ROESY spectrum of erinacenone T.

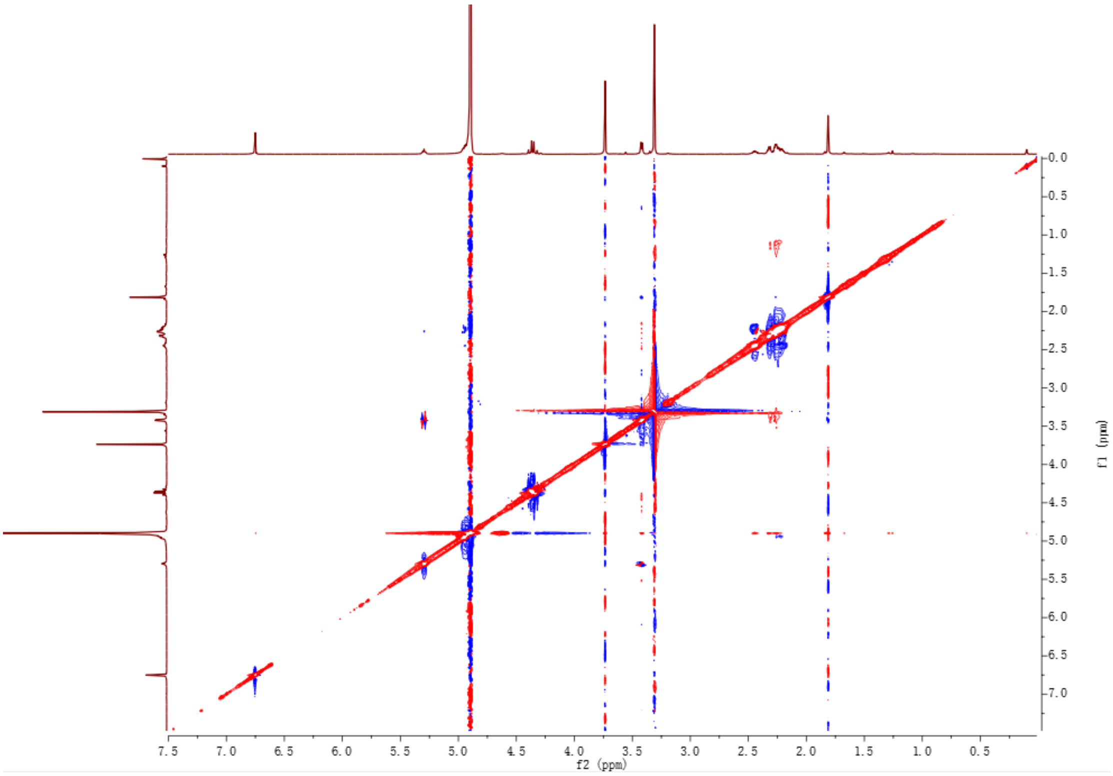

Figure S57. HRESIMS spectroscopic data of erinacenone U.

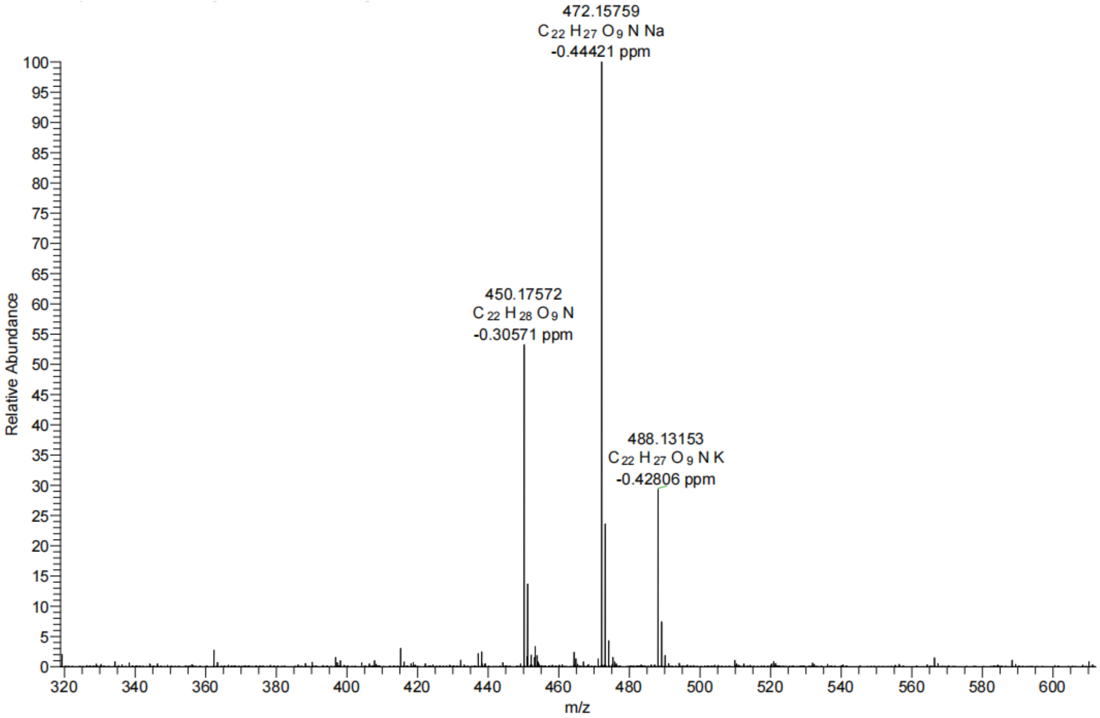

Figure S58. ^{1}H NMR (500 MHz) spectrum of erinacenone U.

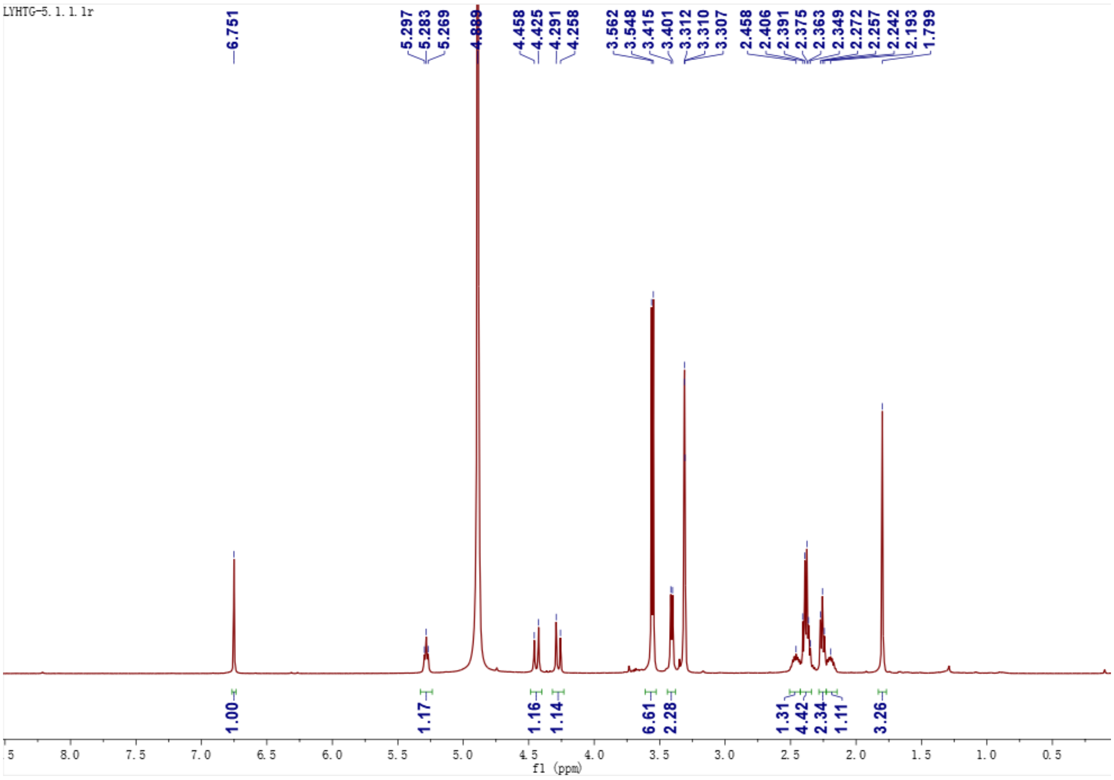

Figure S59. ^{13}C NMR and DEPT (125 MHz) spectra of erinacenone U.

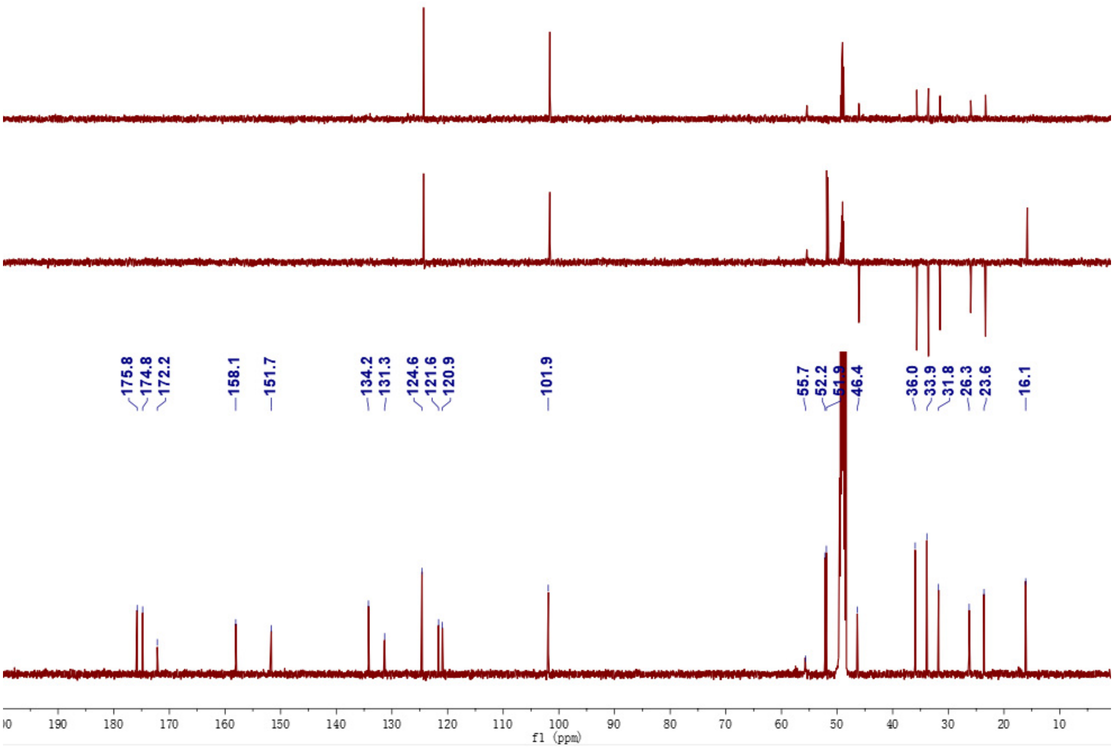

Figure S60. ^{1}H-^{1}H COSY spectrum of erinacenone U.

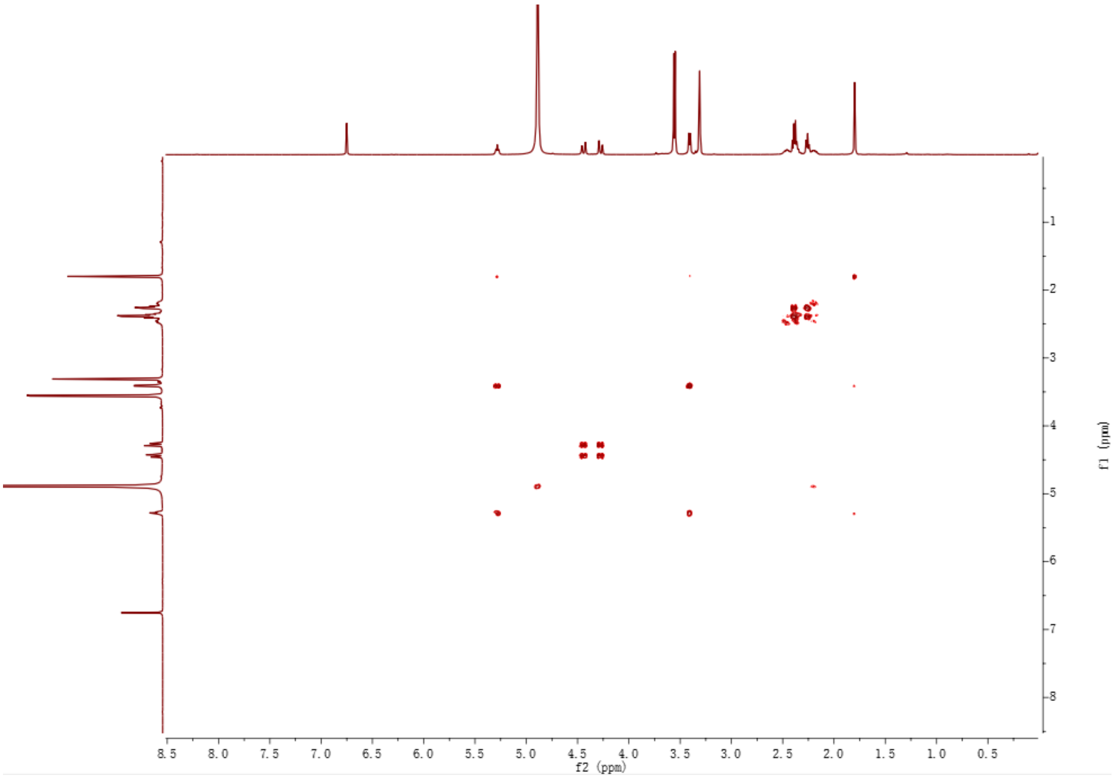

Figure S61. HSQC spectrum of erinacenone U.

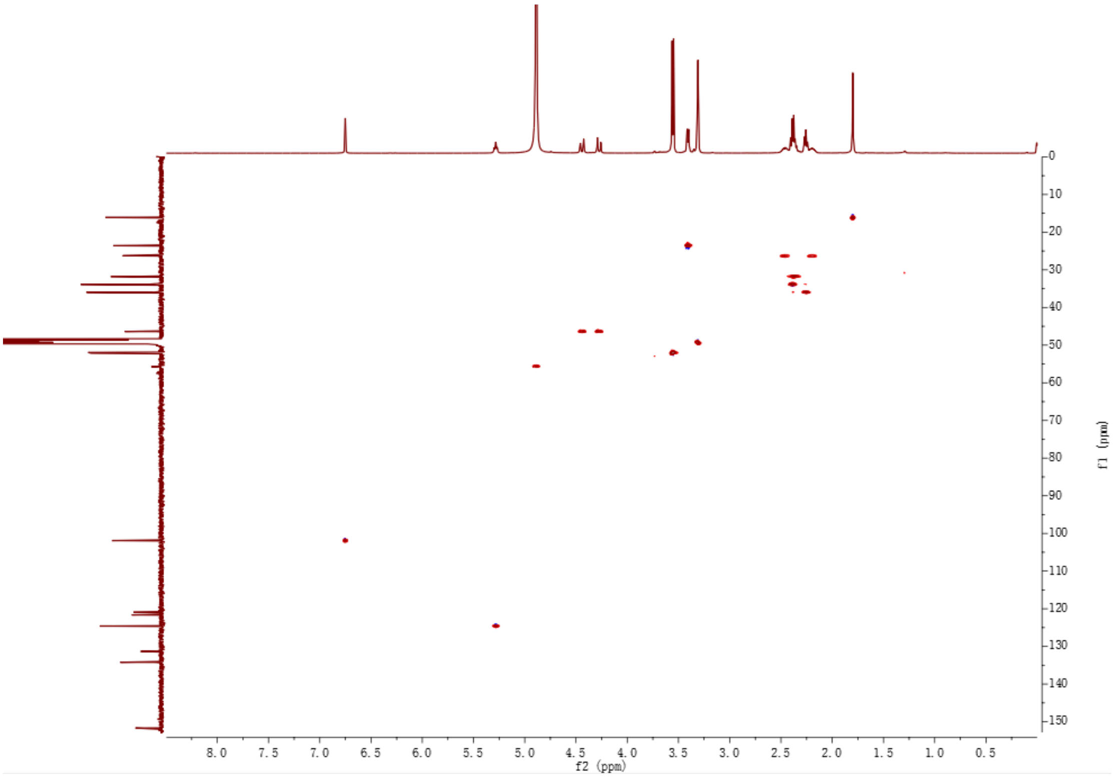

Figure S62. HMBC spectrum of erinacenone U.

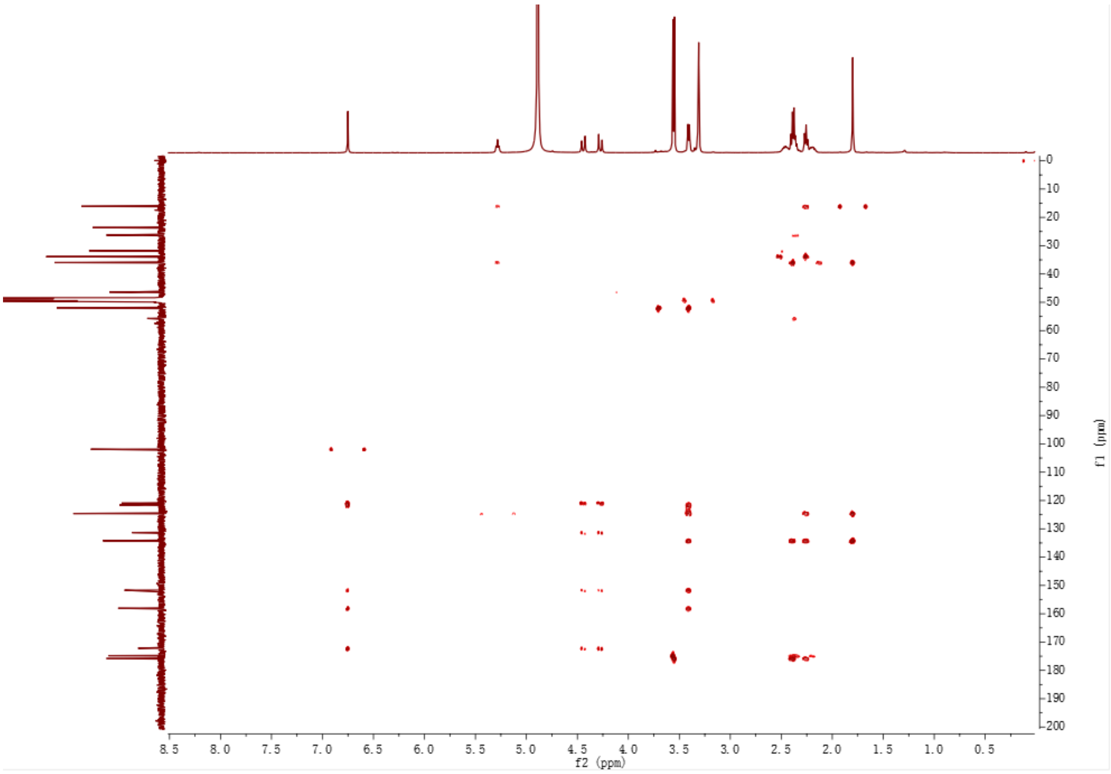

Figure S63. ROESY spectrum of erinacenone U.

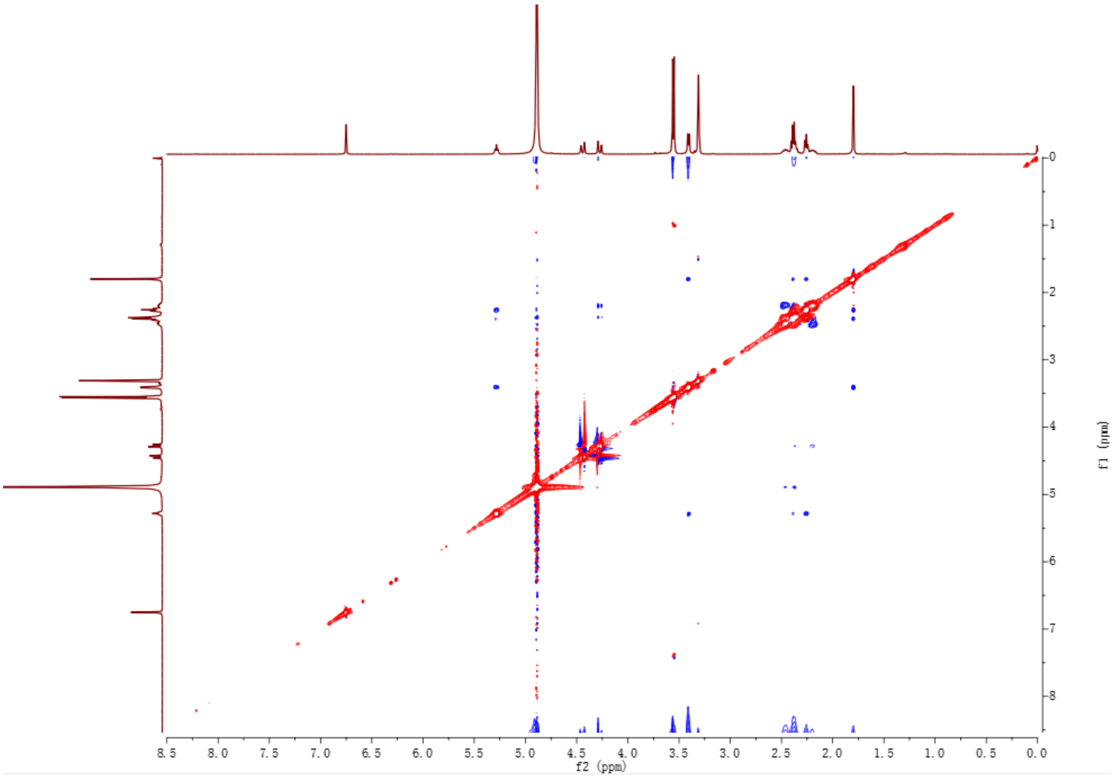

Figure S64. HRESIMS spectroscopic data of erinacenone V.

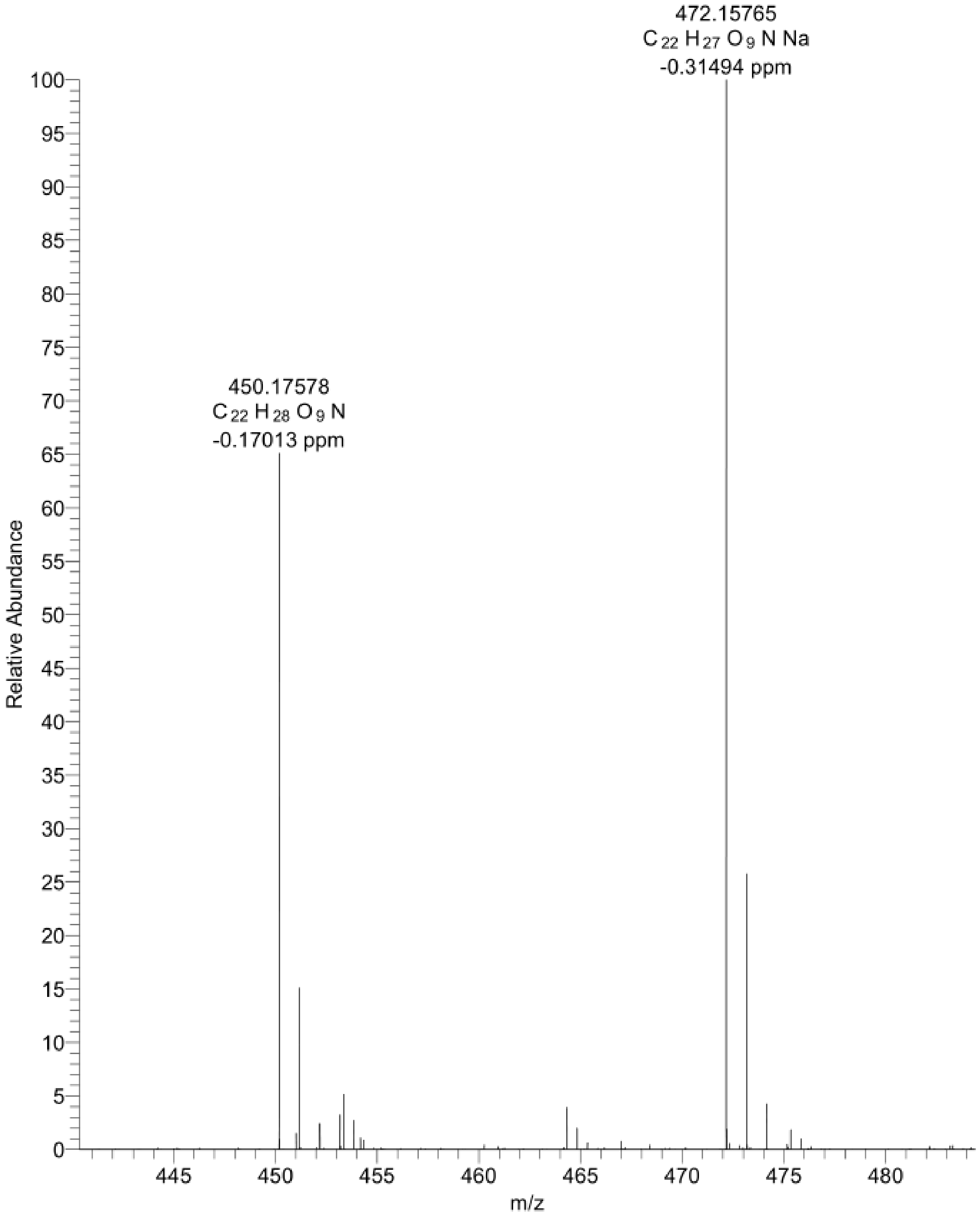

Figure S65. ^{1}H NMR (500 MHz) spectrum of erinacenone V.

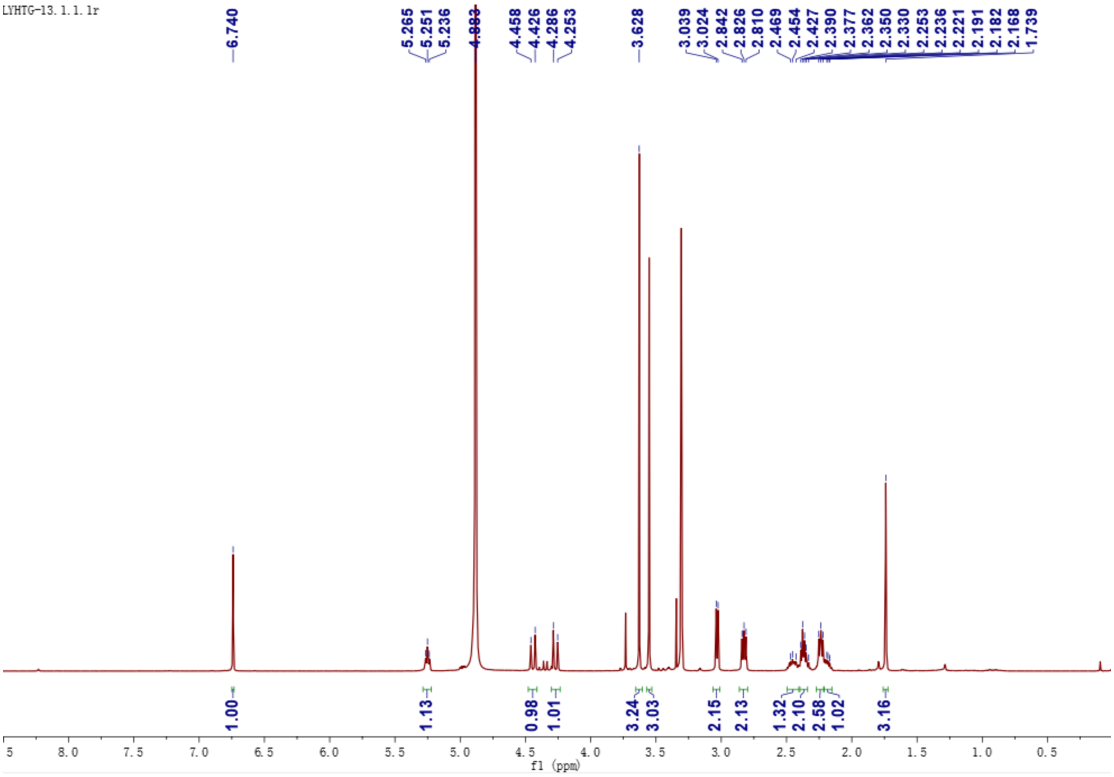

Figure S66. ^{13}C NMR and DEPT (125 MHz) spectra of erinacenone V.

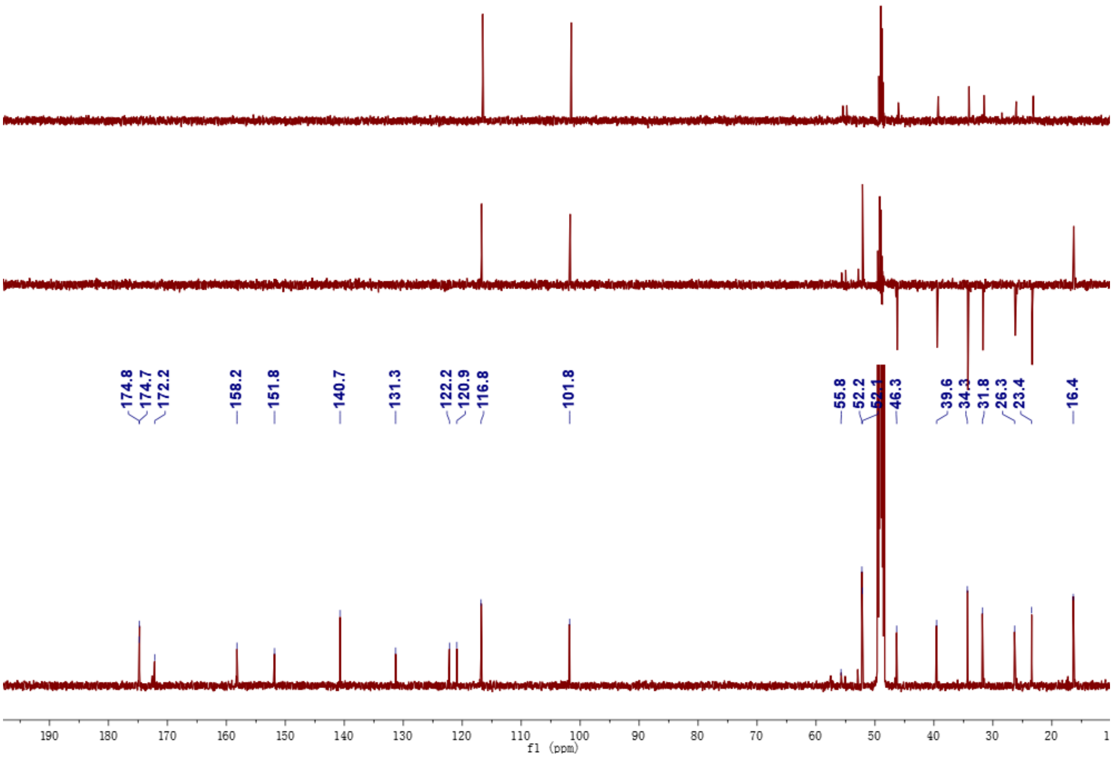

Figure S67. ^1H-^1H COSY spectrum of erinacenone V.

Figure S68. HSQC spectrum of erinacenone V.
